# Multiparametric Profiling of Engineered Nanomaterials: Unmasking the Surface Coating Effect

**DOI:** 10.1002/advs.202002221

**Published:** 2020-10-11

**Authors:** Audrey Gallud, Mathilde Delaval, Pia Kinaret, Veer Singh Marwah, Vittorio Fortino, Jimmy Ytterberg, Roman Zubarev, Tiina Skoog, Juha Kere, Manuel Correia, Katrin Loeschner, Zahraa Al‐Ahmady, Kostas Kostarelos, Jaime Ruiz, Didier Astruc, Marco Monopoli, Richard Handy, Sergio Moya, Kai Savolainen, Harri Alenius, Dario Greco, Bengt Fadeel

**Affiliations:** ^1^ Institute of Environmental Medicine Karolinska Institutet Stockholm 171 77 Sweden; ^2^ Faculty of Medicine and Health Technology Tampere University Tampere 33720 Finland; ^3^ Institute of Biotechnology University of Helsinki Helsinki 00790 Finland; ^4^ Institute of Biomedicine University of Eastern Finland Kuopio 70211 Finland; ^5^ Department of Medical Biochemistry & Biophysics Karolinska Institutet Stockholm 171 77 Sweden; ^6^ Department of Biosciences & Nutrition Karolinska Institutet Huddinge 141 83 Sweden; ^7^ National Food Institute Technical University of Denmark Kongens Lyngby 2800 Denmark; ^8^ Faculty of Biology Medicine & Health University of Manchester Manchester M20 4GJ UK; ^9^ School of Science & Technology Nottingham Trent University Nottingham NG1 8NS UK; ^10^ Catalan Institute of Nanoscience and Nanotechnology (ICN2) Barcelona 08193 Spain; ^11^ ISM UMR CNRS No. 5255 University of Bordeaux Talence 33 405 France; ^12^ Department of Pharmaceutical & Medicinal Chemistry Royal College of Surgeons in Ireland (RCSI) Dublin 2 Ireland; ^13^ School of Biological & Marine Sciences University of Plymouth Plymouth PL4 8AA UK; ^14^ Soft Matter Nanotechnology Laboratory CIC biomaGUNE Donostia‐San Sebastián 20014 Spain; ^15^ Finnish Institute of Occupational Health Helsinki 00032 Finland

**Keywords:** immunotoxicity, nanomaterials, nanotoxicology, proteomics, transcriptomics

## Abstract

Despite considerable efforts, the properties that drive the cytotoxicity of engineered nanomaterials (ENMs) remain poorly understood. Here, the authors inverstigate a panel of 31 ENMs with different core chemistries and a variety of surface modifications using conventional in vitro assays coupled with omics‐based approaches. Cytotoxicity screening and multiplex‐based cytokine profiling reveals a good concordance between primary human monocyte‐derived macrophages and the human monocyte‐like cell line THP‐1. Proteomics analysis following a low‐dose exposure of cells suggests a nonspecific stress response to ENMs, while microarray‐based profiling reveals significant changes in gene expression as a function of both surface modification and core chemistry. Pathway analysis highlights that the ENMs with cationic surfaces that are shown to elicit cytotoxicity downregulated DNA replication and cell cycle responses, while inflammatory responses are upregulated. These findings are validated using cell‐based assays. Notably, certain small, PEGylated ENMs are found to be noncytotoxic yet they induce transcriptional responses reminiscent of viruses. In sum, using a multiparametric approach, it is shown that surface chemistry is a key determinant of cellular responses to ENMs. The data also reveal that cytotoxicity, determined by conventional in vitro assays, does not necessarily correlate with transcriptional effects of ENMs.

## Graphical abstract

Through comprehensive profiling of a large panel of engineered nanomaterials (ENMs) using primary human macrophages and a human monocyte‐like cell line, the authors shed light on the importance of surface modifications of ENMs. These studies are a timely reminder that the biological (cellular) effects of ENMs including transcriptional changes may not be adequately reflected by conventional cytotoxicity assays.

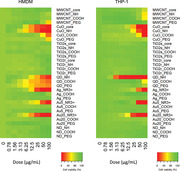

## 1 Introduction

Considerable progress has been made in recent years with respect to hazard assessment of engineered nanomaterials (ENMs), and advanced methods including high‐throughput and high‐content screening platforms as well as omics‐based approaches are gaining traction.^[^ ^1^ ^]^ However careful study design and appropriate data analysis tools are required to maximize the benefit of these emerging technologies.^[^ ^2^ ^]^ Indeed, despite the great promise of high‐throughput approaches,^[^ ^3^ ^]^ the limitation in most studies is the small number of ENMs, making it difficult to draw conclusions regarding the material properties that drive the biological responses to ENMs.^[^ ^4^ ^]^ Moreover, another challenge that is often overlooked is the fact that biology is complex. Indeed, it appears that toxicologists often apply the same “paradigm” for all ENMs rather than seeking to understand biological responses to ENMs in all their complexity (which is not to say that ENMs necessarily trigger novel responses—the pathways involved may very well be conserved).^[^ ^5^ ^]^ From a practical standpoint, there is a trade‐off between simple and robust assays which are amenable to high‐throughput screening^[^ ^6^ ^]^ versus comprehensive, omics‐based approaches that promise a wealth of information—though the latter studies may ultimately not be informative if the results are not validated. The seminal profiling study by Shaw et al.^[^ ^7^ ^]^ provides a case in point. The authors conducted high‐content screening of a large panel of ENMs of varying core compositions and surface modifications and were able to derive robust structure‐activity relationships by using this approach. The cell‐based assays applied were reflective of cell death and oxidative stress, two of the most commonly studied endpoints in nanotoxicology. Hence, by definition, such studies, though they provide a solid basis for hazard screening, cannot provide information on alternative cell fates, such as low‐dose and/or long‐term effects on cell function in the absence of cell death.^[^ ^8^, ^9^, ^10^ ^]^ Multi‐omics approaches, combining, for instance, proteomics and transcriptomics methods, coupled with bioinformatics analysis of the data, afford an opportunity to study the biological responses to ENMs in an unbiased manner, taking into account all the changes that occur at the level of protein and/or gene expression.^[^ ^11^, ^12^ ^]^ Such studies serve to complement, but cannot replace, toxicological studies using conventional assays.

In the present study, we conducted comprehensive in vitro testing of a panel of 31 ENMs, including cytotoxicity screening and cytokine profiling, using primary human monocyte‐derived macrophages (HMDMs) and the human monocytic THP‐1 cell line. Furthermore, proteomics and transcriptomics assessment of THP‐1 cells exposed to a low dose (EC_10_) of ENMs was performed along with detailed bioinformatics analysis of the data. Finally, validation experiments were performed to verify the impact of ENMs on specific cellular pathways. Taken together, these results have provided a systematic overview of nano–bio interactions and served to shed light on the role of chemical composition and surface modifications of ENMs. Our results also show that ENMs that appear biologically inert when assessed using conventional toxicity assays can still yield striking low‐dose effects on cells.

## 2 Results

### 2.1 Experimental Design

Faria et al.^[^ ^13^ ^]^ recently put forward a suggestion for a “minimum information standard” for publications on nano–bio interactions, focusing on three categories namely material characterization, biological characterization, and experimental protocols (in other words, materials, models, and methods). The aim is to improve reproducibility and to enable comparisons between publications in the field. The true value of such reporting requirements lies in their adoption by the scientific community and one should seek to place a reasonable burden on scientists in order to achieve compliance.^[^ ^14^ ^]^ In the present study, conducted in the frame of the European Commission‐funded NANOSOLUTIONS project, close attention was paid to the selection of materials, models, and methods. In brief, we prepared an extensive panel of ENMs comprising eight different core chemistries encompassing both metal, metal oxide, and carbon‐based materials and varying diameters and surface modifications, i.e., amino/ammonium‐, carboxyl/carboxylate‐, or poly(ethylene glycol) (PEG)‐terminated surfaces versus pristine ENMs. The three surface modifications provide stability to ENMs in aqueous media, while they convey different charges. Hence, amino/ammonium‐terminated coatings provide a positive charge, PEGylation is an example of neutral surface charge, and carboxyl/carboxylate coatings provide a negative charge. The purpose was to address the hypothesis that surface functionalization is the key driver of the biological (cellular) responses to ENMs. As our model, we employed primary HMDMs versus human monocyte‐like THP‐1 cells (nondifferentiated). Both cell types were maintained in the same cell culture medium in the presence of 10% fetal bovine serum (FBS). Cytotoxicity screening and cytokine profiling was performed in both models, while omics methods were applied only in THP‐1 cells. The reason for choosing a cell line as opposed to primary cells for omics was to minimize variability between experiments. Overall, the objective was to study the impact of ENMs on immune cells as the immune system represents the first barrier against foreign intrusion and previous studies have shown that monocytes/macrophages are particularly susceptible to ENMs.^[^ ^15^ ^]^ With regard to methods, we first explored the impact of the panel of ENMs by using conventional cell viability assays to establish a dose response and cytokine/chemokine arrays (using a subtoxic dose) followed by proteomics and transcriptomics assays coupled with detailed bioinformatics analyses. It is important to note that the cells were exposed to equipotent doses (i.e., EC_10_) for the cytokine and omics profiling studies to allow for a comparison between the ENMs (with minimal variations in cell death). Functional assays were then performed to verify the microarray results.

### 2.2 ENM Characterization

ENMs of different chemical composition were prepared with three types of surface functionalization: carboxyl/carboxylate groups (COOH/COO^−^), amino/ammonium groups (−NH_2_/−NR^3+^) or PEG. Additionally, in the case of TiO_2_ spheres and rods, CuO NPs, and multiwalled carbon nanotubes (MWCNTs), nonfunctionalized variants (designated hereafter as “core” ENMs) were synthesized. ENMs were subjected to a thorough characterization of physicochemical properties (Table S1, Figures S1 and S2, Supporting Information). Morphology and primary particle size were determined by using transmission electron microscopy (TEM) (Figure S1a–h, Supporting Information). The spherical and rod‐shaped features of the two different TiO_2_ NPs could be clearly distinguished. Dynamic light scattering (DLS) measurements showed, overall, that ENMs in suspension were in the range of 20–300 nm in diameter, except CuO‐core and CuO—COOH displaying an average hydrodynamic diameter size of about 1 µm, and the degree of agglomeration was found to be acceptable for all ENMs with PDI values < 0.5. However, DLS measurements were not conducted for MWCNTs, and the results were unreliable for the very small ENMs including Au NPs, quantum dots (QDs), and nanodiamonds (NDs) (≤ 5 nm). Zeta potential measurements showed, overall, that the surface charge was altered with the surface chemistry (in Milli‐Q water). MWCNTs, Ag NPs, Au NPs, and QDs were also characterized by using ultraviolet (UV)–visible spectroscopy, and QDs were evaluated with photoluminescence spectroscopy. The photoluminescence spectra shown in Figure S2f (Supporting Information) revealed that the amino‐modified QDs did not display fluorescent properties, unlike the other QDs. MWCNTs were also examined with Raman spectroscopy and the characteristic D and G bands were clearly observed (Figure S2g, Supporting Information). In addition, X‐ray photoelectron spectroscopy (XPS) and Fourier transform infrared spectroscopy (FTIR) (data not shown) confirmed the presence of the expected functional groups on the different ENMs. Overall, our analysis confirmed the expected properties of the 31 ENMs.

ENMs were then evaluated for possible endotoxin content by using the Limulus Amebocyte Lysate (LAL) chromogenic assay.^[^ ^16^ ^]^ In the case of MWCNTs and Au ENMs, interference with the LAL assay was noted. To overcome this issue, we used the macrophage‐based TNF‐*α* expression test (TET), as described.^[^ ^17^ ^]^ High levels of TNF‐*α* secretion were found in macrophages exposed to Au—COOH (5 nm) and Au—COOH (20 nm), and this was blocked by polymyxin B, indicating the presence of endotoxin (data not shown). Therefore, new carboxylated Au ENMs were prepared and these were endotoxin‐free (< 0.5 EU). The subsequent experiments were performed with endotoxin free ENMs.

### 2.3 Cytotoxicity Screening

The ENMs were evaluated for cytotoxicity using HMDMs and the undifferentiated monocytic THP‐1 cell line. To this end, cells were exposed for 24 h to freshly dispersed ENMs at doses up to 100 µg mL^−1^ and the Alamar Blue assay was applied as a proxy for cell viability (based on cellular metabolic activity).^[^ ^18^ ^]^ For HMDMs, the data were generated with cells derived from three individual donors (each experiment performed in triplicate). As shown in Figure S3 (Supporting Information), a greater cytotoxicity was observed for CuO‐core and CuO—NH_2_ in comparison to CuO—COOH and CuO—PEG. Regarding MWCNTs, only MWCNT–PEG induced cell death, around 50% at the highest concentration. For TiO_2_ NPs, neither spheres nor rods affected cell viability, while for CdTe QDs, all were found to be cytotoxic irrespective of the surface modification. Furthermore, for Ag NPs and Au NPs (5 and 20 nm), only the ENMs with ammonium surface functionalization were cytotoxic. Finally, no effect on cell viability was observed for the NDs.

**Figure 1:**
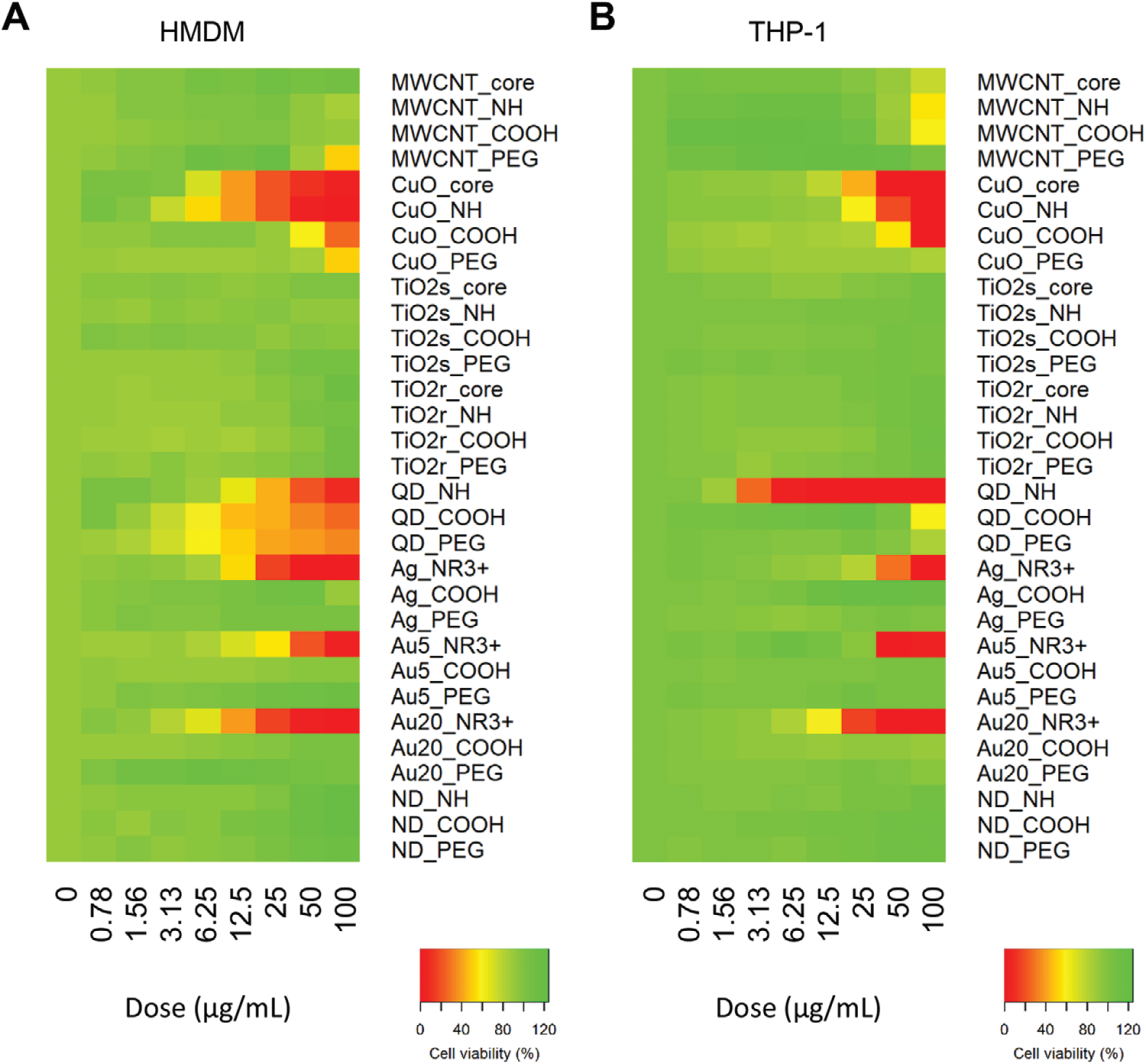
Cytotoxicity screening showed good concordance between primary macrophages and the monocyte‐like cell line. Heatmaps of cytotoxicity results obtained using A) HMDMs versus B) THP‐1 cells A). The percentages of cell viability following 24 h exposure to the 31 ENMs (see Table S1, Figures S1 and S2, Supporting Information) are shown; the corresponding cytotoxicity results obtained using HMDMs and THP‐1 cells are reported in Figures S3 and S4 (Supporting Information). The results are based on experiments using cells from three different donors (HMDMs) or biological replicates (THP‐1), each experiment performed in triplicate.

For the THP‐1 cell line, the data obtained were from three independent experiments each performed in triplicate (Figure S4, Supporting Information). Regarding CuO NPs, CuO‐core and CuO—NH_2_ induced a greater cytotoxicity in comparison to CuO—COOH, while CuO—PEG had a minor impact on cell viability only at the highest concentration. MWCNT‐core, —NH_2_, and —COOH were found to be cytotoxic for THP‐1 cells exposed to 50 or 100 µg mL^−1^, while MWCNT‐PEG did not affect cell viability. Neither TiO_2_ spheres/rods nor the NDs affected cell viability. Regarding QDs, CdTe—NH_2_ was highly cytotoxic for THP‐1 cells when compared to COOH‐ or PEG‐modified QDs. Similar patterns were observed for Ag NPs and Au NPs (5 and 20 nm), for which only the ammonium modified ENMs triggered cell death. To summarize, the results obtained using HMDMs and THP‐1 cells showed good concordance (**Figure** **1**a,b). This is notable not least as differentiated macrophages are adherent when cultured ex vivo, while undifferentiated monocytes grow in suspension. However, some differences were also observed. Hence, QDs were more prone to induce cell death in HMDMs irrespective of surface modification when compared to THP‐1 cells. Overall, while certain ENMs appear to be inherently more cytotoxic (such as, QDs and CuO NPs), the ENMs with amino/ammonium groups (−NH_2_/−NR^3+^) tended to be more cytotoxic, as seen, for instance, in the case of the Ag and Au NPs (5 and 20 nm). Moreover, PEGylation markedly reduced the cytotoxic potential of some ENMs, such as the CuO NPs. Our studies also confirmed that TiO_2_ NPs (regardless of shape and surface modification) appeared inert, at least in the case of monocytes/macrophages, in line with our previous studies of TiO_2_ NPs using primary human macrophages and dendritic cells.^[^ ^19^ ^]^

### 2.4 Cytokine Profiling

**Figure 2:**
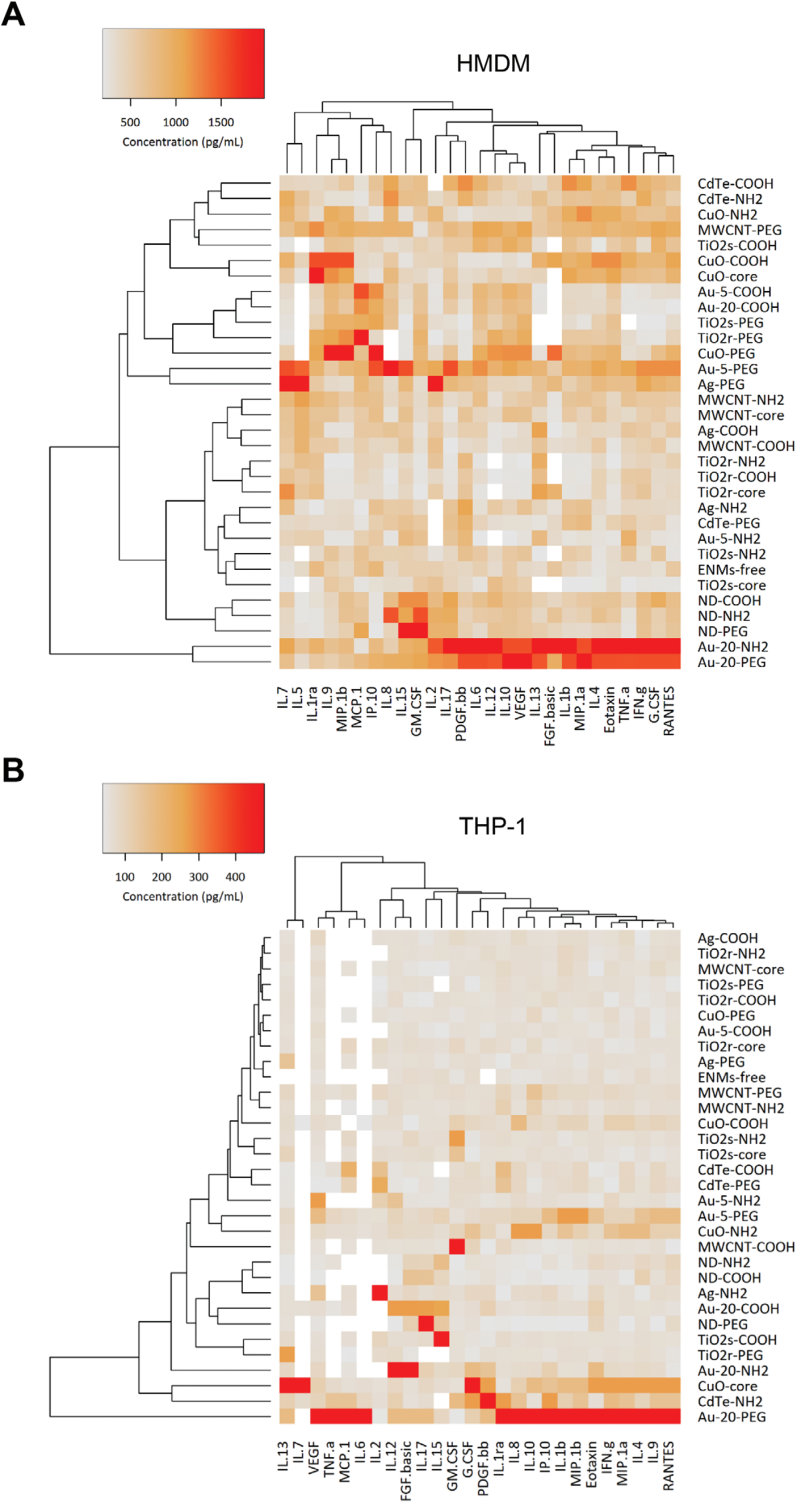
Cytokine profiling of HMDMs A) and THP‐1 cells B) exposed to 31 different ENMs. The cells were exposed to the ENMs for 24 h at doses inducing a maximum of 10–15% of cell death (EC_10_). Cell culture medium alone was used as a negative control. The secretion of cytokines, chemokines, and growth factors into the supernatant was monitored by using the BioPlex Pro Human Cytokine Standard 27‐Plex array (Figure S5, Supporting Information). The heatmaps represent the mean concentrations detected in 3 individual experiments. A comparison of results obtained in HMDMs and THP‐1 cells is shown in Figure S6 (Supporting Information).

To evaluate the immune responses following ENM exposure, we quantified the production of a panel of human cytokines, chemokines, and growth factors by using a multiplex array, as previously described.^[^ ^18^ ^]^ HMDMs and THP‐1 cells were exposed to a dose corresponding to a maximum of 10–15% cell death (EC_10_) at 24 h. Cells incubated in medium alone or with LPS (100 ng mL^−1^) for 24 h were used as negative and positive controls for immune activation, respectively. Overall, the cytokine responses were more pronounced in HMDMs as compared to THP‐1 cells (**Figure** **2**a,b). The results are reported for 23 of the 27 biomarkers in the multiplex array; the remaining cytokines were below the detection limit (Figure S5, Supporting Information). Notably, surface‐modified Au NPs (5 and 20 nm) were found to trigger the production of the Th1‐type cytokines, IFN‐*γ*, and IL‐12, as well as the Th2 cytokine, IL‐10, and several proinflammatory mediators including IL‐6 and TNF‐*α*. Au NPs also elicited the secretion of the chemokines, MIP‐1*α* (CCL3) and RANTES (CCL5), but not MIP‐1*β* (CCL4). We performed hierarchical cluster analysis^[^ ^20^ ^]^ to highlight associations between the different ENMs in relation to their cytokine profiles in the two cell models (Figure S6, Supporting Information). Three clusters were identified: cluster I which groups the most inflammogenic ENMs, such as PEGylated Au NPs (5 and 20 nm); cluster II which groups mainly the THP‐1 responses; and cluster III which groups mainly the cytokine responses in HMDMs. To better visualize the responses in the two cell models, the results for HMDMs are shown in Figure 2a, while results in THP‐1 cells are shown in Figure 2b. Again, noncytotoxic Au‐20‐PEG (Figure 1) stands out as being the most inflammogenic NP.

### 2.5 Proteomics Analysis

**Figure 3:**
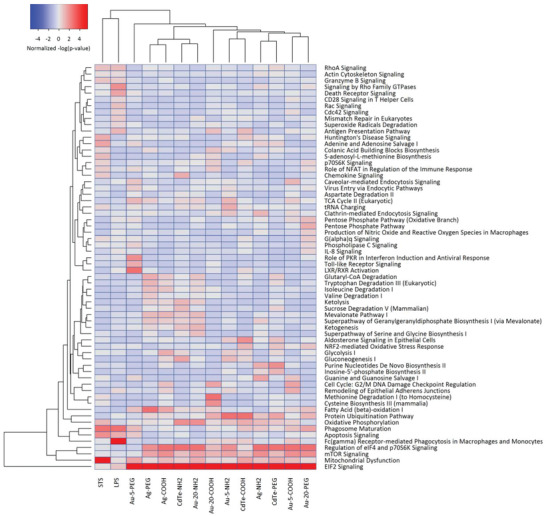
Pathway analysis of proteomics data. THP‐1 cells were exposed to the 31 ENMs for 24 h at doses inducing a maximum of 10–15% of cell death (EC_10_) and samples were then subjected to proteomics analysis (refer to Figure S7, Supporting Information). STS (EC_10_) and LPS (100 ng mL^−1^) were used as positive controls for cell death induction and immune activation, respectively. Pathway analysis was then performed using the Ingenuity Pathway Analysis (IPA) software.^[^ ^22^ ^]^ The heatmap shows the results for the metal ENMs. The corresponding results for metal oxide and carbon‐based ENMs are reported in Figure S8a,b (Supporting Information), respectively. The significance values for the canonical pathways were calculated by Fisher's exact test right‐tailed and indicate the probability of association of the proteins with the respective pathway. The cutoff for p‐values was *p* < 0.001 for at least one of the conditions.

To further probe the biological responses to ENMs, THP‐1 cells were exposed for 24 h at the EC_10_ doses of the 31 ENMs. Staurosporine (0.3 × 10^−6^ m) and LPS (100 ng mL^−1^) were used as positive controls for cell death induction and immune activation, respectively. LC‐MS/MS‐based proteomics analysis was performed as previously described.^[^ ^21^ ^]^ The heatmap in Figure S7 (Supporting Information) shows the unsupervised clustering of the top‐100 proteins identified for all ENMs. No distinct clusters could be identified suggesting a nonspecific response to ENMs. Nevertheless, in order to extract as much information as possible, pathway analysis was performed using the Ingenuity Pathway Analysis (IPA) software tool.^[^ ^22^ ^]^ **Figure** **3** shows the analysis of the different metal ENMs with activation (red) or inactivation (blue) of the most significant canonical pathways (*p* < 0.001). Notably, the pathway, “EIF2 signaling,” was found to be predominantly upregulated by all the ENMs. EIF2 (eukaryotic translation initiation factor 2) is an essential factor for protein synthesis.^[^ ^23^ ^]^ As expected, staurosporine upregulated the “mitochondrial dysfunction” pathway, while LPS triggered the pathway related to phagocytosis in macrophages and monocytes (Figure 3). The corresponding heatmaps for the metal oxide and carbon‐based ENMs are shown in Figure S8a,b (Supporting Information), respectively. Overall, the proteomics data indicated a general stress response in cells exposed to ENMs at EC_10_ doses.

### 2.6 Transcriptomics Analysis

**Figure 4:**
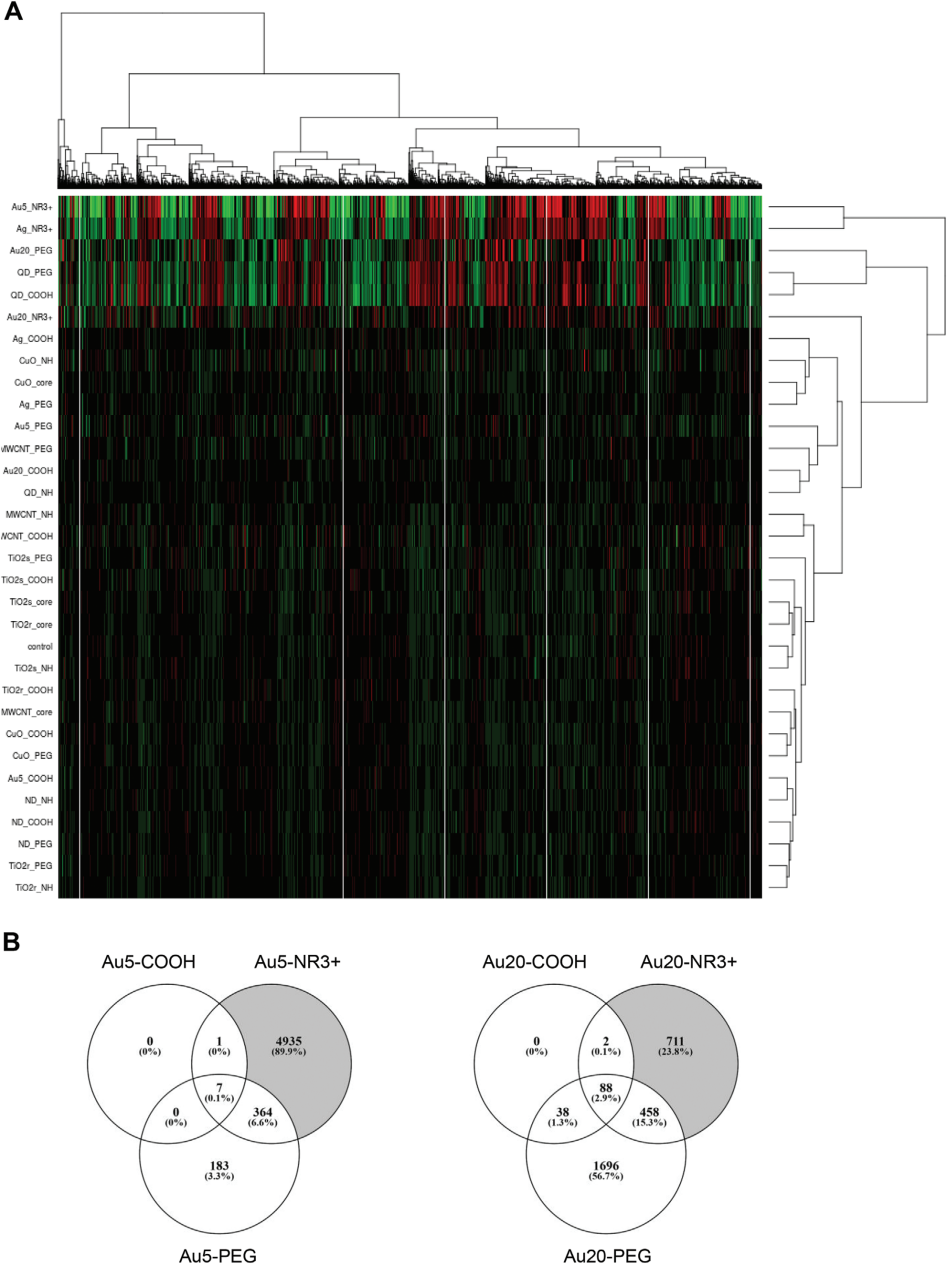
Unsupervised clustering of microarray data. A) Hierarchical clustering of differentially expressed genes (DEGs) significantly regulated in THP‐1 cells after 24 h exposure to the 31 ENMs at doses inducing a maximum of 10–15% of cell death (EC_10_). The heatmap was colored based on activation z scores of the normalized expression levels for the DEGs; refer to Figure S9 (Supporting Information) for the hierarchical clustering results prior to averaging of the replicates. B) Venn analysis of significant DEGs (FC > 1.5 and adjusted *p*‐value < 0.05) in THP‐1 cells exposed to different Au (5 nm) ENMs and Au (20 nm) ENMs.

We then performed genome‐wide expression analysis of THP‐1 cells. To this end, THP‐1 cells were exposed for 24 h at the EC_10_ doses of the 31 ENMs, and total RNA was harvested for microarray analysis, as described previously.^[^ ^24^ ^]^ **Figure** **4**a depicts the hierarchical clustering of the differentially expressed genes (DEGs) found to be significantly regulated by ENMs in THP‐1 cells (average gene expression values derived from three replicates; the cluster analysis before averaging of the replicates is shown in Figure S9, Supporting Information). Notably, the strongly cytotoxic Au‐5‐NR^3+^ and Ag‐NR^3+^ ENMs clustered together on the basis of the deregulated DEGs. A second, closely related cluster was formed by the noncytotoxic ENMs, Au‐20‐PEG, QD–PEG, and QD–COOH, while Au‐20‐NR^3+^ (also cytotoxic) was also closely clustered yet formed a separate node in the dendrogram (Figure 4a). However, other cytotoxic ENMs such as CuO‐core or CuO–NH_2_ did not cluster with the ammonium‐modified Au or Ag ENMs. Overall, these results serve to illustrate that cytotoxicity profiling of ENMs does not necessarily align with the gene expression profiles of the ENMs (following a low‐dose exposure). Indeed, the Au‐5‐NR^3+^, Au‐20‐NR^3+^, and Ag‐NR^3+^ ENMs were all strongly cytotoxic, while the Au‐20‐PEG, QD–PEG, and QD–COOH ENMs were noncytotoxic (as shown in Figure 1b; and Figure S4, Supporting Information), yet these six ENMs all displayed closely related gene expression changes (Figure 4a).

Venn diagrams highlighted that the ammonium modified Au NPs (5 and 20 nm) triggered the most pronounced responses with the greatest number of unique DEGs (Figure 4b). Thus, at least for Au NPs, the ammonium modification results in stronger biological responses overall, both with respect to cell death and in terms of gene expression changes. However, the PEGylated Au NPs evidently triggered significant changes in gene expression yet displayed no overt cytotoxicity. PEGylated CuO, on the other hand, clustered close to the negative control (cell culture medium alone) in the heatmap (Figure 4a).

**Figure 5:**
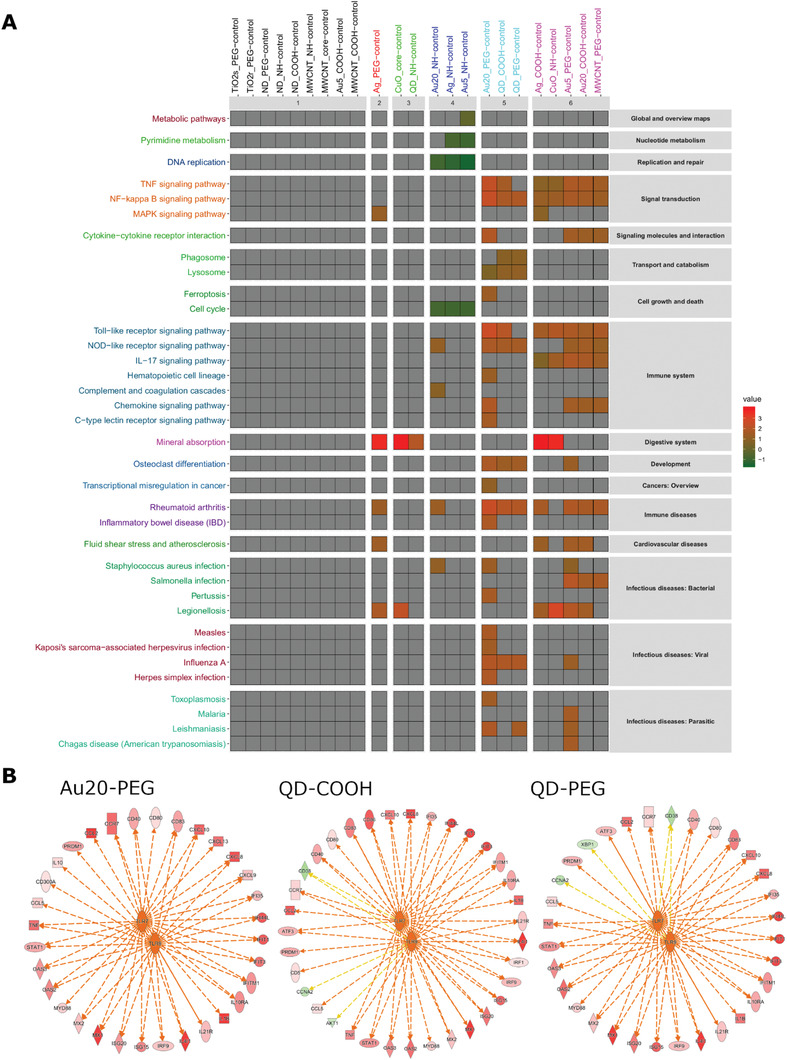
Functional analysis of the transcriptomics data. A) Functional annotation of the microarray data obtained for 23 out of 31 ENMs (i.e., ENMs for which significant DEGs were identified) was performed using the KEGG database^[^ ^29^ ^]^ focusing on “human disease” categories. The results were visualized by using the FunMappOne graphical tool.^[^ ^63^ ^]^ The activation z scores are shown (red: activation, green: deactivation). The cutoff for *p*‐values was *p* < 0.001. B) Upstream regulator analysis of the transcriptomics results was performed,^[^ ^64^, ^65^ ^]^ and TLR7/TLR9 were identified as putative regulators on the basis of z scores. Refer to Figure S10 (Supporting Information) for upstream regulator results with a negative activation z score and the legend for the upstream regulator analysis shown in panel B.

The results presented here represent the largest nano‐transcriptomics dataset to date, providing a wealth of information for further analysis of the biological responses to ENMs. To explore the relevance of the observed gene expression changes, we focused our attention on the six metal ENMs that displayed the most pronounced effects (see above) and performed gene ontology (GO) enrichment analyses.^[^ ^25^ ^]^ The top‐10 biological process GO terms for Ag‐NR^3+^, Au‐5‐NR^3+^, Au‐20‐NR^3+^, Au‐20‐PEG, QD–PEG, and QD–COOH were sorted by adjusted *p*‐value (Table S2, Supporting Information). GO terms were displayed according to up‐ or down‐regulated DEGs. For Ag‐NR^3+^, Au‐5‐NR^3+^, and Au‐20‐NR^3+^, the most significantly downregulated GO terms corresponded to DNA replication, DNA repair, and cell cycle transition. With regard to the upregulated GO terms, all six ENMs showed a significant impact on cytokine‐mediated signaling and inflammation/neutrophil‐mediated immunity. For comparison, CuO‐core and CuO—NH_2_ were found to affect metal detoxification and ion homeostasis (data not shown), suggesting a very different cellular response. We also determined the top‐20 up‐ and downregulated genes for the six metal ENMs (Table S3, Supporting Information). These results do not necessarily match the GO enrichment analysis, as the latter was performed on the full set of significantly up‐ or downregulated DEGs. Several metallothionein‐encoding genes were found to be upregulated in Ag‐NR^3+^ exposed cells. For five out of the six ENMs (Au‐5‐NH^3+^, Ag‐NH^3+^ Au‐20‐PEG, QD–PEG, QD–COOH), SERPINB2 was found to be one of the most strongly downregulated genes (Table S3, Supporting Information). SERPINB2 encodes a serine protease inhibitor abundantly expressed in macrophages and implicated in the regulation of innate and adaptive immune responses.^[^ ^26^, ^27^ ^]^ Moreover, AHRR encoding the aryl hydrocarbon receptor repressor^[^ ^28^ ^]^ was found to be one of the most downregulated genes in cells exposed to surface‐modified MWCNTs (data not shown). To explore the transcriptomics data further, we utilized the KEGG (Kyoto Encyclopedia of Genes and Genomes) database that integrates genomic, chemical, and functional information.^[^ ^29^ ^]^ **Figure** **5**a shows the results for the “human diseases” category for the 23 ENMs (out of 31) for which significant DEGs were identified. Results are plotted based on activation scores (*p* < 0.001). Interestingly, the three transcriptionally “active,” yet noncytotoxic ENMs (i.e., Au‐20‐PEG, QD–COOH, and QD–PEG) clustered together, with activation of NF‐*κ*B, Toll‐like receptor (TLR) and NOD‐like receptor (NLR) signaling. Furthermore, these ENMs all showed activation of infectious disease pathways including the “influenza virus” infection pathway. The three transcriptionally “active” and cytotoxic ENMs (i.e., Ag‐NR^3+^, Au‐5‐NR^3+^, Au‐20‐NR^3+^) did not show any overlap with Au‐20‐PEG, QD–COOH, and QD–PEG. Instead, these ENMs were characterized by inactivation of DNA replication and cell cycle pathways (Figure 5a), in line with the GO enrichment analysis (Table S2, Supporting Information). Moreover, upstream regulator analysis using IPA identified TLR7 and TLR9 as potential regulators of gene expression in cells exposed to Au‐20‐PEG, QD–COOH, and QD–PEG (Figure 5b). Furthermore, Bruton tyrosine kinase (BTK) was identified as a potential upstream regulator with a negative activation score (Figure S10b, Supporting Information). Venn analysis showed a considerable overlap between QD–COOH and QD–PEG (Figure S10a, Supporting Information).

### 2.7 Validation Experiments

**Figure 6:**
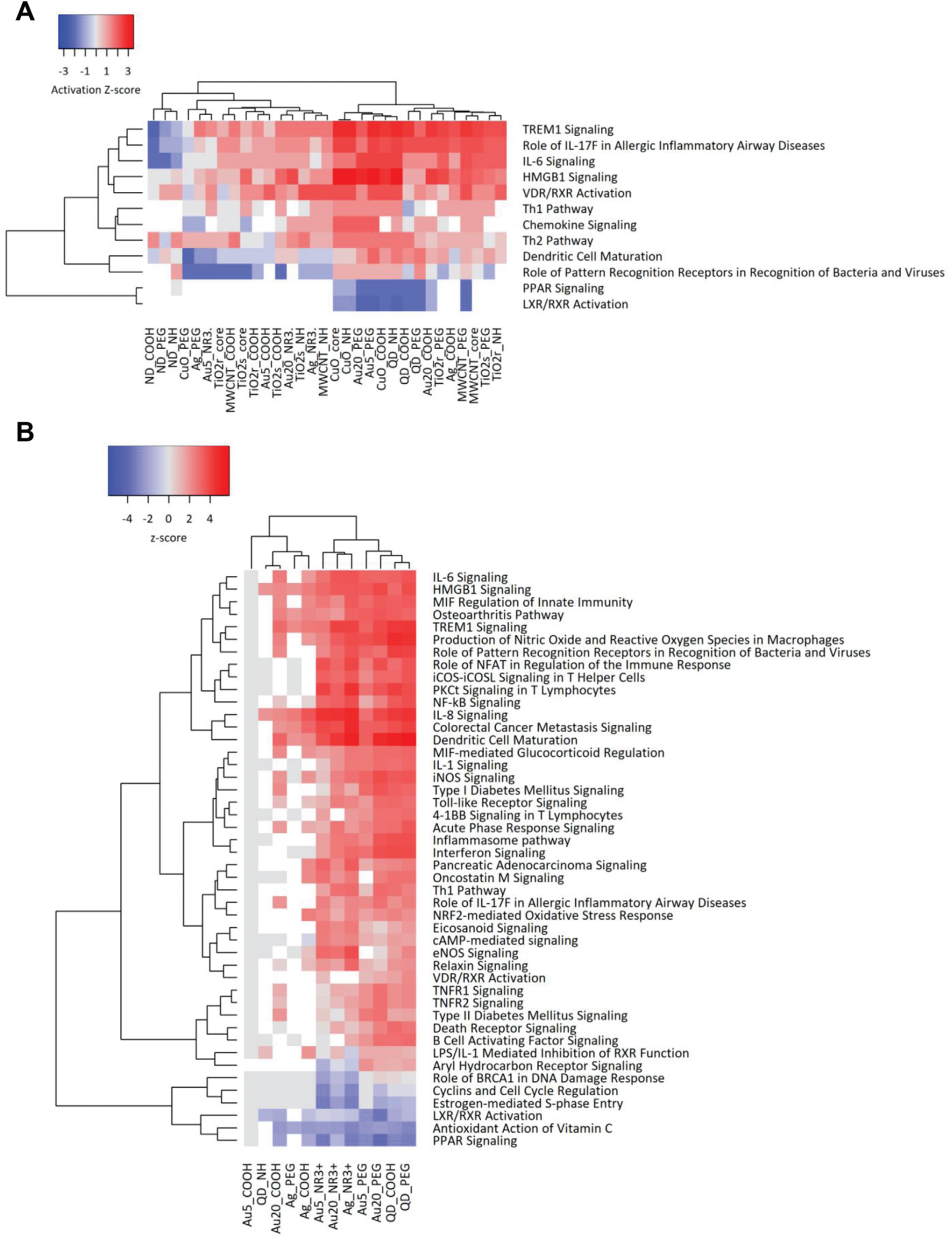
Pathway analysis of immune responses. A) Pathway analysis was performed by using IPA software,^[^ ^22^ ^]^ by integrating the normalized cytokine profiling results of THP‐1 cells exposed to the 31 ENMs. The results shown highlight the grouping of ENMs into activators and deactivators of nuclear receptor (PPAR and LXR/RXR) pathways, respectively. The heatmaps show the significant canonical pathways with activation score > 2, and adjusted *p*‐value < 0.05 for at least one activated pathway per treatment. B) Pathway analysis was also performed on transcriptomics results obtained for the metal ENMs. The heatmap based on activation z scores depicts the activation (red) or inactivation (blue) of the most significant canonical pathways associated with the various conditions. The significance values for the canonical pathways were calculated by Fisher's exact test right‐tailed and indicates the probability of association of DEGs with the respective pathway. The cutoff for the *p*‐value was *p* < 0.001 for at least one of the conditions.

Since our transcriptomics analysis pointed to the upregulation of cytokine‐mediated signaling for the most transcriptionally “active” metal ENMs, we decided to explore cytokine responses further by using the IPA software tool. We previously provided evidence, in an unrelated study of 19 ENMs, that grouping could be achieved on the basis of cytokine responses in THP‐1 cells. Hence, by using multiplex arrays coupled with bioinformatics analysis of the cytokine results, we found that the ENMs could be segregated into two distinct groups based on the activation or deactivation of the nuclear receptors, PPAR (peroxisome proliferator‐activated receptor) and LXR/RXR (liver X receptor/retinoid X receptor).^[^ ^18^ ^]^ We decided to test this proposal in the present study. To this end, canonical pathway analysis was performed on the cytokine data obtained for the full set of ENMs (Figure 2b). As shown in **Figure** **6**a, the ENMs segregated into two groups, with activation or deactivation of PPAR and LXR/RXR. Furthermore, canonical pathway analysis was performed on the transcriptomics data obtained for the metal ENMs. Interestingly, as shown in Figure 6b, the ENMs were found to inactivate PPAR and LXR/RXR along with DNA damage and cell cycle related pathways (*p* < 0.001), suggesting that: i) nuclear receptor activation/deactivation in monocyte‐like THP‐1 cells is a valid paradigm for ENMs, and ii) cytokine profiling and transcriptomics results for the studied metal ENMs show good concordance at the level of pathway activation.

**Figure 7:**
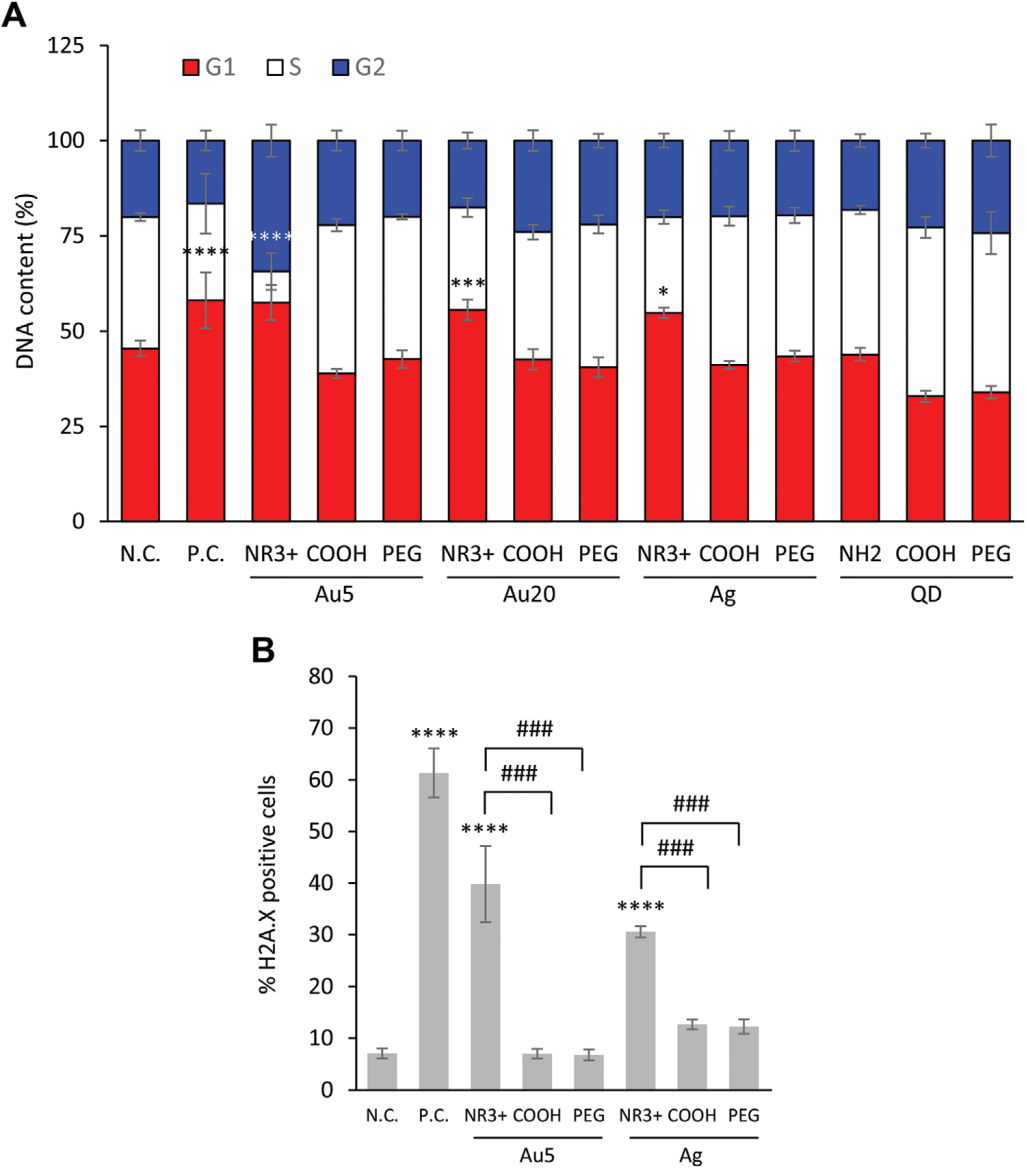
Validation of microarray results. A) Quantification of cell cycle analysis results for THP‐1 cells exposed for 24 h at the EC_10_ dose of the indicated metal ENMs, i.e., Ag, Au (5 nm), and CdTe. N.C., negative control (medium alone); P.C., positive control (etoposide, 10 × 10^−6^ m). Data analyzed by one‐way ANOVA followed by Dunnett test: *p < 0.05, ***p < 0.001, ****p < 0.0001. B) DNA damage was determined by staining cells with an anti‐histone H2A.X (Ser139) antibody with quantification by flow cytometry. Cells were exposed as described in (A). N.C., negative control; P.C., positive control (etoposide). Data analyzed by one‐way ANOVA followed by Dunnett's test for comparison to N.C. or by Bonferroni's test for multiple comparisons: *p < 0.05, ***p < 0.001, ****p < 0.0001, ^###^ *p* < 0.001.

To further validate the microarray results, we turned our attention to the most downregulated biological processes, i.e., DNA replication/repair, and cell cycle transition (Table S2, Supporting Information). First, we applied the conventional DNA content/cell cycle assay,^[^ ^9^ ^]^ using THP‐1 cells exposed for 24 h at the EC_10_ dose. Etoposide (10 × 10^−6^ m) was included as a positive control. **Figure** **7**a shows the results for all the metal ENMs tested (i.e., Ag, Au 5 nm, Au 20 nm, and CdTe, with three different surface modifications). Notably, the ammonium modified Ag and Au (5 and 20 nm) ENMs all triggered a strong cell cycle arrest comparable to that seen for etoposide while this was not the case for their carboxyl modified or PEGylated counterparts. Next, we evaluated DNA damage in cells by using a specific phospho‐histone H2A.X antibody. Etoposide (10 × 10^−6^ m) was used as a positive control. Both Au‐5‐NR^3+^ and Ag‐NR^3+^, the two ENMs that were shown to be the most closely related according to hierarchical clustering of DEGs (Figure 4a), triggered a significant degree of DNA damage (Figure 7b) comparable to that seen for etoposide treated cells. The carboxyl modified or PEGylated ENMs did not elicit DNA damage. Thus, the transcriptomics results were validated with respect to DNA damage and cell cycle arrest.

Previous work has shown that NPs with a high affinity for DNA strongly inhibited DNA replication, whereas those with low affinity had no or minimal effects.^[^ ^30^ ^]^ We therefore decided to study the interaction of Au NPs of different diameters and varying surface modifications with respect to their binding to RNA and DNA. To this end, we isolated total cellular RNA and DNA from THP‐1 cells using standard protocols. Then, ENMs were incubated with 25 ng of RNA or 50 ng of DNA for 15 min (on ice). To monitor binding, we performed DLS and zeta potential measurements (data not shown) and PCR (Figure S11a,b, Supporting Information). These studies showed, not unexpectedly, that the positively charged ammonium modified ENMs interacted more readily with RNA (Figure S11a,b, Supporting Information) and DNA (Figure S11c, Supporting Information). These experiments were performed with “naked” DNA, while in the cell, DNA is bound to histones, forming nucleosomes. Nevertheless, the results suggest that direct nucleic acid binding could potentially contribute to the effects observed for ammonium modified Au NPs.

## 3 Discussion

In this study, we performed a multiparametric evaluation of a large panel of ENMs using human in vitro models representing the immune system.^[^ ^31^ ^]^ Our overarching conclusion is that the surface properties are a key determinant of the biological responses toward EMNs, thus supporting our initial hypothesis. However, core chemistry also plays a role, and this was evidenced both when studying cytotoxicity and gene expression changes. Furthermore, ENMs that do not display cytotoxicity may still elicit major effects on cells. Indeed, certain PEGylated metal NPs were found to be highly inflammogenic, and the transcriptional responses were reminiscent of those induced by viruses, as we discuss below.

Previous toxicity profiling studies have focused mainly on a single class of ENMs. For instance, Cai et al.^[^ ^32^ ^]^ studied a panel of Fe_2_O_3_ ENMs by applying metabolomics and proteomics analyses (at relatively high doses, i.e., 100 µg mL^−1^). Shaw et al.^[^ ^7^ ^]^ performed profiling studies of iron oxide ENMs (Fe_2_O_3_ and Fe_3_O_4_) plus QDs (CdSe) using conventional assays. Zhang et al.^[^ ^33^ ^]^ explored the biological effects of a panel of metal oxides and Ag ENMs. Breznan et al.^[^ ^34^ ^]^ focused on a panel of mesoporous silica particles, while Wu et al.^[^ ^35^ ^]^ studied a large set of surface modified MWCNTs. Taken together, these studies have provided valuable insights with respect to nano–bio interactions insofar as they showed that different physicochemical properties, such as surface reactivity, or size, or shape/aspect ratio, or a combination of several properties, may dictate cellular outcomes. Cell type‐specific differences were also noted in several studies. What does the present study add to this picture? First, using a broad panel of ENMs encompassing metal, metal oxide, and carbon‐based ENMs, we showed that surface properties are an important driver of the cytotoxicity of ENMs. This was verified in a commonly used human cell line as well as in primary human macrophages. However, the relation between physicochemical properties and biological responses is complex and the core chemistry also matters. Interestingly, we found that while certain surface modifications, i.e., amino/ammonium termination, may convert ENMs that are inherently inert (such as Au) into a strongly cytotoxic material, the same surface modification had no effect on other ENMs (such as TiO_2_ or NDs) that remained biologically inert no matter the surface properties (in the current model). For comparison, Orecchioni et al. studied the impact of pristine graphene oxide (GO) versus GO functionalized with amino groups (GO–NH_2_) on various subpopulations of peripheral blood mononuclear cells by using single‐cell mass cytometry and supplemented this with genome‐wide transcriptomics analysis of GO and GO–NH_2_ exposed Jurkat cells and THP‐1 cells.^[^ ^36^ ^]^ Using pathway analysis, the authors found that the perturbations induced by GO were reflective of cytotoxic mechanisms, while the transcriptional changes induced by GO–NH_2_ were consistent with immune activation.^[^ ^36^ ^]^ Thus, the impact of amino/ammonium functionalization may potentially depend on how the functional groups are displayed (i.e., on a flat surface as opposed to a surface with high curvature). We noted that while PEGylation of ENMs, a commonly used strategy in nanomedicine,^[^ ^37^ ^]^ may passivate certain ENMs (such as CuO) the same surface modification may promote the inflammogenic properties of other ENMs (such as Au) and may even enhance toxicity of certain ENMs (as seen for MWCNTs). Hence, this further supports the view that the manner in which the functional groups are presented also matters. The stability or lability of surface ligands may also be a factor, as suggested in a previous microarray study of surface modified Au NPs.^[^ ^38^ ^]^ Overall, carboxyl functionalization appeared to be the most biocompatible modification in the present study. However, it is important to qualify this statement in at least two ways. First, macromolecules (such as PEG) may acquire different conformations depending on a number of factors including the type of bond anchoring the ligand to the surface, the curvature of the particle, and the ionic strength and pH of the surrounding media.^[^ ^39^ ^]^ Second, differences in surface coating may lead to the adsorption of different proteins or other biomolecules which, in turn, may impact on cellular uptake of ENMs.^[^ ^40^, ^41^ ^]^ Notwithstanding, our survey of a large panel of ENMs using two cell models has shown that surface functionalization (coating) is an important determinant of the cellular effects of ENMs.

The present study takes as its starting point the administered dose (the amount added to the cell cultures) as opposed to the internalized dose, although the concentration of ENMs internalized by cells may, in principle, vary depending upon the surface chemistry (and, potentially, the composition of the so‐called corona).^[^ ^42^ ^]^ However, we have studied the internalization of spherical TiO_2_ ENMs (pristine and surface‐modified) by using TEM and found that these ENMs were avidly taken up by HMDMs regardless of surface functionalization (unpublished observations). On the other hand, as shown in the present study, none of these ENMs displayed any cytotoxicity toward macrophages (or THP‐1 cells). These results thus dispel the notion that cellular uptake equals toxicity, at least for TiO_2_ ENMs. Furthermore, it is of interest to note that the cytotoxicity results for the entire panel of ENMs were largely concordant between the two cell models, despite the fact that primary macrophages are more proficient in terms of phagocytosis when compared to cell lines.

The second major point of interest is that we have shown, using a low‐dose (equipotent) exposure regimen (to avoid excessive cell death as this may confound the results), that transcriptional responses to ENMs are related to surface properties. The current dataset spanning 31 different ENMs is the largest transcriptomics dataset thus far, and our results provide a useful resource for in silico exploration of nano–bio interactions, as exemplified in previous publications.^[^ ^43^, ^44^ ^]^ The most striking finding in this regard was that the small, ammonium modified Ag and Au ENMs which were found to be strongly cytotoxic in our model were also clustered together based on significant changes in gene expression (at EC_10_). Furthermore, with regard to the six different metal (Ag, Au, CdTe) ENMs that were found to be the most transcriptionally “active” among the 31 ENMs, we found a remarkably consistent pattern in terms of affected cellular pathways. Hence, the downregulated pathways were related to DNA replication/DNA repair and cell cycle transition, while the upregulated pathways were associated with cytokine‐dependent signaling and inflammation. Importantly, these findings were validated by using conventional cell‐based assays. It is noteworthy that not all cytotoxic ENMs displayed the same pattern of gene expression changes. For instance, cytotoxic CuO ENMs were found to activate metal detoxification pathways, likely due to the release of toxic ions, as shown in several previous studies.^[^ ^45^, ^46^ ^]^ On the other hand, our proteomics analysis did not distinguish specific effects for any of the ENMs and pointed to a general activation of EIF2 signaling (i.e., protein translation). This shows that gene expression profiling is a more sensitive method able to discern low‐dose effects of ENMs. However, we reported in a previous study on a set of Au ENMs that proteomics can be applied to detect changes at doses corresponding to EC_50_ and found a good concordance between transcriptomics and proteomics approaches in THP‐1 cells.^[^ ^12^ ^]^In line with the latter findings, a recent proteomics study of metal oxide ENMs (SiO_2_ and TiO_2_) and GO showed no effects in a lung epithelial cell line at low doses (≤ 1 µg cm^−2^) while doses of 10 µg cm^−2^ yielded significant changes.^[^ ^47^ ^]^

It is interesting to note that three ENMs (Au‐20‐PEG, QD–COOH, and QD–PEG) were noncytotoxic as shown by conventional toxicity assays, yet they were found to elicit significant changes in gene expression in THP‐1 cells. Our bioinformatics analyses revealed that these ENMs activated NF‐*κ*B, TLR, and NLR signaling pathways. Furthermore, the gene expression profiles matched those of several infectious diseases including influenza virus infection, and upstream regulator analysis predicted both TLR7 and TLR9 as positive regulators and BTK as a negative regulator of gene expression. TLR7 and TLR9 are endosomal pattern recognition receptors that function as sensors of single‐stranded RNA and double‐stranded DNA, respectively.^[^ ^48^ ^]^ BTK, in turn, is involved in the sensing of multiple microbes, in part, via TLRs.^[^ ^49^ ^]^ Taken together, these observations imply that Au‐20‐PEG, QD–COOH, and QD–PEG are “sensed” as microbes. This may not be surprising if one considers that viruses are nanosized particles (e.g., influenza virus particles are between 80 and 120 nm) that display repetitive molecular motifs on their surface; these molecular patterns are, in turn, recognized by extracellular or intracellular pattern recognition receptors, such as TLRs.^[^ ^50^ ^]^ Moreover, viruses were recently shown to display a “corona” of surface adsorbed proteins,^[^ ^51^ ^]^ further blurring the distinction between synthetic and natural nanoparticles. Previous studies have provided evidence that QD–COOH and QD–PEG activate TLR signaling pathways in murine RAW264.7 macrophages,^[^ ^52^, ^53^ ^]^ though this does not prove a direct interaction of QDs with TLRs. Moreover, Au NPs were shown to modulate TLR9 signaling within macrophage lysosomes in a size‐dependent manner.^[^ ^54^ ^]^ Luo et al. recently demonstrated that PEGylated GO stimulates cytokine responses in peritoneal macrophages, and comparable results were obtained for the unrelated 2D material, MoS_2_.^[^ ^55^ ^]^ Therefore, the widely held assumption that PEGylation serves to passivate ENMs may not be correct. Instead, it appears that PEGylation promotes inflammogenic responses, at least for certain ENMs (such as Au). QDs (with or without PEG) may be especially prone to trigger “virus‐like” responses by virtue of their small size and spherical shape.

In sum, we report multiparametric assessment of a large panel of ENMs and show that surface properties are key determinants of cellular (immune) responses to ENMs. However, the core chemistry (composition) of the ENMs is also important. Our findings also demonstrate that ENMs can be discriminated on the basis of transcriptional profiling.

## 4 Experimental Section

##### ENM Synthesis and Functionalization

Spherical TiO_2_ ENMs (TiO_2_s‐core) were prepared at room temperature by hydrolysis of titanium tetrachloride solution with a further condensation of the reaction products, and stabilization by nitric acid. TiO_2_ nanorods (TiO_2_r‐core) were obtained by forced hydrolysis in acidic conditions at normal pressure, leading to high yield and desired diameter and aspect ratio. TiO_2_‐spheres and TiO_2_‐rods were surface modified using a multistep procedure. First, the particle cores were modified with an NH_2_‐terminated silane (APTMS), yielding to amino‐modified TiO_2_. Then, to produce COOH and PEG modified particles, succinic anhydride and PEG‐COOH were bound to amino group through amide bonds, respectively. For the synthesis of the two Au ENMs (Au‐5 and Au‐20) the strategy consisted in growing the metallic NPs with the simultaneous attachment of self‐assembled thiol monolayers on the growing nuclei in order to allow the surface reaction to take place during metal nucleation and growth. Briefly, Au‐5 were prepared using reduction of Au^3+^ to Au^0^ with NaBH_4_ in the presence of bifunctional ligands of the type XRSH (X = COOH, N(CH_3_)_3_, or CH_3_) so that the surface is terminated with these functionalities. The ligands bind to the gold surface through their thiolate (RS end). Au‐5‐PEG were obtained by direct synthesis with controlled ratio of [HAuCl_4_]/[poly(ethylene glycol) methyl ether thiol average 550 g mol^−1^]. The Au‐20 were synthesized by reduction of Au^3+^ to Au^0^ with Na_3_ citrate which yielded Au ENMs of core size > 10 nm. The citrate reduction process involves hot gold chloride and sodium citrate as reactants. In this reaction, the citrate molecules act as both reducing and stabilizing agents, allowing for the formation of the colloidal gold. The bifunctional ligands of the type XRSH (X = COOH, N(CH_3_)_3_, and CH_3_) were used in order to replace the citrate ligands on the nanoparticle surface. Poly(ethylene glycol) methyl ether thiol (average 550 g mol^−1^) was the bifunctional ligand used in the synthesis of the Au‐20‐PEG. CuO ENMs were synthesized by the precipitate decomposition method. A precursor, basic copper carbonate (Cu_2_(OH)_2_CO_3_), was first prepared by precipitation reaction from an aqueous solution composed of 1 m copper nitrate and sodium carbonate. The obtained precursor was dewatered, dried, and milled. Nanocrystalline CuO particles were obtained by thermal decomposition of the precursor at 300 °C for 2 h. For surface modification of the CuO ENMs, the thiol‐ligands, HSHCH_2_CH_2_NH_2_, HSCH_2_COOH, and HSCH_2_COOPEGOCH_3_, were used due to their strong ability to bind to copper oxide in order to obtain CuO—NH_2_, CuO—COOH, and CuO–PEG ENMs, respectively. Ag ENMs were prepared by reduction of Ag^+^ to Ag^0^ with excess ice‐cold NaBH_4_ solution. The bifunctional ligands of the type XRSH (X = COOH, N(CH_3_)_3_, and CH_3_) were added and also provided an efficient stabilization. Ag‐PEG was obtained by using the [poly (ethylene glycol) methyl ether thiol, average 550 g mol^−1^] ligand. CdTe QDs were synthesized by PlasmaChem GmbH (Berlin) in the frame of FP7‐NANOSOLUTIONS. Briefly, cadmium and tellurium precursors were mixed together with a thiocarboxylic acid, which serves as a stabilizing ligand. After introduction of the reducing atmosphere, the solution was refluxed until a desirable fluorescence emission wavelength is achieved. The carboxylated QDs (CdTe—COOH) had an emission wavelength of 590 nm. Modification of CdTe—COOH with amino and PEG groups was performed through the ligand exchange procedure with the respective thiols. Briefly, CdTe—COOH QDs were extensively washed with a solvent mixture containing an excess of a target ligand (HS‐ *_n_* R‐(PEG) or HS‐R‐NH_2_) followed by incubation of the obtained QDs in the suspension of a pure target ligand. The process of ligand exchange was monitored by the dispersibility of the QDs in water and change of the optical properties of QDs. NDs were prepared by detonation in two stages: first, synthesis of nanodiamond soot by explosion of mixed trinitrotoluene‐hexogen explosive charges (TNT–RDX 60/40) in a large‐sized chamber (50 m^3^) at a raised pressure of a cooling gas, and second, oxidation with a HNO_3_—HClO_4_ mixture of metals and nondiamond carbon and isolation of the carboxylated NDs (ND—COOH). The carboxylic groups were used as reactive sites for the ligands —C(O)—NH—(CH_2_)_2_NH—(CH_2_)_2_NH—(CH_2_)_2_—NH_3_Cl and —C(O)O—CH_2_CH_2_—(O—CH_2_—CH_2_)11—O—CH_3_ in order to obtain ND—NH_2_ and ND–PEG. MWCNT‐core, MWCNT–NH_2_ and MWCNT–COOH were produced by Nanocyl SA (Sambreville) in the frame of FP7‐NANOSOLUTIONS via catalytic carbon vapor deposition (CCVD) (NC7000, NC3152, and NC3151, respectively). For PEGylated MWCNTs, 1,2‐distearoyl‐sn‐glycero‐3‐phosphoethanolamine‐*N*‐[methoxy(poly ethylene glycol)‐2000] (DSPE‐PEG 2000) was used for a noncovalent functionalization process. 125 mg of DSPE‐PEG 2000 was first dissolved in 20 mL of water, then added to the pristine MWCNT (25 mg) at a 1:5 [MWCNT:DSPE‐PEG 2000] weight ratio. The mixture was then sonicated at room temperature for 1 h and diluted to 250 mL with water before filtration through a Millipore 100 kDa cut‐off filter 5 times to remove any unbound DSPE‐PEG 2000. The final product of MWCNT–PEG was then resuspended in water to give the final product of MWCNT–PEG.

##### ENM Characterization

DLS and zeta potential measurements were performed on freshly prepared ENM suspensions using a Malvern Zetasizer Nano ZS (model ZEN3600) operating with a 632.8 nm laser wavelength. The angle of detection was set at 173° and the temperature was maintained at 25 °C throughout the analysis. The Zetasizer v.6.32 software was used for data processing. UV–vis measurements were performed on a UV–vis spectrometer Lambda 750 (Perkin Elmer). The parameters for refractive index were set according to the ENM core type measured, the range of acquisition covered 200 to 800 nm and the interval was set at 0.5 or 1 nm. The software UVWinLab was used for data processing. Photoluminescence analysis of QDs was done on a LS‐55B Luminescence spectrometer (Perkin Elmer). The excitation wavelength was set at 550 nm and emission was collected from 560 to 700 nm. The software FLWinLab was used for data processing. Raman spectroscopy of MWCNTs was performed using a WITec alpha 300 RAS instrument, coupled to a 532 nm wavelength laser operating with the WITec project FOUR software. One single acquisition represents the average of 10 spectra each integrated over 0.5 s. The presence of impurities (Al, Si, Mn, Fe, Co, Ni, Cu, Cr, Ga, Fe, Br, Sr, As, Mo, Ag, Sn, Sb, Te, W, Au, Pb) was investigated by inductively coupled plasma‐mass spectrometry. The presence of functional groups was confirmed with XPS, FTIR, and thermogravimetric analysis (TGA). Complete datasets can be retrieved from the NANOSOLUTIONS repository (refer to Table S1 for a summary, Supporting Information).

##### Endotoxin Assessment

The procedure for the detection of gram‐negative bacterial endotoxin followed the QCL‐1000 Endpoint Chromogenic LAL assay (Lonza) protocol. The enzymatic reaction was performed on ENMs alone, ENMs in presence of LPS or ENMs in presence of LPS and polymyxin B (PolyB). Briefly, the stock suspensions of ENMs were diluted at 100 µg mL^−1^ in endotoxin‐free culture medium and 50 µL of each suspension were dispensed into a 96‐well plate. LPS and PolyB were added at 1–3 EU mL^−1^ or 5–10 × 10^−6^ m, respectively, following by 1 h incubation at room temperature. The reaction was initiated by first adding the proenzyme and then the substrate. Then, a stop solution of acetic acid 25% v/v in dH2O was added and the absorbance was determined at 405 nm, using an Infinite 200 Tecan microplate reader operating with Magellan v7.2 software. For ENMs for which assay interference was shown, the macrophage‐based TET assay was deployed.^[^ ^17^ ^]^ Briefly, HMDMs were obtained from human monocytes seeded into a 96‐well plate and differentiated with recombinant human M‐CSF (PeproTech) for 3 days. ENMs suspensions were diluted to 5 µg mL^−1^ in cell culture medium, supplemented or not with PolyB (10 × 10^−6^ m). HMDMs were then exposed for 24 h to the ENM suspensions, or culture medium alone, or LPS at 100 ng mL^−1^ (positive control for TNF‐*α* production). Following incubation, cell culture supernatants were collected and the levels of TNF‐*α* were monitored using a specific ELISA (Mabtech) according to the manufacturer's protocol.

##### Cell Line and Primary Cells

HMDMs are obtained from peripheral blood mononuclear cells as described.^[^ ^56^ ^]^ Briefly, cells were isolated from buffy coats of healthy adult donors by density gradient centrifugation followed by positive selection of CD14+ monocytes using CD14 MACS magnetic beads (Miltenyi Biotech). Cells were then cultured in RPMI‐1640 medium supplemented with 10% heat‐inactivated bovine serum (FBS), 2 × 10^−3^ m glutamine, 100 U mL^−1^ penicillin, and 100 µg mL^−1^ streptomycin and M‐CSF (50 ng mL^−1^) was added for 72 h to induce macrophage differentiation. The human monocytic THP‐1 cell line was purchased from ATCC (UK). The cells were cultured in RPMI‐1640 medium supplemented with 10% FBS, 2 × 10^−3^ m glutamine, 100 U mL^−1^ penicillin, 100 µg mL^−1^ streptomycin, and 0.05 × 10^−3^ m *β*‐mercaptoethanol. The cell density was strictly maintained at 0.1–2.0 × 10^6^ cells mL^−1^.

##### Cytotoxicity Assessment

HMDMs and undifferentiated THP‐1 cells were seeded into 96‐well plates at a density of 10^6^ cells mL^−1^ (100 µL per well). ENMs were freshly prepared and the following working concentrations were applied: 0.78, 1.56, 3.13, 6.25, 12.5, 25, 50, 100 µg mL^−1^ in 100 µL of complete RPMI cell medium supplemented with 10% FBS. Dimethyl sulfoxide (DMSO) 5%, LPS (100 ng mL^−1^), and culture media alone were used as positive controls for cell death, immune activation, and as a negative control, respectively. After differentiation of HMDMs, the cell medium was removed, and cells were exposed directly to 100 µL of the treatment solutions prepared at the final working concentration. For THP‐1 cells, 100 µL of a twice concentrated solution was added to the cells. After 24 h, 100 µL of the supernatants were collected into 96‐well plates and stored at −80 °C for subsequent analysis by multiplex immunoassay (see below). The cytotoxic effects were evaluated by incubating cells with the Alamar Blue reagent (ThermoFischer Scientific) for 4 h at 37 °C. The resulting fluorescence was determined at 540/590 nm (ex/em), using an Infinite 200 Tecan microplate reader. The statistical analysis was performed by one‐way ANOVA analysis using Prism 5.02 (GraphPad), on results obtained from three independent experiments (three individual donors in case of HMDMs), each performed in triplicate.

##### Cytokine Measurements

For the multiplex immunoassay, HMDMs and THP‐1 cells were exposed to ENM doses inducing 10–15% cell death (EC_10_). The procedure followed the Bio‐Plex Pro Human Cytokine Immunoassay 27‐plex, Group I (BioRad LUMINEX) protocol, as described.^[^ ^57^ ^]^ Briefly, the beads were first dispensed into a 96‐well plate and incubated with antigen standards or samples for 1 h at room temperature. The beads were then washed, followed by incubation with the biotinylated detection antibodies for 30 min at room temperature. After washing the unbound biotinylated antibodies, beads were incubated with the reporter SA‐PE conjugate for 10 min at room temperature. Following removal of the excess of SA‐PE, the beads were passed through the Bio‐Plex 200 array reader to detect the 27 cytokines, chemokines, and growth factors (IL‐1*β*, IL‐1ra, IL‐2, IL‐4, IL‐5, IL‐6, IL‐7, IL‐8, IL‐9, IL‐10, IL‐12, IL‐13, IL‐15, IL‐17, Eotaxin, FGF basic, G‐CSF, GM‐CSF, IFN‐*γ*, IP‐10, MCP‐1, MIP‐1*α*, PDGF, MIP‐1*β*, RANTES, TNF‐*α*, and VEGF) by measuring the fluorescence of the bound SA‐PE. Data processing was performed with the Bio‐Plex Manager v6.0 software. The calibration curves were determined by adjusting logistic functions to the standard points assessed and limits of detection were established. Data retrieved from the multiplex assay were analyzed using hierarchical clustering analysis.^[^ ^18^ ^]^ Pathway analysis was performed on normalized cytokine expression data using the Ingenuity Pathway Analysis (IPA) software (Ingenuity Systems, Redwood City, CA).

##### DNA Damage/Cell Cycle Analysis

THP‐1 cells were exposed for 24 h to the indicated ENMs at EC_10_ concentrations. Etoposide (10 × 10^−6^ m) was used as a positive control. Detection of cellular *γ*H2AX and simultaneous cell cycle analysis were carried out by using the FlowCellect Cell Cycle Checkpoint H2A.X DNA Damage Kit (Millipore) according to the manufacturer's instructions. Briefly, cells were washed twice with phosphate‐buffered saline (PBS), fixed, and permeabilized with the fixation and permeabilization buffers, respectively, and thereafter stained with an anti‐phospho‐Histone H2A.X (Ser139)‐AlexaFluor 488 conjugated‐antibody. DNA staining was performed by using a PI/RNAse solution. Histone H2A.X resides downstream of the DNA damage kinase signaling cascade and phosphorylation of H2A.X at serine 139 is an important indicator of DNA damage.^[^ ^58^ ^]^ Samples were analyzed on a BD LSRFortessa and data were processed using FCS Express DeNovo software.

##### Nucleic Acid Binding Studies

Qiagen AllPrep DNA/RNA/miRNA Universal kit (Qiagen GmbH) was used to extract RNA and DNA according to the manufacturer's protocol. Briefly, cells were quickly washed once with ice‐cold PBS, and lysed directly into the well with 350 µL RTL plus buffer from the kit supplemented with *β*‐mercaptoethanol. Samples were stored at −80 °C before RNA/DNA extraction. RNA was eluted with 30 µL of RNAse free water and DNA was eluted with 50 µL of elution buffer (EB). Au NPs were dispersed by sonication for 5 min in a bath sonicator at 47 kHz before they were diluted to working suspensions (5.0, 0.5, 0.05, 0.005, 0.0005, and 0.00 005 µg mL^−1^). For RNA binding, 25 ng of total RNA from THP‐1 cells was incubated with each of the six EMNs in 100 µL total volume using PBS as buffer. The incubation was performed on ice for 15 or 30 min. Then, the suspension was centrifuged with 16 000 × g for 30 min at 4 °C and the supernatant was collected. ENMs were then washed four times with 1 mL of ice‐cold PBS, centrifuged after each wash with 16 000 × g for 30 min at 4 °C and the supernatants were saved. The washed ENMs were resuspended into 100 µL of PBS. During this time, 25 ng total RNA in a volume of 100 µL was kept on ice to be used as unbound control. The experiments were repeated three times. For DNA binding, 50 ng of DNA from THP‐1 cells was incubated with each of the six ENMs in a volume of 10 µL diluted in water for 15 min on ice; 50 ng DNA in a volume of 10 µL without EMNs was used as unbound control.

##### Quantitative Real‐Time PCR

cDNA was synthesized with Maxima First Strand cDNA Synthesis Kit (K1642; Thermo Scientific) according to manufacturer's instructions. 12 µL of template (control RNA, first supernatant, wash supernatants, final suspension of ENMs, or water as no‐template control) was used in each reaction. Quantitative real‐time PCR (qRT‐PCR) was analyzed with undiluted cDNA using SYBR Green primers for human *β*‐actin and GAPDH (0.2 × 10^−6^ m) and the FastStart Universal SYBR Green Master Mix (Roche Diagnostics). The following SYBR Green primers were used: *β*‐actin forward: 5’‐GACGACATGGAGAAAATCTG‐3; *β*‐actin reverse: ´5’‐ATGATCTGGGTCATCTTCTC‐3´, GAPDH forward: 5´‐ACGGATTTGGTCGTATTGGG‐3; and GAPDH reverse: ´5´‐TGATTTTGGAGGGATCTCGC‐3´. Human placenta cDNA was used as positive control and water as negative control. Reactions were amplified by using the 7500 Fast Real‐Time PCR system (Applied Biosystems). Agarose gel electrophoresis. PCR was run in the presence of 1 × PCR buffer, 1.5 × 10^−3^ m MgCl_2_, 0.4 × 10^−3^ m dNTPs, 1 × 10^−6^ m of each primer, and 8.5 units HotStar Taq *Plus* DNA Polymerase (Qiagen GmbH). The whole DNA‐ENM incubation reaction (10 µL) was used as a template for PCR. The primers used were *LDLR* forward: CACCTGGCTGTTTCCTTGAT; and *LDLR* reverse: CGGTCAGGGGATATGAGTCT. Reactions were amplified by using the T100 Thermal Cycler (BioRad) and a program of: 95 °C for 5 min; 35 rounds of 95 °C for 30 s, 62 °C for 30 s, and 72 °C for 1 min; finishing with 5 min of elongation at 72 °C. The products were visualized on agarose gel (1%) electrophoresis together with Thermo Scientific GeneRuler 100 bp Plus DNA Ladder (Thermo Scientific). DLS and zeta potential measurements were performed on freshly prepared ENM suspensions as described above. Measurements were done for ENMs alone versus ENMs (5 µg) incubated with RNA (25 ng) or DNA (50 ng).

##### Proteomics Analysis

For proteomics samples, 10^6^ cells per well were seeded in a 12‐well plate one day prior to exposure. Cells were exposed for 24 h to the respective EC_10_ doses of freshly resuspended ENMs. Staurosporine (at the EC_10_ dose equivalent), LPS (100 ng mL^−1^), and cell culture medium were used as positive controls for cell death, immune activation, and as a negative control, respectively. Each condition was performed in triplicate. After exposure, the cells were washed twice with PBS by centrifugation 300 g, 5 min at room temperature. Proteins were extracted, digested, and analyzed using a Q Exactive Plus Hybrid Quadrupole‐Orbitrap mass spectrometer. Data processing was done using Raw2MGF and ClusterMGF from the Quanti workflow 18. The data were searched against the human complete proteome database (Uniprot) using the Mascot search engine and quantified by Quant, an in‐house developed software for label‐free quantification. Student's *t*‐test, adjusted for multiple testing false discovery rate, where used to compare the different treatments. Heatmaps were generated by calculating: i) the median of each sample, ii) the variance from the median, and iii) unsupervised clustering of the top 50 or 100 proteins identified for each of the tested ENMs.

##### Microarray Analysis

For transcriptomics, 10^6^ cells per well were seeded in a 12‐well plate one day prior to exposure. Cells were exposed for 24 h to the respective EC_10_ doses of freshly resuspended ENMs and to a fixed concentration for the noncytotoxic ENMs. Cell culture medium without ENMs was used as negative control. Each condition was performed in triplicate. After exposure, the cells were washed twice with ice‐cold PBS by centrifugation at 300 g, 5 min at 4 °C. RNA extraction was performed using the AllPrep DNA/RNA/miRNA Universal Kit, according to the manufacturer's instruction. RNA samples were labeled with Cy3 or Cy5 (Quick Amp Gene Expression Labelling kit, Agilent). Labeled samples were randomly hybridized to Agilent SurePrint G3Human GE 8 × 60K DNA microarrays, according to the manufacturer's protocol (Agilent). Hybridized slides were scanned using the Agilent microarray scanner (Model G2505C) and raw data were extracted using Agilent feature extraction software (version 12.0.1.1). Downstream analyses of the normalized data^[^ ^59^ ^]^ were performed using the IPA software (Ingenuity Systems, Redwood City, CA, http://www.ingenuity.com—version 33 559 992) to examine the canonical signaling pathways associated with the observed changes.^[^ ^22^ ^]^ Venn diagrams of the differentially expressed genes (DEGs) were plotted with the web‐based tool, Venny 2.1.0.^[^ ^60^ ^]^ GO enrichment analyses were performed by using Enrichr,^[^ ^61^, ^62^ ^]^ and the GO database version 2017b. KEGG (Kyoto Encyclopedia of Genes and Genomes) pathways were analyzed and visualized by using the FunMappOne graphical tool.^[^ ^63^ ^]^

##### Statistical Analysis

Experiments were performed in at least three biological replicates (for primary cells, three individual donors) and samples were analyzed using three technical triplicates. Data are average values ± S.D. Statistical analysis was performed by one‐way ANOVA using Prism 5.02 (GraphPad Software, Inc.), assuming equal variances with *p* < 0.05. For proteomics, transcriptomics, and multiplex immunoassay data analysis, see above.

## Conflict of Interest

The authors declare no conflict of interest.

## Author Contributions

A.G. performed experiments and analyzed data; M.D. performed validation experiments and analyzed data; P.K. performed microarray analyses and analyzed data; V.M. and V.F. performed statistical analyses of the transcriptomics data; J.Y. and R.Z. performed proteomics analyses, T.S. performed nucleic acid binding studies; J.K. coordinated the omics studies, M.C., K.L., Z.A., J.R., M.M., R.H., and S.M. synthesized and characterized ENMs; D.A. and K.K. supervised the material synthesis; K.S. coordinated the project; H.A. supervised the microarray work; D.G. supervised the bioinformatics analyses; B.F. supervised the study, analyzed data, and wrote the paper; all authors approved the final version.

## Supporting information

Supporting Information

## Data Availability

The transcriptomics data have been deposited in the NCBI Gene Expression Omnibus (GEO) database (accession no. GSE148705), while the proteomics data have been deposited to the ProteomeXchange consortium via the PRIDE repository with the dataset identifier: PXD019267. Other relevant source data are available through the data repository of FP7‐NANOSOLUTIONS (http://nanosolutions.cs.unisa.it/login.cgi).

## References

[1] B. Fadeel , L. Farcal , B. Hardy , S. Vázquez‐Campos , D. Hristozov , A. Marcomini , I. Lynch , E. Valsami‐Jones , H. Alenius , K. Savolainen , Nat. Nanotechnol. 2018, 13, 537. 29980781 10.1038/s41565-018-0185-0

[2] K. Paunovska , D. Loughrey , C. D. Sago , R. Langer , J. E. Dahlman , Adv. Mater. 2019, 31, 1902798. 10.1002/adma.201902798 PMC6810779 31429126

[3] Y. Li , J. Wang , F. Zhao , B. Bai , G. Nie , A. E. Nel , Y. Zhao , Natl. Sci. Rev. 2018, 5, 365.

[4] X. Bai , F. Liu , Y. Liu , C. Li , S. Wang , H. Zhou , W. Wang , H. Zhu , D. A. Winkler , B. Yan , Toxicol. Appl. Pharmacol. 2017, 323, 66. 28344110 10.1016/j.taap.2017.03.011 PMC5581002

[5] B. Fadeel , Front Toxicol. 2019, 10.3389/ftox.2019.00001. [Epub ahead of print].

[6] I. Hansjosten , J. Rapp , L. Reiner , R. Vatter , S. Fritsch‐Decker , R. Peravali , T. Palosaari , E. Joossens , K. Gerloff , P. Macko , M. Whelan , D. Gilliland , I. Ojea‐Jimenez , M. P. Monopoli , L. Rocks , D. Garry , K. Dawson , P. J. F. Röttgermann , A. Murschhauser , J. O. Rädler , S. V. Y. Tang , P. Gooden , M. A. Belinga‐Desaunay , A. O. Khan , S. Briffa , E. Guggenheim , A. Papadiamantis , I. Lynch , E. Valsami‐Jones , S. Diabaté , C. Weiss , Arch Toxicol. 2018, 92, 633. 29119250 10.1007/s00204-017-2106-7

[7] S. Y. Shaw , E. C. Westly , M. J. Pittet , A. Subramanian , S. L. Schreiber , R. Weissleder , Proc. Natl. Acad. Sci. USA 2008, 105, 7387. 18492802 10.1073/pnas.0802878105 PMC2396702

[8] V. Kodali , M. H. Littke , S. C. Tilton , J. G. Teeguarden , L. Shi , C. W. Frevert , W. Wang , J. G. Pounds , B. D. Thrall , ACS Nano 2013, 7, 6997. 23808590 10.1021/nn402145t PMC3756554

[9] N. Feliu , P. Kohonen , J. Ji , Y. Zhang , H. L. Karlsson , L. Palmberg , A. Nyström , B. Fadeel , ACS Nano 2015, 9, 146. 25530437 10.1021/nn5061783

[10] P. Falagan‐Lotsch , E. M. Grzincic , C. J. Murphy , Proc. Natl. Acad. Sci. USA 2016, 113, 13318. 27821760 10.1073/pnas.1616400113 PMC5127334

[11] C. Pisani , J. C. Gaillard , V. Nouvel , M. Odorico , J. Armengaud , O. Prat , BMC Genomics 2015, 16, 315. 25895662 10.1186/s12864-015-1521-5 PMC4404697

[12] A. Gallud , K. Klöditz , J. Ytterberg , N. Östberg , S. Katayama , T. Skoog , V. Gogvadze , Y. Z. Chen , D. Xue , S. Moya , J. Ruiz , D. Astruc , R. Zubarev , J. Kere , B. Fadeel , Sci. Rep. 2019, 9, 4366. 30867451 10.1038/s41598-019-40579-6 PMC6416392

[13] M. Faria , M. Björnmalm , K. J. Thurecht , S. J. Kent , R. G. Parton , M. Kavallaris , A. P. R. Johnston , J. J. Gooding , S. R. Corrie , B. J. Boyd , P. Thordarson , A. K. Whittaker , M. M. Stevens , C. A. Prestidge , C. J. H. Porter , W. J. Parak , T. P. Davis , E. J. Crampin , F. Caruso , Nat. Nanotechnol. 2018, 13, 777. 30190620 10.1038/s41565-018-0246-4 PMC6150419

[14] H. S. Leong , K. S. Butler , C. J. Brinker , M. Azzawi , S. Conlan , C. Dufés , A. Owen , S. Rannard , C. Scott , C. Chen , M. A. Dobrovolskaia , S. V. Kozlov , A. Prina‐Mello , R. Schmid , P. Wick , F. Caputo , P. Boisseau , R. M. Crist , S. E. McNeil , B. Fadeel , L. Tran , S. F. Hansen , N. B. Hartmann , L. P. W. Clausen , L. M. Skjolding , A. Baun , M. Ågerstrand , Z. Gu , D. A. Lamprou , C. Hoskins , et al., Nat. Nanotechnol. 2019, 14, 629. 31270452 10.1038/s41565-019-0496-9 PMC6939883

[15] L. Farcal , F. Torres Andón , L. Di Cristo , B. M. Rotoli , O. Bussolati , E. Bergamaschi , A. Mech , N. B. Hartmann , K. Rasmussen , J. Riego‐Sintes , J. Ponti , A. Kinsner‐Ovaskainen , F. Rossi , A. Oomen , P. Bos , R. Chen , R. Bai , C. Chen , L. Rocks , N. Fulton , B. Ross , G. Hutchison , L. Tran , S. Mues , R. Ossig , J. Schnekenburger , L. Campagnolo , L. Vecchione , A. Pietroiusti , B. Fadeel , PLoS One 2015, 10, e0127174. 25996496 10.1371/journal.pone.0127174 PMC4440714

[16] S. Smulders , J. P. Kaiser , S. Zuin , K. L. Van Landuyt , L. Golanski , J. Vanoirbeek , P. Wick , P. H. Hoet , Part. Fibre Toxicol. 2012, 9, 41. 23140310 10.1186/1743-8977-9-41 PMC3546036

[17] S. P. Mukherjee , N. Lozano , M. Kucki , A. E. Del Rio‐Castillo , L. Newman , E. Vázquez , K. Kostarelos , P. Wick , B. Fadeel , PLoS One 2016, 11, e0166816. 27880838 10.1371/journal.pone.0166816 PMC5120825

[18] K. Bhattacharya , G. Kiliç , P. M. Costa , B. Fadeel , Nanotoxicology 2017, 11, 809. 28816564 10.1080/17435390.2017.1363309

[19] S. Tuomela , R. Autio , T. Buerki‐Thurnherr , O. Arslan , A. Kunzmann , B. Andersson‐Willman , P. Wick , S. Mathur , A. Scheynius , H. F. Krug , B. Fadeel , R. Lahesmaa , PLoS One 2013, 8, e68415. 23894303 10.1371/journal.pone.0068415 PMC3718780

[20] S. P. Mukherjee , K. Kostarelos , B. Fadeel , Adv. Healthcare Mater. 2018, 7, 1700815. 10.1002/adhm.201700815 29266859

[21] N. K. Tarasova , A. Gallud , A. J. Ytterberg , A. Chernobrovkin , J. R. Aranzaes , D. Astruc , A. Antipov , Y. Fedutik , B. Fadeel , R. A. Zubarev , J. Proteome Res. 2017, 16, 689. 27973853 10.1021/acs.jproteome.6b00747

[22] A. Krämer , J. Green , J. Pollard , S. Tugendreich , Bioinformatics 2014, 30, 523. 24336805 10.1093/bioinformatics/btt703 PMC3928520

[23] R. J. Jackson , C. U. Hellen , T. V. Pestova , Nat. Rev. Mol. Cell Biol. 2010, 11, 113. 20094052 10.1038/nrm2838 PMC4461372

[24] P. Kinaret , V. Marwah , V. Fortino , M. Ilves , H. Wolff , L. Ruokolainen , P. Auvinen , K. Savolainen , H. Alenius , D. Greco , ACS Nano 2017, 11, 3786. 28380293 10.1021/acsnano.6b08650

[25] The Gene Ontology Consortium , Nucleic Acids Res. 2019, 47, D330. 30395331 10.1093/nar/gky1055 PMC6323945

[26] W. A. Schroder , T. T. Le , L. Major , S. Street , J. Gardner , E. Lambley , K. Markey , K. P. MacDonald , R. J. Fish , R. Thomas , A. Suhrbier , J. Immunol. 2010, 184, 2663. 20130210 10.4049/jimmunol.0902187

[27] W. A. Schroder , T. D. Hirata , T. T. Le , J. Gardner , G. M. Boyle , J. Ellis , E. Nakayama , D. Pathirana , H. I. Nakaya , A. Suhrbier , Sci. Rep. 2019, 9, 12421. 31455834 10.1038/s41598-019-48741-w PMC6712035

[28] C. F. A. Vogel , T. Haarmann‐Stemmann , Curr. Opin. Toxicol. 2017, 2, 109. 28971163 10.1016/j.cotox.2017.02.004 PMC5621755

[29] M. Kanehisa , Y. Sato , M. Furumichi , K. Morishima , M. Tanabe , Nucleic Acids Res. 2019, 47, D590. 30321428 10.1093/nar/gky962 PMC6324070

[30] K. Li , X. Zhao , B. K Hammer , S. Du , Y. Chen , ACS Nano 2013, 7, 9664. 24093667 10.1021/nn402472k

[31] B. Fadeel , Front Immunol. 2019, 10, 133. 30774634 10.3389/fimmu.2019.00133 PMC6367956

[32] X. Cai , J. Dong , J. Liu , H. Zheng , C. Kaweeteerawat , F. Wang , Z. Ji , R. Li , Nat. Commun. 2018, 9, 4416. 30356046 10.1038/s41467-018-06869-9 PMC6200803

[33] J. Zhang , S. Wang , M. Gao , R. Li , S. Liu , ACS Sustainable Chem. Eng. 2018, 6, 10374.

[34] D. Breznan , D. D. Das , C. MacKinnon‐Roy , S. Bernatchez , A. Sayari , M. Hill , R. Vincent , P. Kumarathasan , ACS Nano 2018, 12, 12062. 30475590 10.1021/acsnano.8b04910

[35] L. Wu , Y. Zhang , C. Zhang , X. Cui , S. Zhai , Y. Liu , C. Li , H. Zhu , G. Qu , G. Jiang , B. Yan , ACS Nano 2014, 8, 2087. 24552177 10.1021/nn500376w PMC5586106

[36] M. Orecchioni , D. Bedognetti , L. Newman , C. Fuoco , F. Spada , W. Hendrickx , F. M. Marincola , F. Sgarrella , A. F. Rodrigues , C. Ménard‐Moyon , G. Cesareni , K. Kostarelos , A. Bianco , L. G. Delogu , Nat. Commun. 2017, 8, 1109. 29061960 10.1038/s41467-017-01015-3 PMC5653675

[37] J. S. Suk , Q. Xu , N. Kim , J. Hanes , L. M. Ensign , Adv. Drug Delivery Rev. 2016, 99, 28. 10.1016/j.addr.2015.09.012 PMC4798869 26456916

[38] E. M. Grzincic , J. A. Yang , J. Drnevich , P. Falagan‐Lotsch , C. J. Murphy , Nanoscale 2015, 7, 1349. 25491924 10.1039/c4nr05166a PMC4411964

[39] S. M. Louie , R. D. Tilton , G. V. Lowry , Environ. Sci. Nano 2016, 3, 283.

[40] C. D. Walkey , J. B. Olsen , H. Guo , A. Emili , W. C. Chan , J. Am. Chem. Soc. 2012, 134, 2139. 22191645 10.1021/ja2084338

[41] C. Vogt , M. Pernemalm , P. Kohonen , S. Laurent , K. Hultenby , M. Vahter , J. Lehtiö , M. S. Toprak , B. Fadeel , PLoS One 2015, 10, e0129008. 26444829 10.1371/journal.pone.0129008 PMC4596693

[42] C. D. Walkey , W. C. Chan , Chem. Soc. Rev. 2012, 41, 2780. 22086677 10.1039/c1cs15233e

[43] A. Serra , I. Letunic , V. Fortino , R. D. Handy , B. Fadeel , R. Tagliaferri , D. Greco , Sci. Rep. 2019, 9, 179. 30655578 10.1038/s41598-018-37411-y PMC6336851

[44] V. Zhernovkov , T. Santra , H. Cassidy , O. Rukhlenko , D. Matallanas , A. Krstic , W. Kolch , V. Lobaskin , B. N. Kholodenko , Toxicol. Sci. 2019, 171, 303. 10.1093/toxsci/kfz151 31271423

[45] S. Triboulet , C. Aude‐Garcia , L. Armand , V. Collin‐Faure , M. Chevallet , H. Diemer , A. Gerdil , F. Proamer , J. M. Strub , A. Habert , N. Herlin , A. Van Dorsselaer , M. Carrière , T. Rabilloud , PLoS One 2015, 10, e0124496. 25902355 10.1371/journal.pone.0124496 PMC4406518

[46] M. Ilves , P. A. S. Kinaret , J. Ndika , P. Karisola , V. Marwah , V. Fortino , Y. Fedutik , M. Correia , N. Ehrlich , K. Loeschner , A. Besinis , J. Vassallo , R. D. Handy , H. Wolff , K. Savolainen , D. Greco , H. Alenius , Part Fibre Toxicol. 2019, 16, 28. 31277695 10.1186/s12989-019-0309-1 PMC6612204

[47] I. Karkossa , A. Bannuscher , B. Hellack , A. Bahl , S. Buhs , P. Nollau , A. Luch , K. Schubert , M. von Bergen , A. Haase , Part Fibre Toxicol. 2019, 16, 38. 31653258 10.1186/s12989-019-0321-5 PMC6814995

[48] G. M. Barton , J. C. Kagan , Nat. Rev. Immunol. 2009, 9, 535. 19556980 10.1038/nri2587 PMC3934928

[49] A. N. R. Weber , Z. Bittner , X. Liu , T. M. Dang , M. P. Radsak , C. Brunner , Front Immunol. 2017, 8, 1454. 29167667 10.3389/fimmu.2017.01454 PMC5682317

[50] N. J. Gay , M. F. Symmons , M. Gangloff , C. E. Bryant , Nat. Rev. Immunol. 2014, 14, 546. 25060580 10.1038/nri3713

[51] K. Ezzat , M. Pernemalm , S. Pålsson , T. C. Roberts , P. Järver , A. Dondalska , B. Bestas , M. J. Sobkowiak , B. Levänen , M. Sköld , E. A. Thompson , O. Saher , O. K. Kari , T. Lajunen , E. Sverremark Ekström , C. Nilsson , Y. Ishchenko , T. Malm , M. J. A. Wood , U. F. Power , S. Masich , A. Lindén , J. K. Sandberg , J. Lehtiö , A. L. Spetz , S. El Andaloussi , Nat. Commun. 2019, 10, 2331. 31133680 10.1038/s41467-019-10192-2 PMC6536551

[52] C. C. Ho , Y. H. Luo , T. H. Chuang , C. S. Yang , Y. C. Ling , P. Lin , Toxicology 2013, 308, 1. 23499856 10.1016/j.tox.2013.03.003

[53] C. C. Ho , Y. H. Luo , T. H. Chuang , P. Lin , Toxicology 2016, 344–346, 61. 10.1016/j.tox.2016.02.005 26925925

[54] C. Y. Tsai , S. L. Lu , C. W. Hu , C. S. Yeh , G. B. Lee , H. Y. Lei , J. Immunol. 2012, 188, 68. 22156340 10.4049/jimmunol.1100344

[55] N. Luo , J. K. Weber , S. Wang , B. Luan , H. Yue , X. Xi , J. Du , Z. Yang , W. Wei , R. Zhou , G. Ma , Nat. Commun. 2017, 8, 14537. 28233871 10.1038/ncomms14537 PMC5333105

[56] N. Feliu , M. V. Walter , M. I. Montanez , A. Kunzmann , A. Hult , A. Nyström , M. Malkoch , B. Fadeel , Biomaterials 2012, 33, 1970. 22177621 10.1016/j.biomaterials.2011.11.054

[57] A. Gallud , O. Bondarenko , N. Feliu , N. Kupferschmidt , R. Atluri , A. Garcia‐Bennett , B. Fadeel , Biomaterials 2017, 121, 28. 28063981 10.1016/j.biomaterials.2016.12.029

[58] E. P. Rogakou , D. R. Pilch , A. H. Orr , V. S. Ivanova , W. M. Bonner , J. Biol. Chem. 1998, 273, 5858. 9488723 10.1074/jbc.273.10.5858

[59] B. M. Bolstad , R. A. Irizarry , M. Astrand , T. P. Speed , Bioinformatics 2003, 19, 185. 12538238 10.1093/bioinformatics/19.2.185

[60] J. C. Oliveros , http://bioinfogp.cnb.csic.es/tools/venny/index.html (accessed: 2007).

[61] E. Y. Chen , C. M. Tan , Y. Kou , Q. Duan , Z. Wang , G. V. Meirelles , N. R. Clark , A. Ma'ayan , BMC Bioinformatics 2013, 14, 128. 23586463 10.1186/1471-2105-14-128 PMC3637064

[62] M. V. Kuleshov , M. R. Jones , A. D. Rouillard , N. F. Fernandez , Q. Duan , Z. Wang , S. Koplev , S. L. Jenkins , K. M. Jagodnik , A. Lachmann , M. G. McDermott , C. D. Monteiro , G. W. Gundersen , A. Ma'ayan , Nucleic Acids Res. 2016, 44, W90. 27141961 10.1093/nar/gkw377 PMC4987924

[63] G. Scala , A. Serra , V. S. Marwah , L. A. Saarimäki , D. Greco , BMC Bioinformatics 2019, 20, 79. 30767762 10.1186/s12859-019-2639-2 PMC6376640

[64] S. P. Mukherjee , G. Gupta , K. Klöditz , J. Wang , A. F. Rodrigues , K. Kostarelos , B. Fadeel , Small 2020, 16, 1907686. 10.1002/smll.201907686 32227449

[65] A. F. Rodrigues , L. Newman , D. Jasim , S. P. Mukherjee , J. Wang , I. A. Vacchi , C. Ménard‐Moyon , A. Bianco , B. Fadeel , K. Kostarelos , C. Bussy , Adv. Sci. 2020, 7, 1903200. 10.1002/advs.201903200 PMC7312279 32596109

