# Supplementary material for: Multiparametric Profiling of Engineered Nanomaterials: Unmasking the Surface Coating Effect
Source: Adv Sci (Weinh). 2020 Oct 11;7(22):2002221. doi: 10.1002/advs.202002221 (PMC7675037; doi:10.1002/advs.202002221)
Supplement: Supplementary file 1 — Supporting Information [file ADVS-7-2002221-s001.pdf]

© 2020 Wiley-VCH GmbH

ADVANCED SCIENCE
Open Access

Supporting Information

for *Adv. Sci.,* DOI: 10.1002/advs.202002221

Multiparametric Profiling of Engineered Nanomaterials: Unmasking the Surface Coating Effect

*Audrey Gallud, Mathilde Delaval, Pia Kinaret, Veer Singh Marwah, Vittorio Fortino, Jimmy Ytterberg, Roman Zubarev, Tiina Skoog, Juha Kere, Manuel Correia, Katrin Loeschner, Zahraa Al-Ahmady, Kostas Kostarelos, Jaime Ruiz, Didier Astruc, Marco Monopoli, Richard Handy, Sergio Moya, Kai Savolainen, Harri Alenius, Dario Greco, and Bengt Fadeel**

Supporting Information

Multi-Parametric Profiling of Engineered Nanomaterials: Unmasking the Surface Coating Effect

Audrey Gallud, Mathilde Delaval, Pia Kinaret, Veer Singh Marwah, Vittorio Fortino, Jimmy Ytterberg, Roman Zubarev, Tiina Skoog, Juha Kere, Manuel Correia, Katrin Loeschner, Zahraa Al-Ahmady, Kostas Kostarelos, Jaime Ruiz, Didier Astruc, Marco Monopoli, Richard Handy, Sergio Moya, Kai Savolainen, Harri Alenius, Dario Greco, Bengt Fadeel

ENM	TEM: Estimated primary particle size (L= µm, D= nm)	TEM: Approximate agglomerate/ aggregate size	DLS (Milli-Q H_2O): Z_{ave} ± S.D. (d.nm)	DLS (Milli-Q H_2O): PDI ± S.D.	ζ-potential (Milli-Q H_2O) (mV)	Surface chemistry
MWCNT_core	L: 0.9 - 1.2 / D: 10 - 15	not observed	N.A.		-24 ± 1	not disclosed (Nanocyl^{TM})
$MWCNT\_NH_2$	L: 0.6 - 0.9 / D: 10 - 15	not/few observed	N.A.		-21 ± 1	not disclosed (Nanocyl^{TM})
MWCNT_COOH	L: 1.2 - 1.5 / D: 10 - 15	not/few observed	N.A.		-30 ± 2	not disclosed (Nanocyl^{TM})
MWCNT_PEG	L: 0.6 - 0.9 / D: 10 - 15	not observed	N.A.		-2 ± 1	1,2-distearoyl-sn-glycero-3-phosphoethanolamine-N-[methoxy(PEG)-2000]
CuO_core	D: 10 - 20	400 - 900	1408.3 ± 101.5	0.482 ± 0.124	14.0 ± 1.2	-
CuO_NH_2	D: 10 - 20	30 - 60 and 75 - 400	293.0 ± 7.9	0.309 ± 0.006	27.7 ± 0.5	$-S-(CH_2)_2-NH_3Cl$
CuO_COOH	D: 10 - 20	200 - 2500	1179.3 ± 60.6	0.470 ± 0.024	-7.3 ± 0.5	$-S-CH_2–COOH$
CuO_PEG	D: 10 – 20	10 - 150	90.4 ± 2.5	0.146 ± 0.020	-16.8 ± 0.4	$-S-(CH_2)-C(O)O-(CH_2-CH_2-O-)_{12}-CH_3$
TiO2s_core	D: < 4 *	N.D. **	23.03 ± 0.68	0.421 ± 0.042	21.5 ± 0.5	NO_3^- counter ion
$TiO2s\_NH_2$	D: < 4 *	N.D. **	23.36 ± 0.46	0.239 ± 0.048	14.2 ± 0.9	$-(O_3)Si-(CH_2)_3-NH_3Cl$
TiO2s_COOH	D: < 4 *	N.D. **	209.13 ± 8.24	0.288 ± 0.042	21.2 ± 1.3	$-(O_3)Si-(CH_2)_3-NH(O)-C-(CH_2)_2-COOH$
TiO2s_PEG	D: < 4 *	N.D. **	38.97 ± 0.9	0.214 ± 0.004	41.1 ± 1.5	$-(O_3)Si-(CH_2)_3-NH(O)-(CH_2-CH_2-O)_{12}-CH_3$
TiO2r_core	L: 56.5 ± 25.5 / D: 4 - 15	Bundles	N.A.		16 ± 1	-
$TiO2r\_NH_2$	L: 94.8 ± 43.5 / D: 4 - 15	Bundles	N.A.		25 ± 3	$-(O_3)Si-(CH_2)_3-NH_3Cl$
TiO2r_COOH	L: 77.7 ± 46.6 / D: 4 - 15	Bundles	N.A.		-23 ± 1	$-(O_3)Si-(CH_2)_3-NH(O)-C-(CH_2)_2-COOH$
TiO2r_PEG	L: 69.9 ± 26.7 / D: 4 - 15	Bundles	N.A.		26 ± 2	$-(O_3)Si-(CH_2)_3-NH(O)-(CH_2-CH_2-O)_{12}-CH_3$
QD_NH_2	D: < 4 *	not observed	N.D. ***		9 ± 1	$-S-(CH_2)_2-NH_3Cl$
QD_COOH	D: < 4 *	30 - 2	N.D. ***		-33 ± 5	$-S-(CH)(COOH)-CH_2–COOH$
QD_PEG	D: < 4 *	not observed	N.D. ***		-25 ± 4	$-S-CH_2-C(O)O-(CH_2-CH_2-O)n-CH_3$
Ag_NR3+	D: 2 - 8 and 50 - 110	not observed	80.3 ± 7.9	0.447 ± 0.181	44.6 ± 1.6	$-S(CH_2)_{11}N(CH_3)_3Br$
Ag_COOH	D: 5 - 15	100 - 500	164.7 ± 60.6	0.164 ± 0.013	-32.5 ± 1.1	$-S-(CH_2)_{10}–COOH$
Ag_PEG	D: 2 - 5, 7 - 15 and 200 - 100	not observed	37.7 ± 2.5	0.430 ± 0.014	-10.7 ± 0.4	$-S(PEG)_{550}CH_3$
Au5-NR3+	D: 1 - 8	not observed	N.D. ***		49.0 ± 0.8	$-S(CH_2)_{11}N(CH_3)_3Br$ and $-S(CH_2)_{11}CH_3$
Au5-COOH	D: 1 - 4	not observed	N.D. ***		-28.8 ± 0.4	$-S(CH_2)_{10}CO_2Na$
Au5-PEG	D: 2 - 6	not observed	N.D. ***		-16.5 ± 3	$-SPEG_{550}$
Au20-NR3+	D: 9 - 20	suspected	N.D. ***		42.8 ± 10	$-S(CH_2)_{11}N(CH_3)_3Br$
Au20-COOH	D: 10 - 20 and 32 - 54	not observed	N.D. ***		-26 ± 3	$-S(CH_2)_{10}CH_2O(C_2H_4O)_3C_2H_4OCH_2CO_2Na$
Au20-PEG	D: 10 - 18	not observed	N.D. ***		-37 ± 3	$-SPEG_{550}$
ND_NH_2	D: < 4 *	N.D. **	N.D. ***		12 ± 1	$-C(O)-NH-(CH_2)_2NH-(CH_2)_2NH-(CH_2)_2-NH_3Cl$
ND_COOH	D: < 4 *	N.D. **	N.D. ***		-18 ± 1	-COOH
ND_PEG	D: < 4 *	N.D. **	N.D. ***		-5 ± 1	$-C(O)O-CH_2CH_2-(O-CH_2-CH_2)_{11}-O-CH_3$

Table S1. Physicochemical properties of ENMs. Primary sizes and hydrodynamic particle sizes were determined by using transmission electron microscopy (TEM) and dynamic light scattering (DLS), respectively. N.D.: not determined, N.A.: not applicable, L: length, D: diameter, * primary particle size was below the resolution of the microscope, ** in the case of TiO_2 spheres the agglomerate/aggregate size estimated from TEM may be an artifact due to drying and therefore is not reported, *** in the case of the Au5, Au20, and QDs there were limitations with the DLS measurement due to their small size.

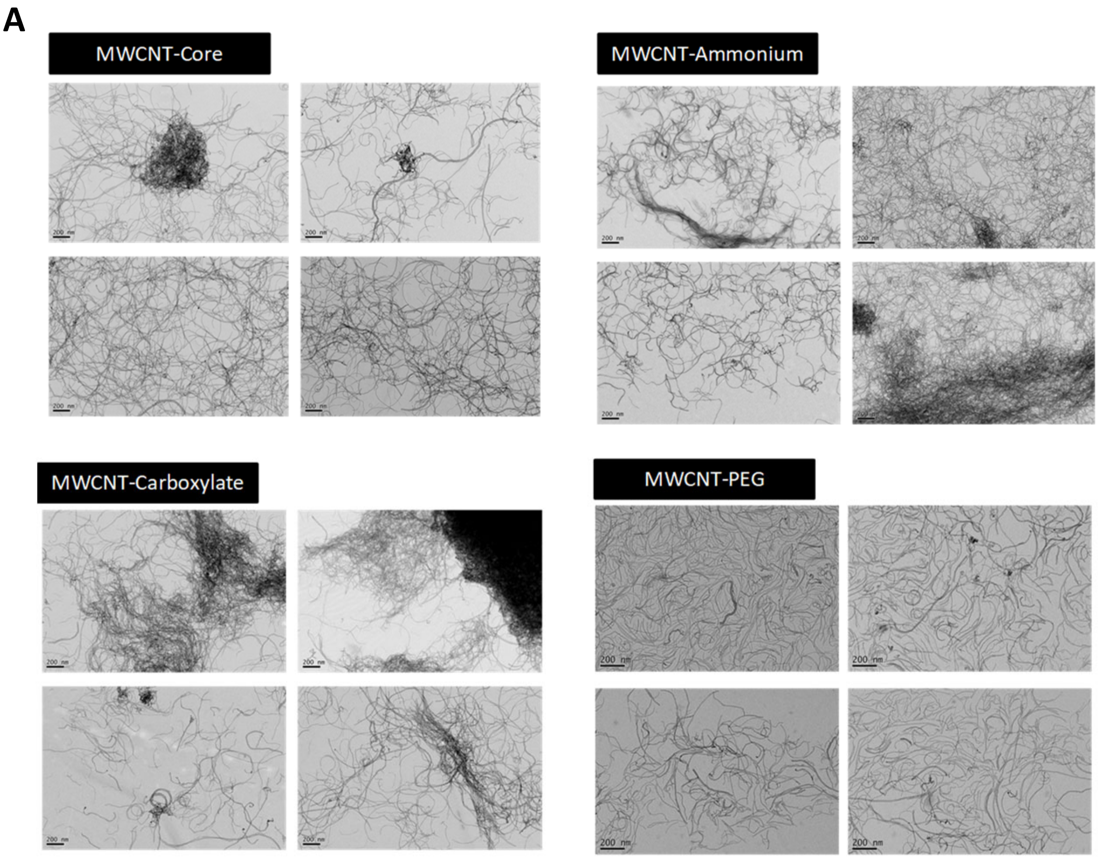

Figure S1 (A). TEM images of MWCNT suspensions in 0.1% albumin. Stock suspensions were prepared at 2 mg/mL in 0.1% albumin then diluted to 0.5 mg /mL in milli-Q water followed by 10 min sonication before TEM imaging.

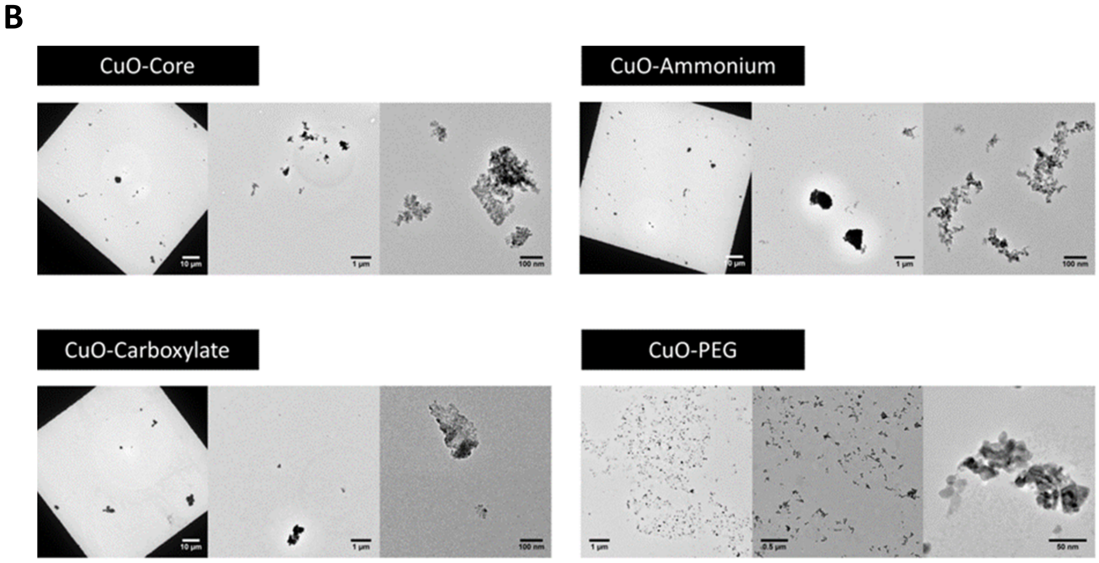

Figure S1 (B). TEM images of CuO ENMs suspensions. CuO stock suspensions were prepared by ultrasonic treatment with tip sonication of 1 mg/mL suspensions (CuO_core, CuO_COOH, and CuO_NH) or 6.5 mg/mL (CuO_PEG). The CuO ENMs suspensions were then diluted to 10 μg/mL prior to TEM imaging.

C

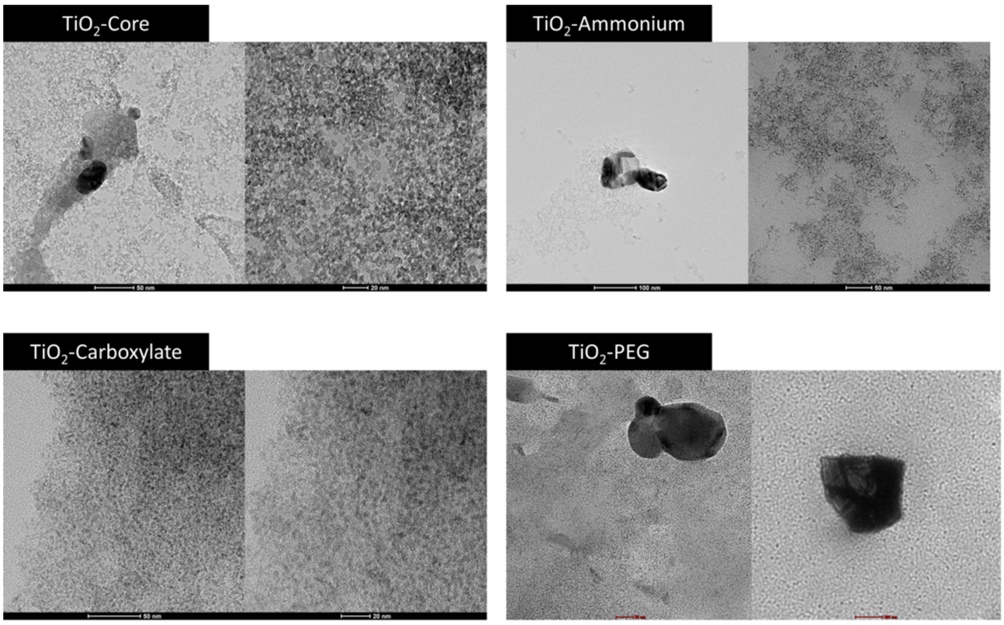

Figure S1 (C). TEM images of TiO_2 sphere suspensions. TiO_2s stock suspensions were prepared by ultrasonic treatment with bath sonication of 5 mg/mL of TiO_2s_core, TiO_2s_COOH, TiO_2s_NH and TiO_2s_PEG. The TiO_2s suspensions were then diluted to 1 µg/mL prior to TEM imaging.

D

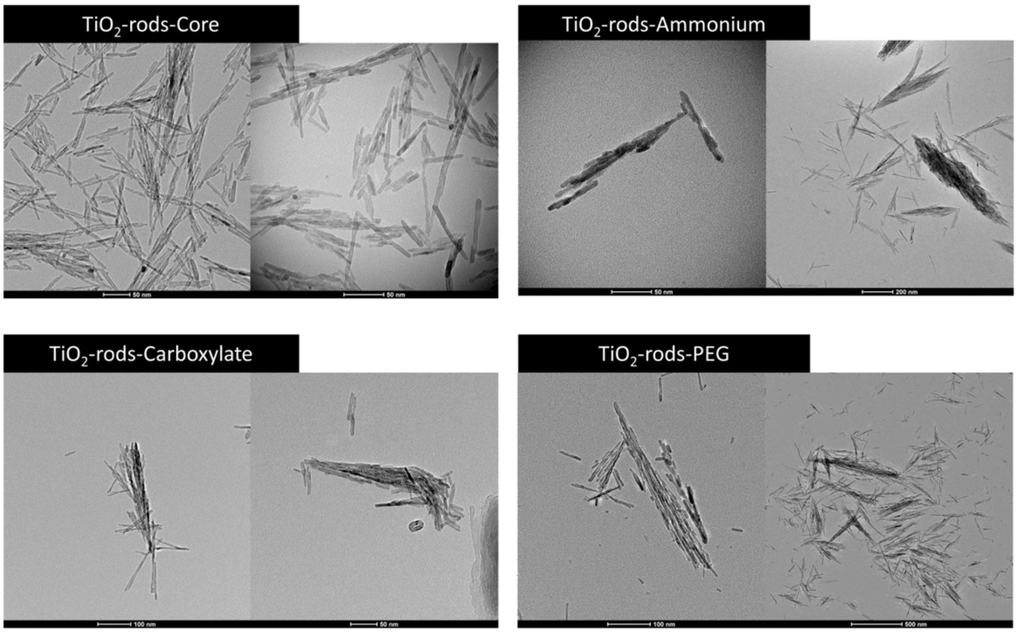

Figure S1 (D). TEM images of TiO_2 rods suspensions. TiO_2r stock suspensions were prepared by bath sonication of 5 mg/mL suspensions for 1 min. The TiO_2r suspensions were then diluted to 1 µg/mL prior to TEM imaging.

E

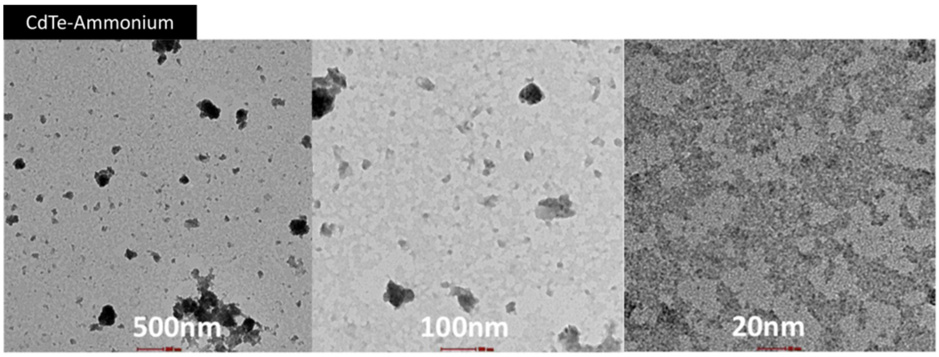

Figure S1 (E). TEM images of QD_NH suspensions. QD_NH ENMs stock suspension was prepared by gentle vortex treatment and brief bath sonication at 5 mg/mL and then diluted to 10 μg/mL prior to TEM imaging.

F

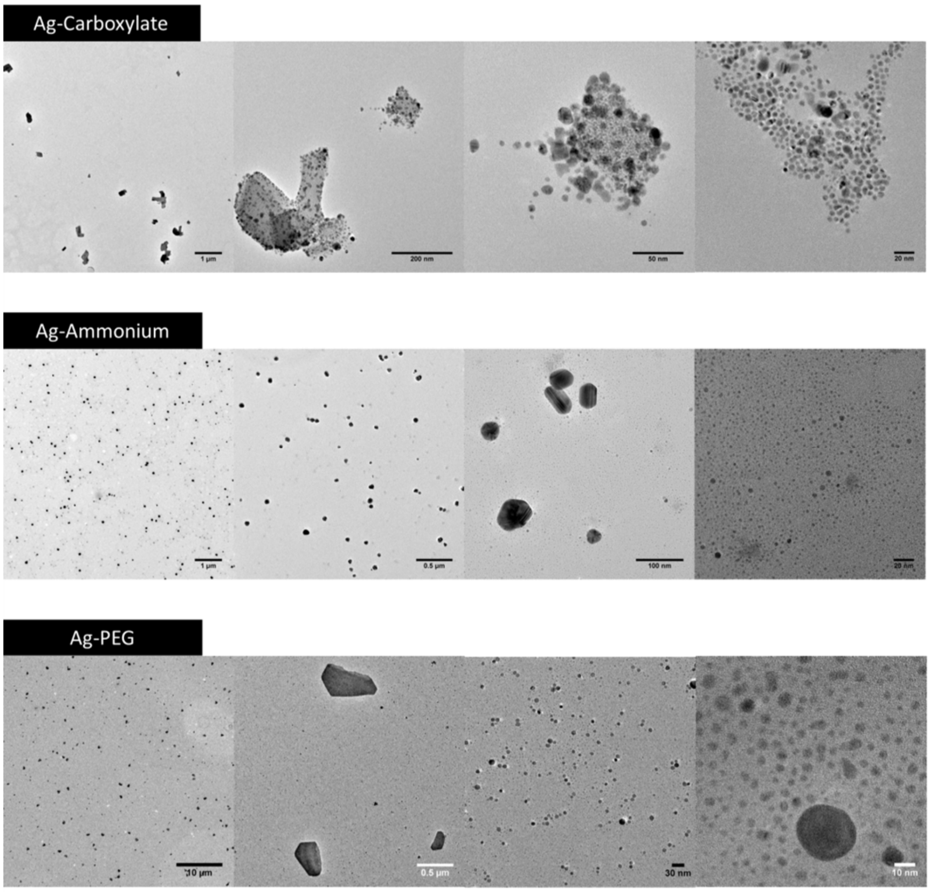

Figure S1 (F). TEM images of Ag ENMs suspensions. Ag ENM stock suspensions (Ag_COOH, 10 mg/mL; Ag_NR3+, 2.5 mg/mL; Ag_PEG, 5 mg/mL) were diluted in milli-Q water and working suspensions were diluted to 20 μg/mL prior to TEM imaging.

G

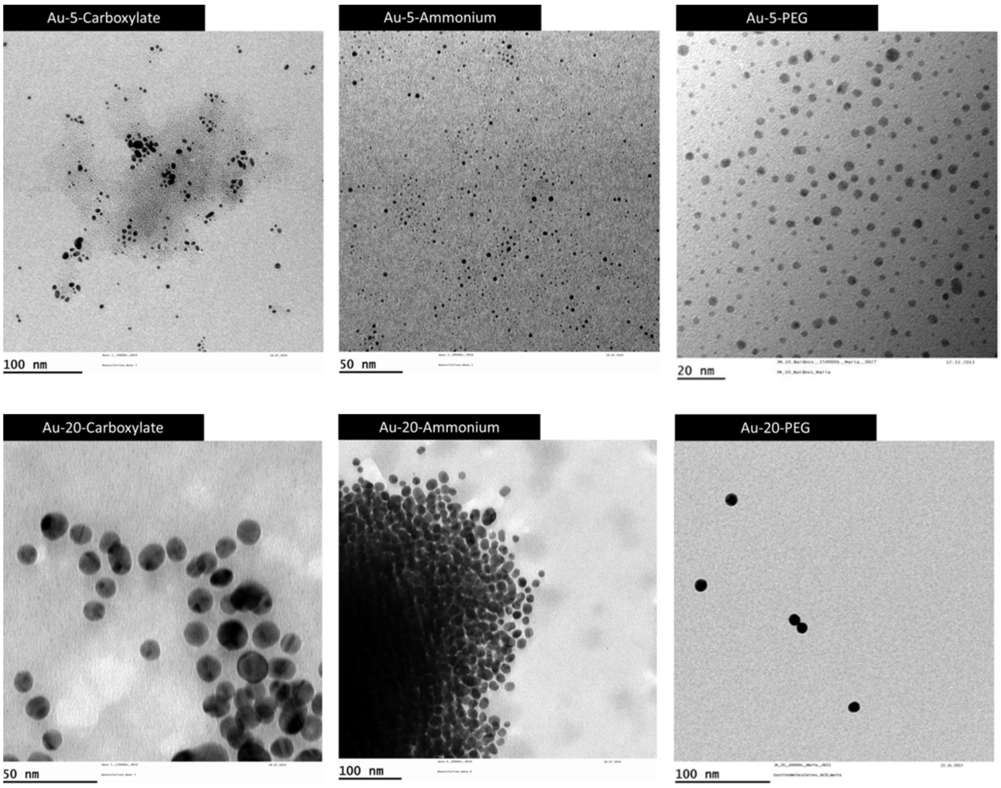

Figure S1 (G). TEM images of Au5 and Au20 ENM suspensions. The concentration used for TEM imaging was 0.1 mg/mL.

H

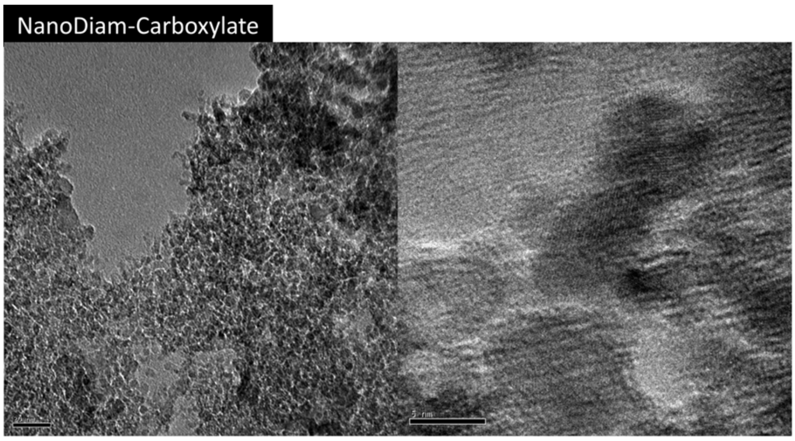

Figure S1 (H). TEM of the native NDs with -COOH groups. No difference between these particles and the other modified variants ND_NH and ND_PEG were observed (data not shown). The ND_COOH suspension was diluted to 0.1 mg/mL prior to TEM imaging.

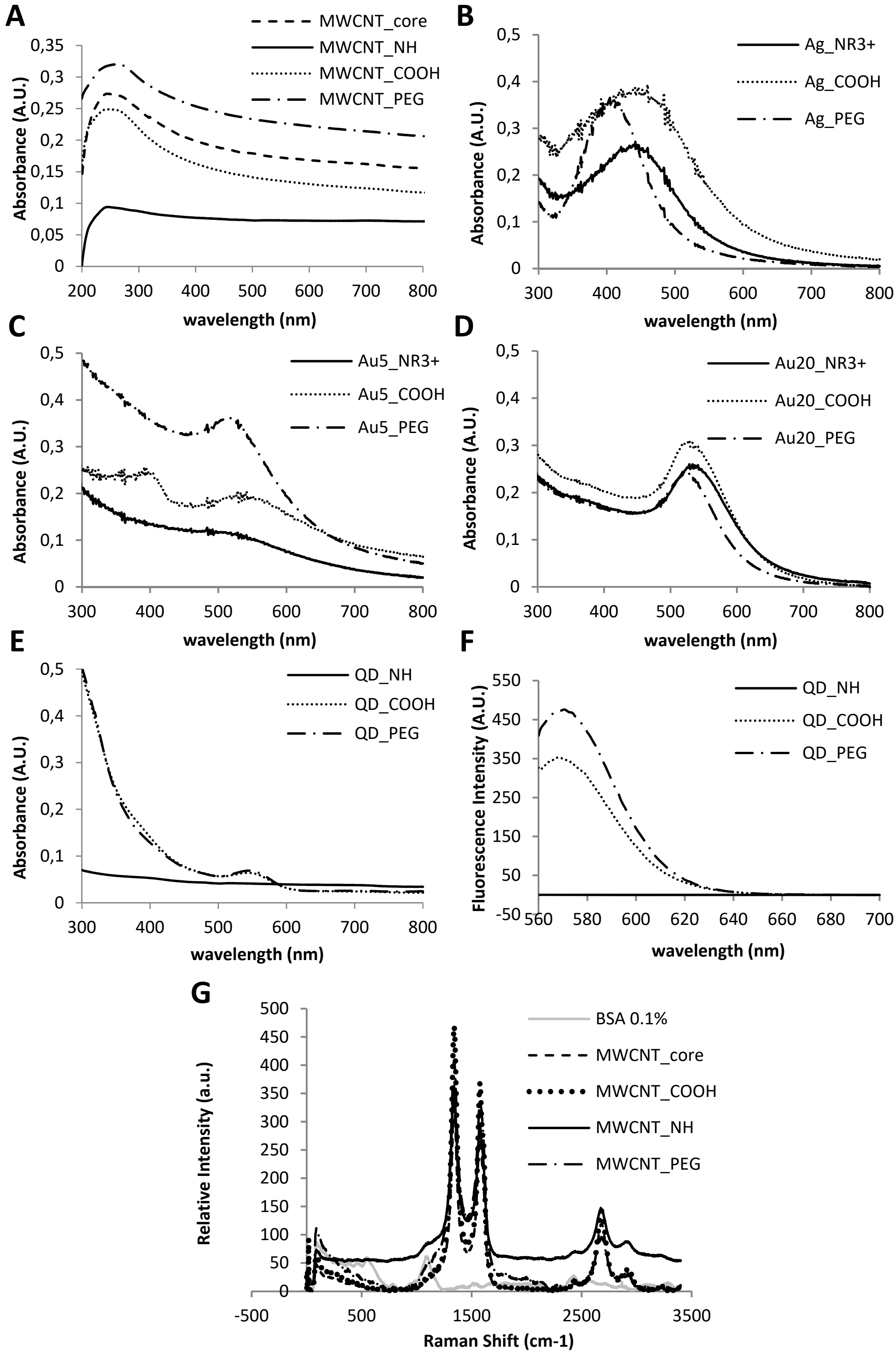

Figure S2. Optical properties of ENMs measured by UV-vis, photoluminescence, or Raman spectrometry. UV-vis measurements were performed for (A) MWCNTs (core and surface modified), (B) Ag ENMs, (C) Au5 ENMs, (D) Au20 ENMs, and (E) QDs. (F) Photoluminescence analysis was performed for the QDs. (G) Raman spectroscopy was performed on the MWCNTs.

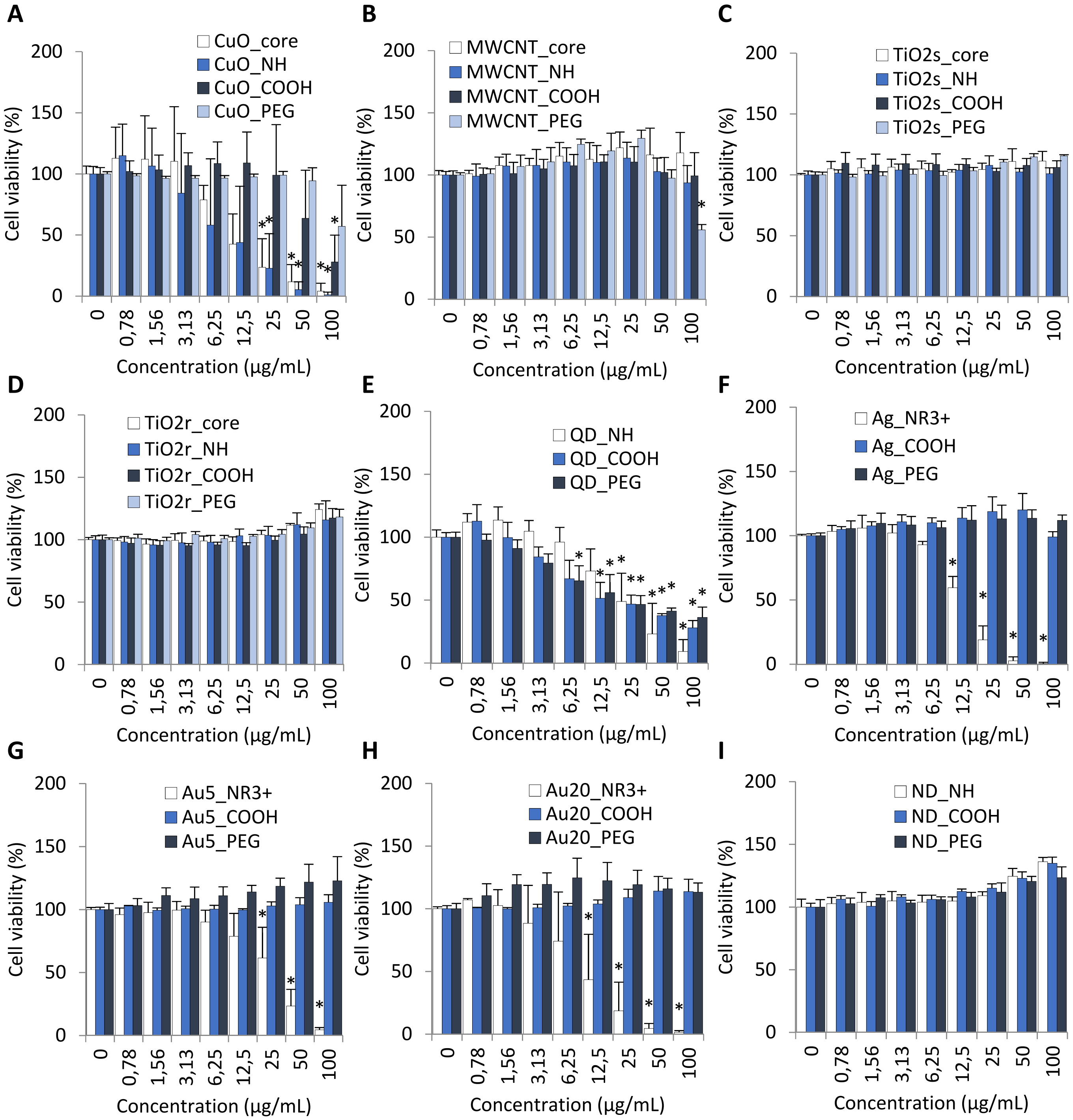

Figure S3. Cytotoxicity assessment of 31 ENMs using primary human monocyte-derived macrophages (HMDMs). Cell viability was evaluated by using the Alamar Blue assay following 24 h exposure to (A) CuO ENMs, (B) MWCNTs, (C) TiO_2 spheres, (D) TiO_2 rods, (E) QDs, (F) Ag ENMs, (G) Au-5 nm ENMs, (H) Au-20 nm ENMs, and (I) NDs at the indicated concentrations. ENMs were surface modified with carboxyl/carboxylate groups (COOH/COO^-), amino/ammonium groups ($-NH_2$/$-NR^{3+}$) or PEG, as indicated. Data are mean values ± S.D. using cells from three different donors (in triplicate). *$p < 0.05$.

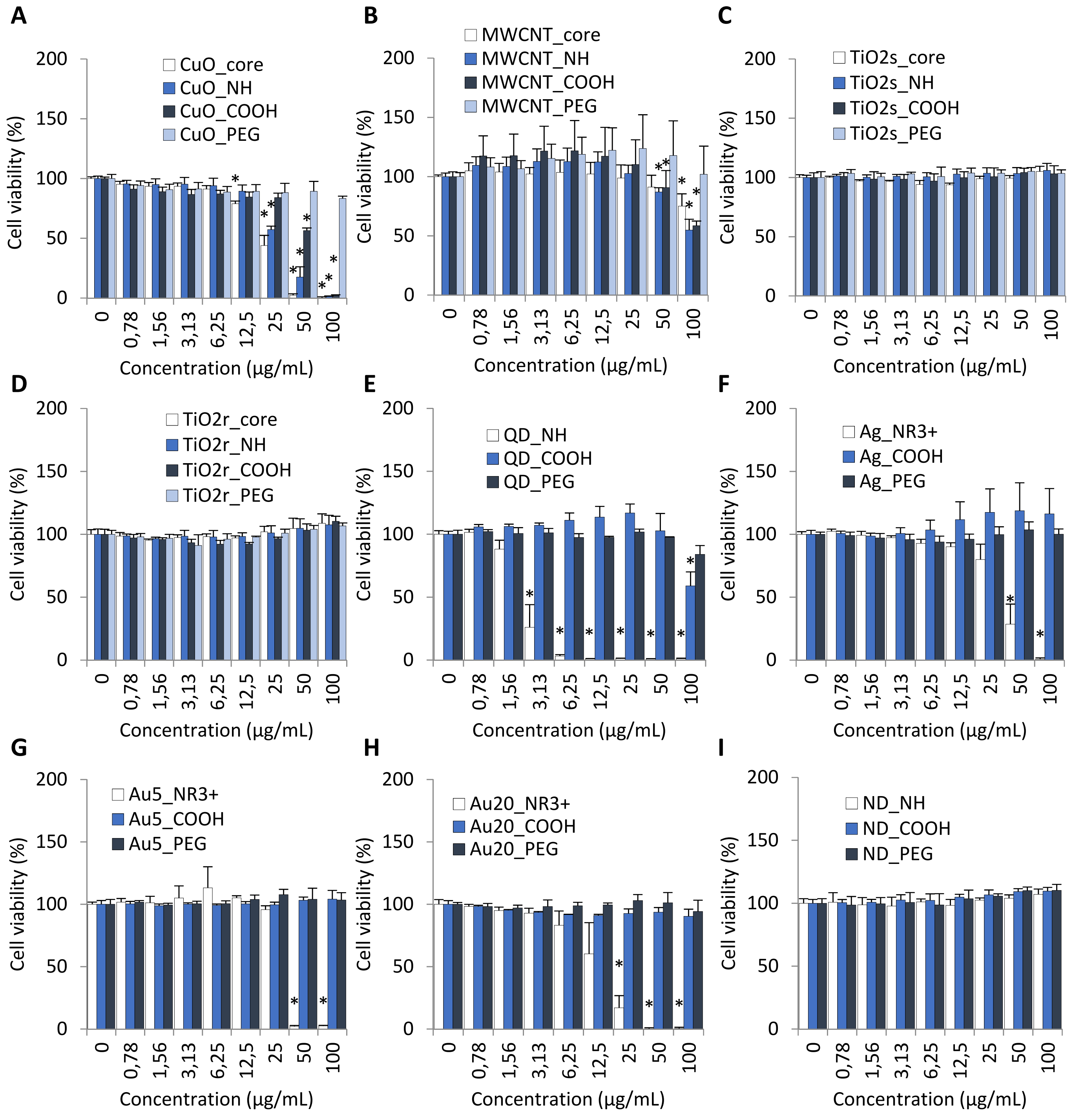

Figure S4. Cytotoxicity assessment of 31 ENMs using the human monocytic cell line THP-1. Cell viability was evaluated by using the Alamar Blue assay following 24 h exposure to (A) CuO ENMs, (B) MWCNTs, (C) TiO_2 spheres, (D) TiO_2 rods, (E) QDs, (F) Ag ENMs, (G) Au-5 nm ENMs, (H) Au-20 nm ENMs and (I) NDs at the indicated concentrations. ENMs were surface modified with carboxyl/carboxylate groups ($COOH/COO^-$), amino/ammonium groups ($-NH_2/-NR^{3+}$) or PEG, as indicated. Data reported as mean values ± S.D. of three independent experiments (each performed in triplicate). *$p < 0.05$.

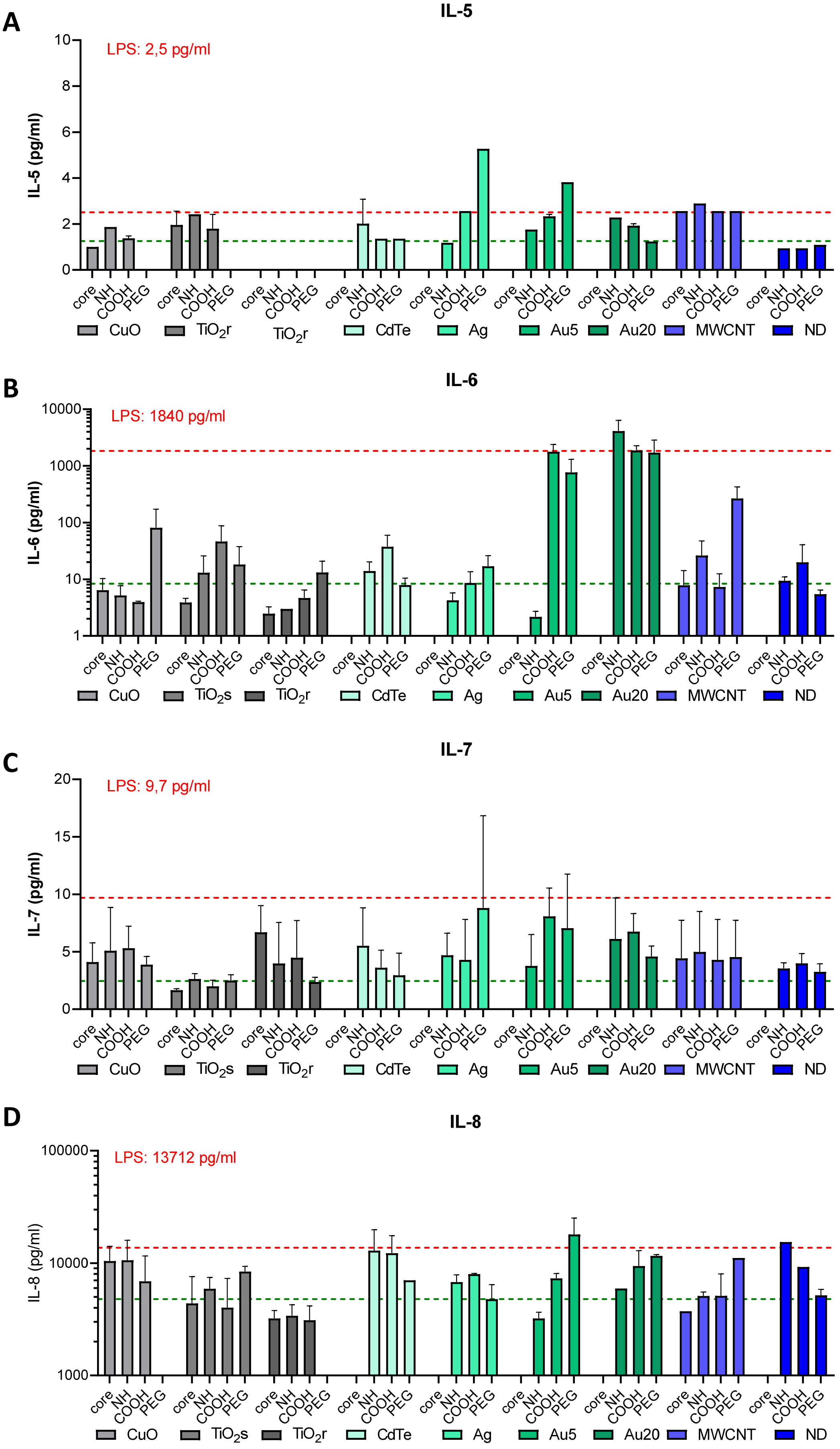

A
IL-5
LPS: 2,5 pg/ml
IL-5 (pg/ml)
0
2
4
6
8
10
core NH COOH PEG
CuO
TiO2r
TiO2r
CdTe
Ag
Au5
Au20
MWCNT
ND
B
IL-6
LPS: 1840 pg/ml
IL-6 (pg/ml)
1
10
100
1000
10000
core NH COOH PEG
CuO
TiO2s
TiO2r
CdTe
Ag
Au5
Au20
MWCNT
ND
C
IL-7
LPS: 9,7 pg/ml
IL-7 (pg/ml)
0
5
10
15
20
core NH COOH PEG
CuO
TiO2s
TiO2r
CdTe
Ag
Au5
Au20
MWCNT
ND
D
IL-8
LPS: 13712 pg/ml
IL-8 (pg/ml)
1000
10000
100000
core NH COOH PEG
CuO
TiO2s
TiO2r
CdTe
Ag
Au5
Au20
MWCNT
ND

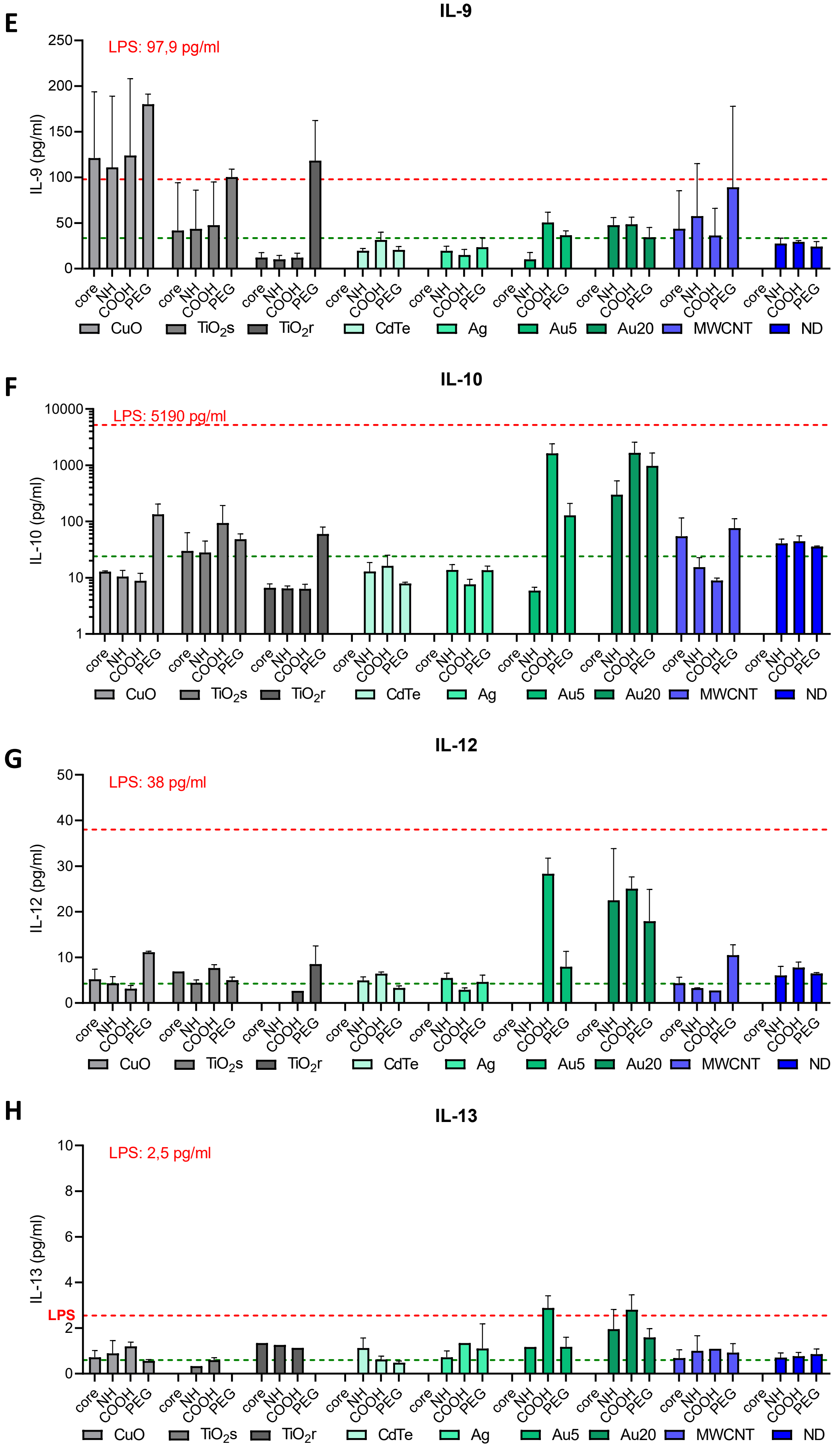
E
IL-9
LPS: 97,9 pg/ml
IL-9 (pg/ml)
F
IL-10
LPS: 5190 pg/ml
IL-10 (pg/ml)
G
IL-12
LPS: 38 pg/ml
IL-12 (pg/ml)
H
IL-13
LPS: 2,5 pg/ml
IL-13 (pg/ml)
LPS
core
NH
COOH
PEG
CuO
TiO2s
TiO2r
CdTe
Ag
Au5
Au20
MWCNT
ND

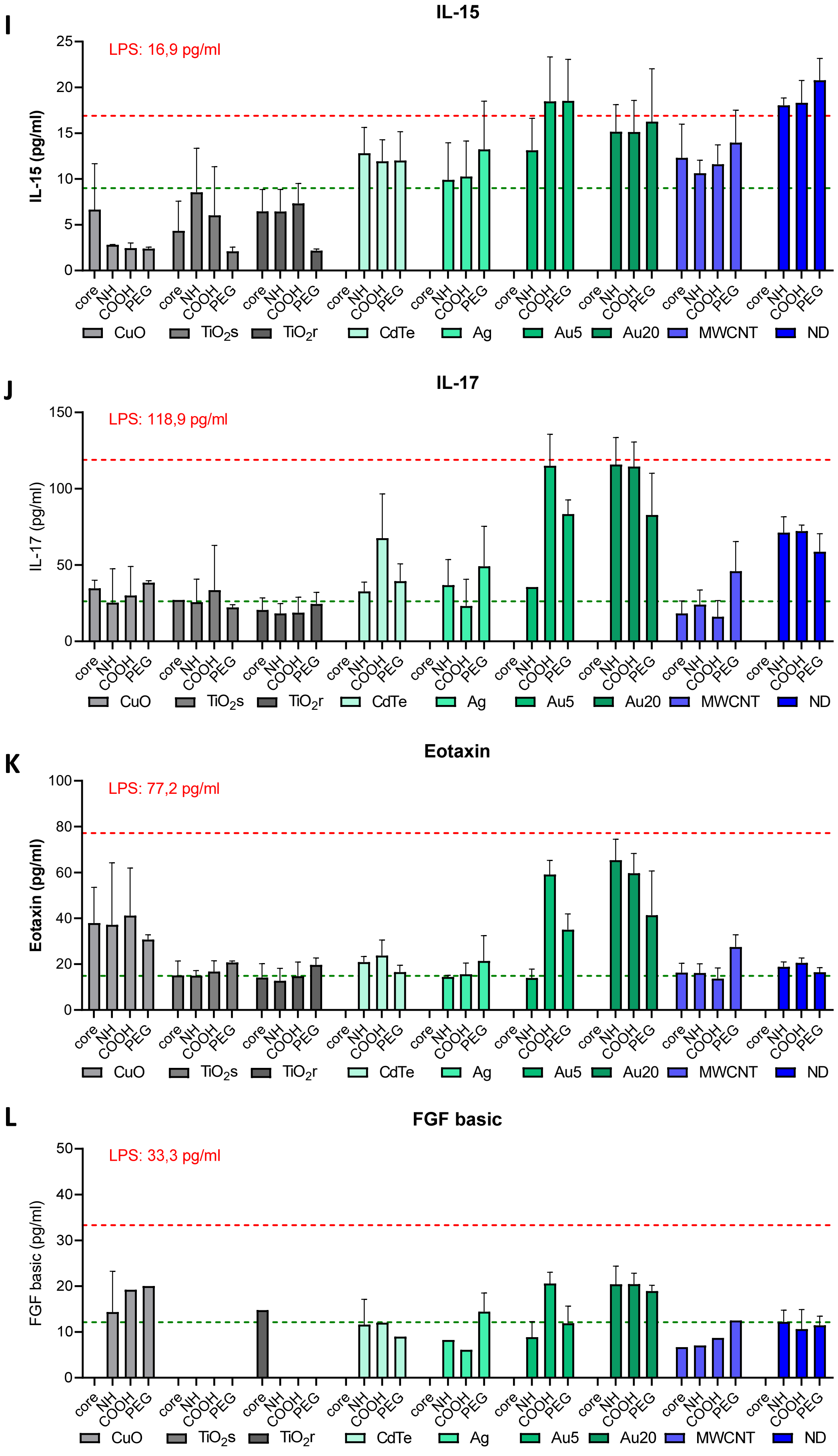
I
IL-15
IL-15 (pg/ml)
LPS: 16,9 pg/ml
core NH COOH PEG
CuO TiO2s TiO2r CdTe Ag Au5 Au20 MWCNT ND
J
IL-17
IL-17 (pg/ml)
LPS: 118,9 pg/ml
core NH COOH PEG
CuO TiO2s TiO2r CdTe Ag Au5 Au20 MWCNT ND
K
Eotaxin
Eotaxin (pg/ml)
LPS: 77,2 pg/ml
core NH COOH PEG
CuO TiO2s TiO2r CdTe Ag Au5 Au20 MWCNT ND
L
FGF basic
FGF basic (pg/ml)
LPS: 33,3 pg/ml
core NH COOH PEG
CuO TiO2s TiO2r CdTe Ag Au5 Au20 MWCNT ND

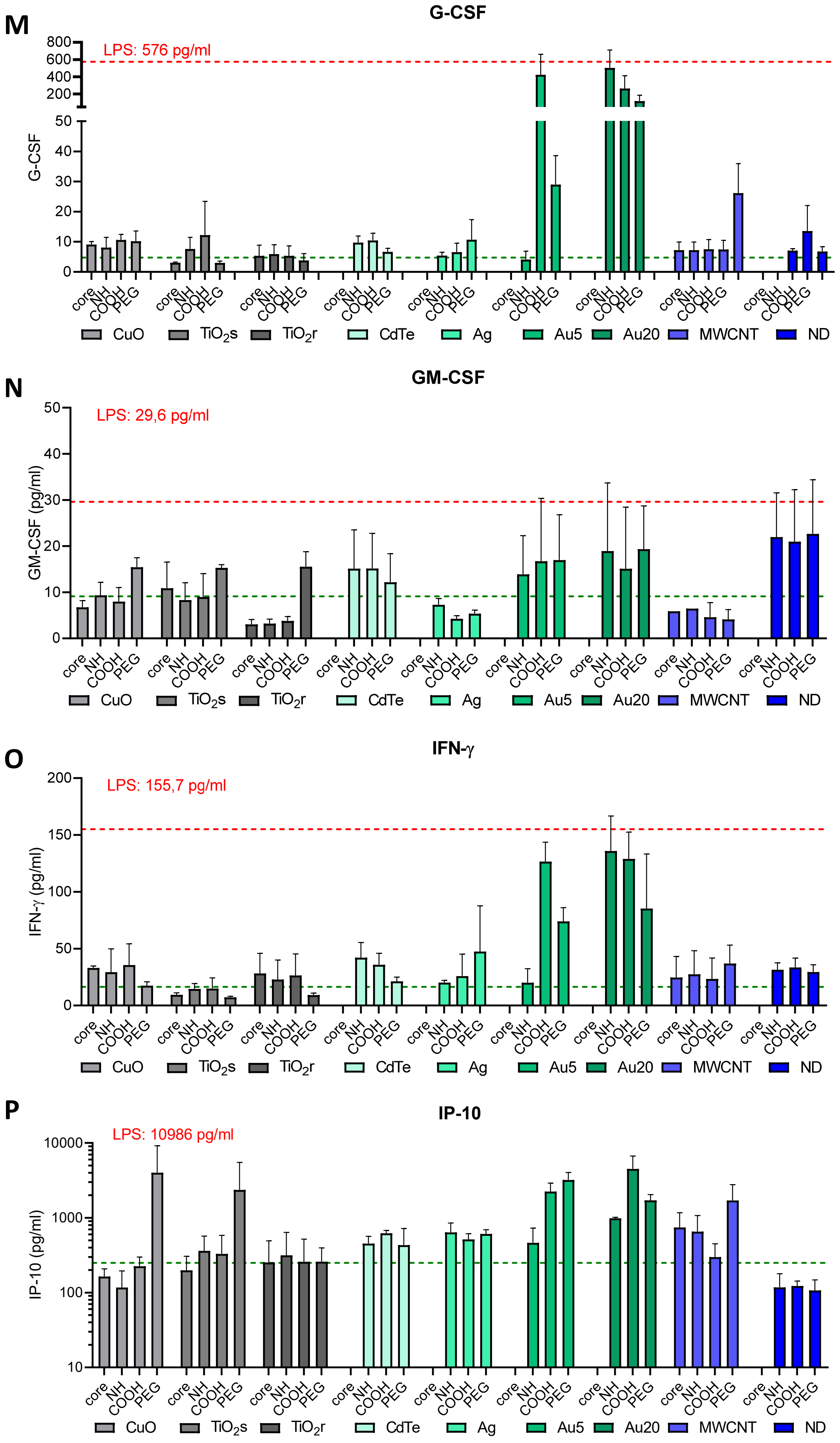
M
G-CSF
LPS: 576 pg/ml
G-CSF
N
GM-CSF
LPS: 29,6 pg/ml
GM-CSF (pg/ml)
O
IFN-γ
LPS: 155,7 pg/ml
IFN-γ (pg/ml)
P
IP-10
LPS: 10986 pg/ml
IP-10 (pg/ml)
core NH COOH PEG
CuO TiO2s TiO2r CdTe Ag Au5 Au20 MWCNT ND

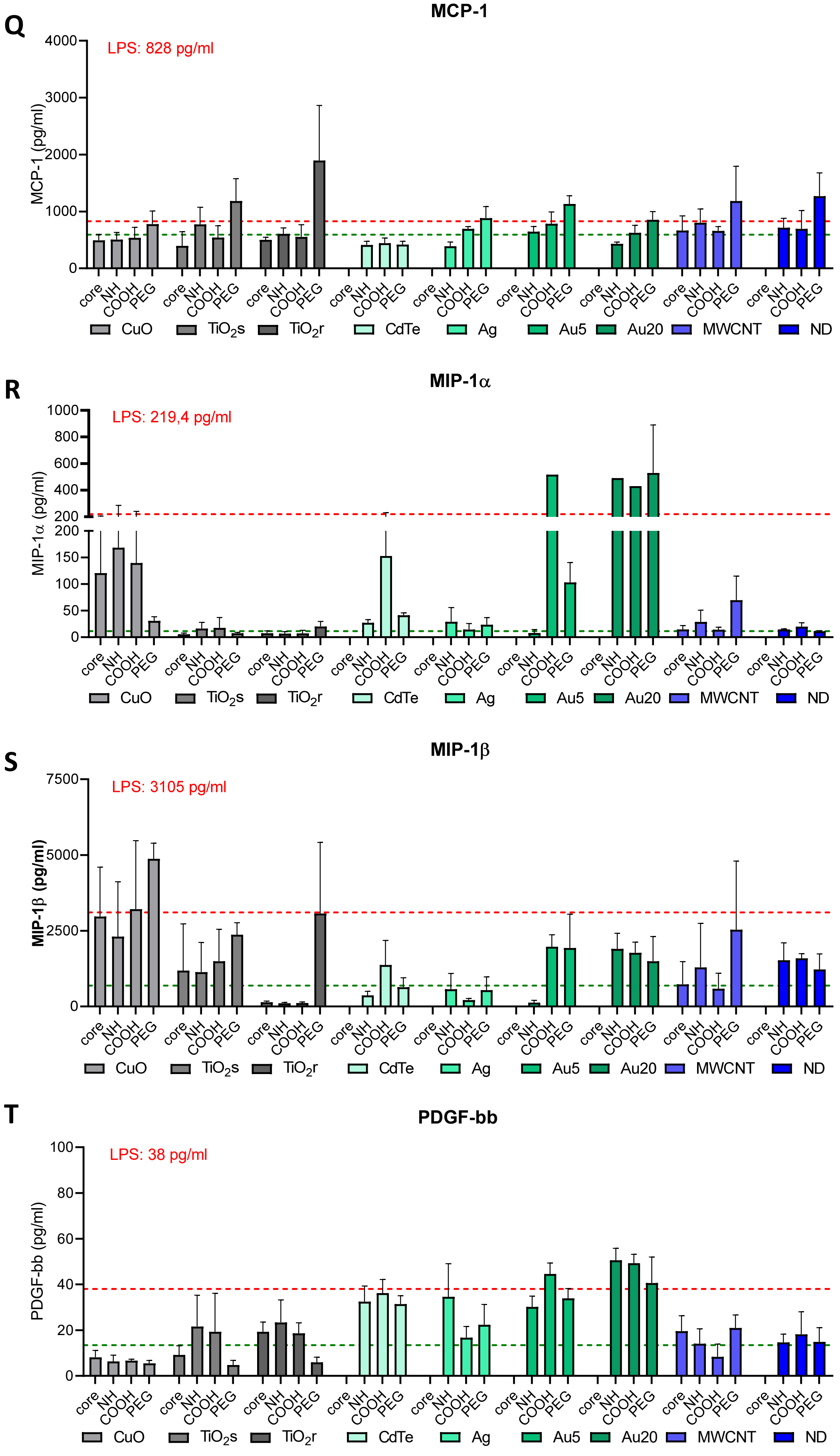
Q
MCP-1
LPS: 828 pg/ml
MCP-1 (pg/ml)
4000
3000
2000
1000
0
core
NH
COOH
PEG
CuO
TiO2s
TiO2r
CdTe
Ag
Au5
Au20
MWCNT
ND
R
MIP-1α
LPS: 219,4 pg/ml
MIP-1α (pg/ml)
1000
800
600
400
200
150
100
50
S
MIP-1β
LPS: 3105 pg/ml
MIP-1β (pg/ml)
7500
5000
2500
T
PDGF-bb
LPS: 38 pg/ml
PDGF-bb (pg/ml)
100
80
60
40
20

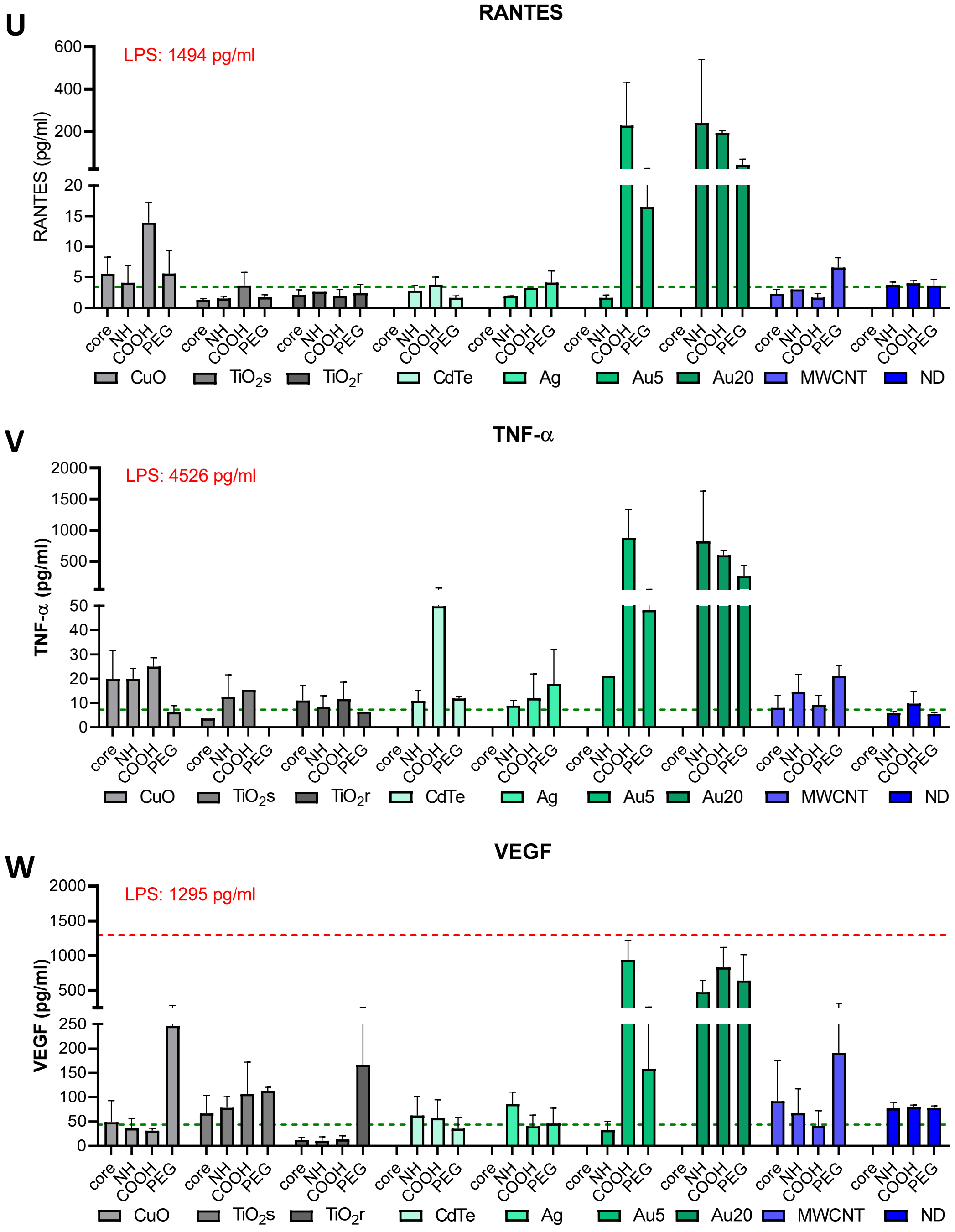

Figure S5. Cytokine release by primary human monocyte-derived macrophages (HMDMs) exposed to 31 ENMs. Cells were exposed to ENMs for 24 h at doses inducing a maximum of 10-15% of cell death (EC_{10}) and secretion of cytokines, chemokines. and growth factors was monitored by using the BioPlex Pro™ Human Cytokine Standard 27-Plex array. LPS (0,1 µg/mL) was included as a positive control. Data shown in (A) to (W) are mean values ± S.D. (n=3).

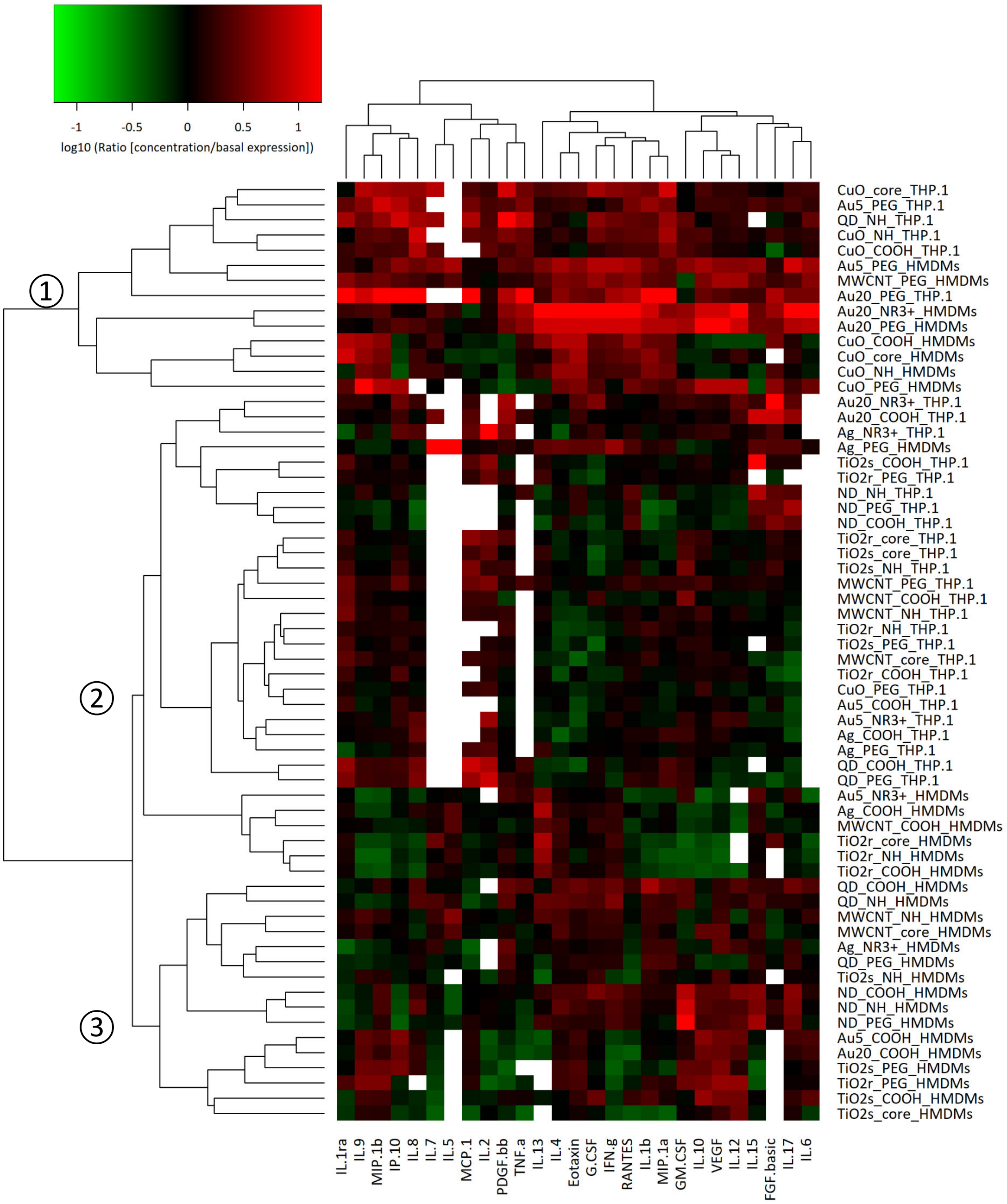

Figure S6. Cytokine profiling of primary human monocyte-derived macrophages (HMDMs) and the human monocytic THP-1 cell line exposed to 31 EMNs. The cells were exposed to ENM doses inducing a maximum of 10-15% of cell death (EC_{10}) and the secretion of cytokines, chemokines, and growth factors into the supernatant was monitored by using the BioPlex Pro™ Human Cytokine Standard 27-Plex array. Data are normalized mean values expressed by the log10 of the concentration of proteins released after ENM exposure divided by their basal expression (medium alone). The hierarchical clustering revealed a grouping of samples into three clusters: (1) the most inflammogenic ENMs, (2) the THP-1 responses (mainly), and (3) the HMDM responses (mainly).

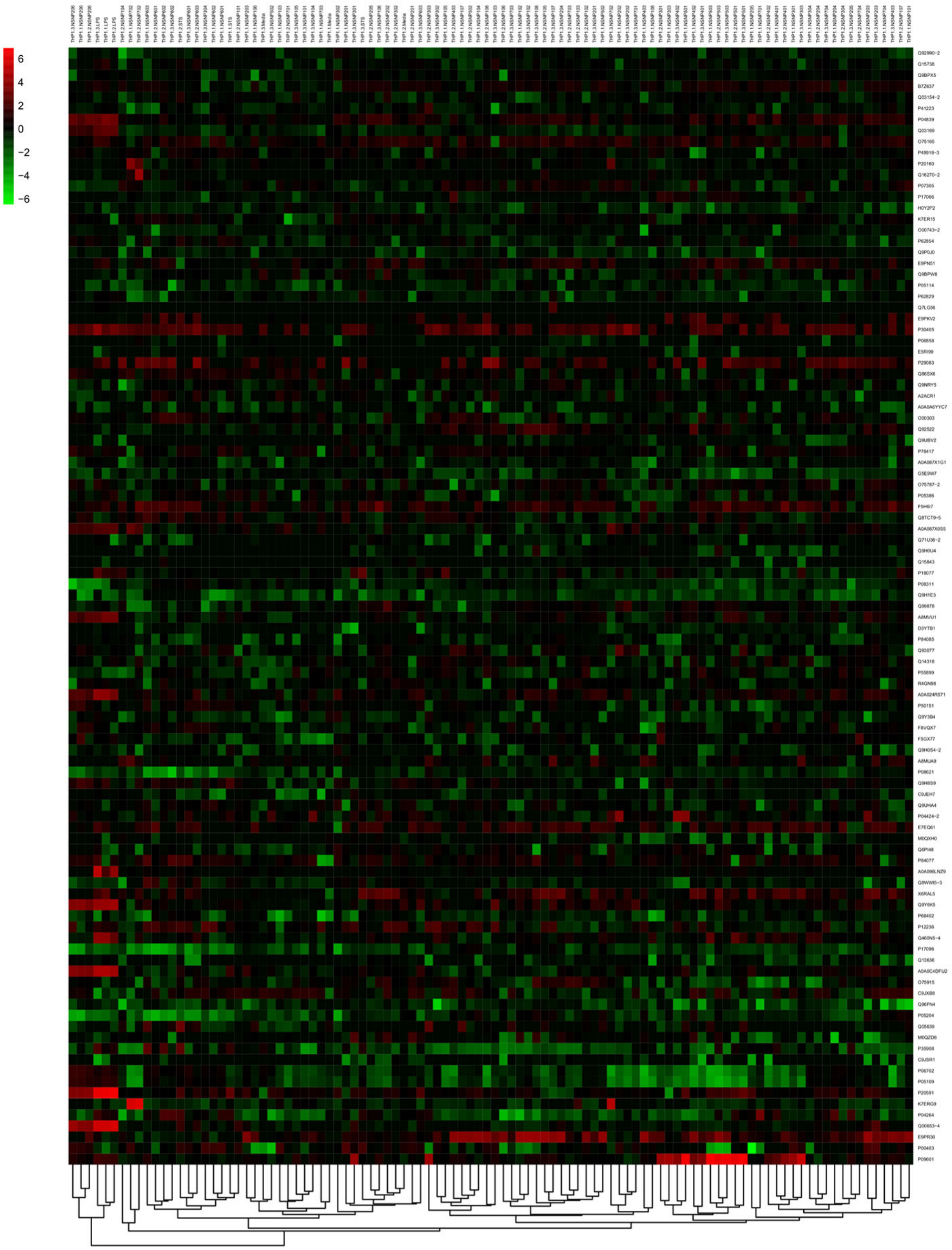

Figure S7. Proteomics profiling of THP-1 cells exposed to 31 ENMs. Protein expression heatmap depicting the top 100 proteins dysregulated in THP-1 cells upon ENM exposure for 24 h at doses inducing a maximum of 10-15% of cell death (EC_{10}). Hierarchical clustering analysis provided no evidence for grouping of the ENMs.

A

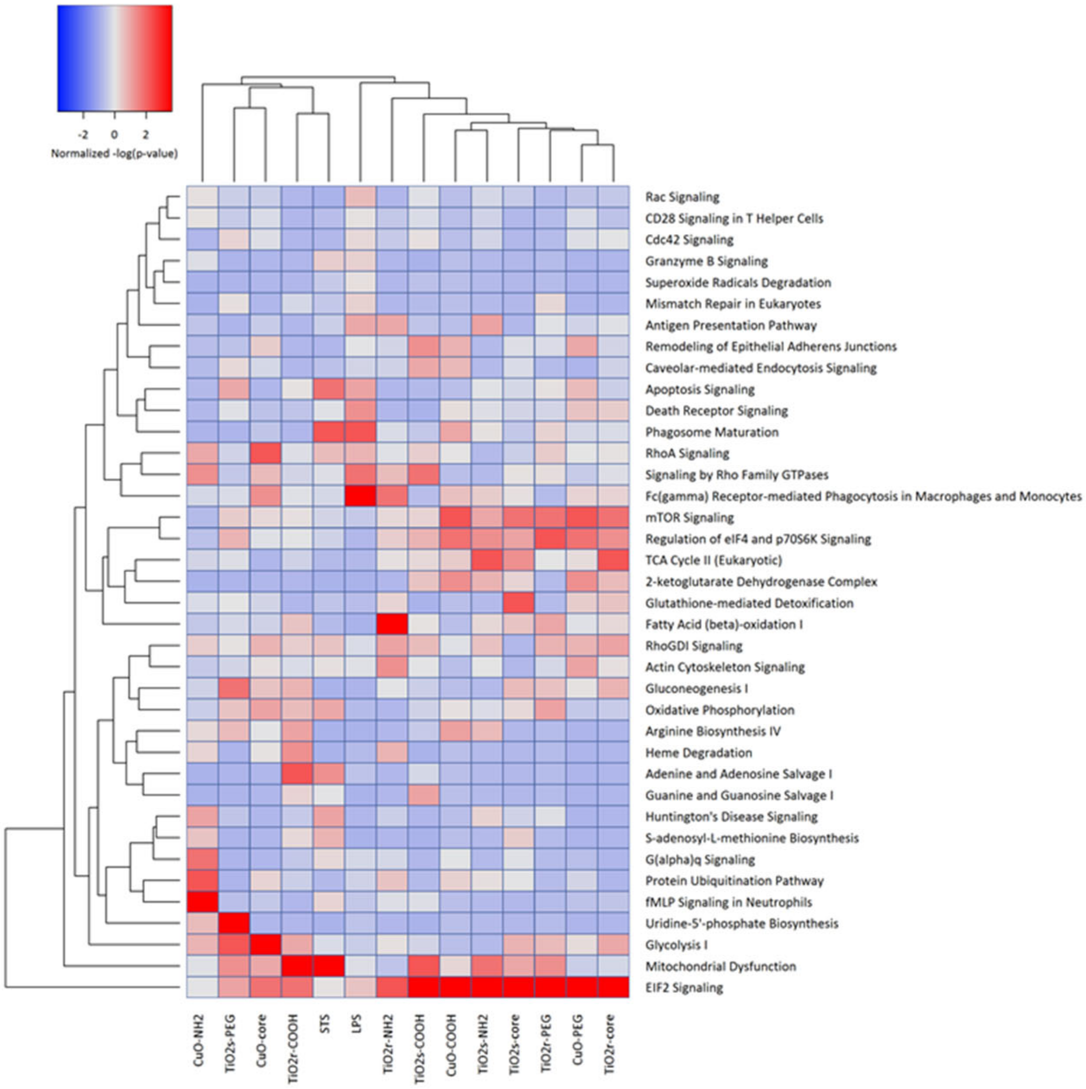

Figure S8. Pathway analysis of the proteomics data. (A). Pathway analysis of the metal-oxide based ENMs allowing the visualization of the activation (red) or inactivation (blue) of the most significant canonical pathways associated across the multiple conditions. The significance values for the canonical pathways were calculated by Fisher's exact test right-tailed and indicates the probability of association of the proteins with the respective pathway. Score cutoff for the p-value is $p<0.001$ for at least one of the conditions.

B

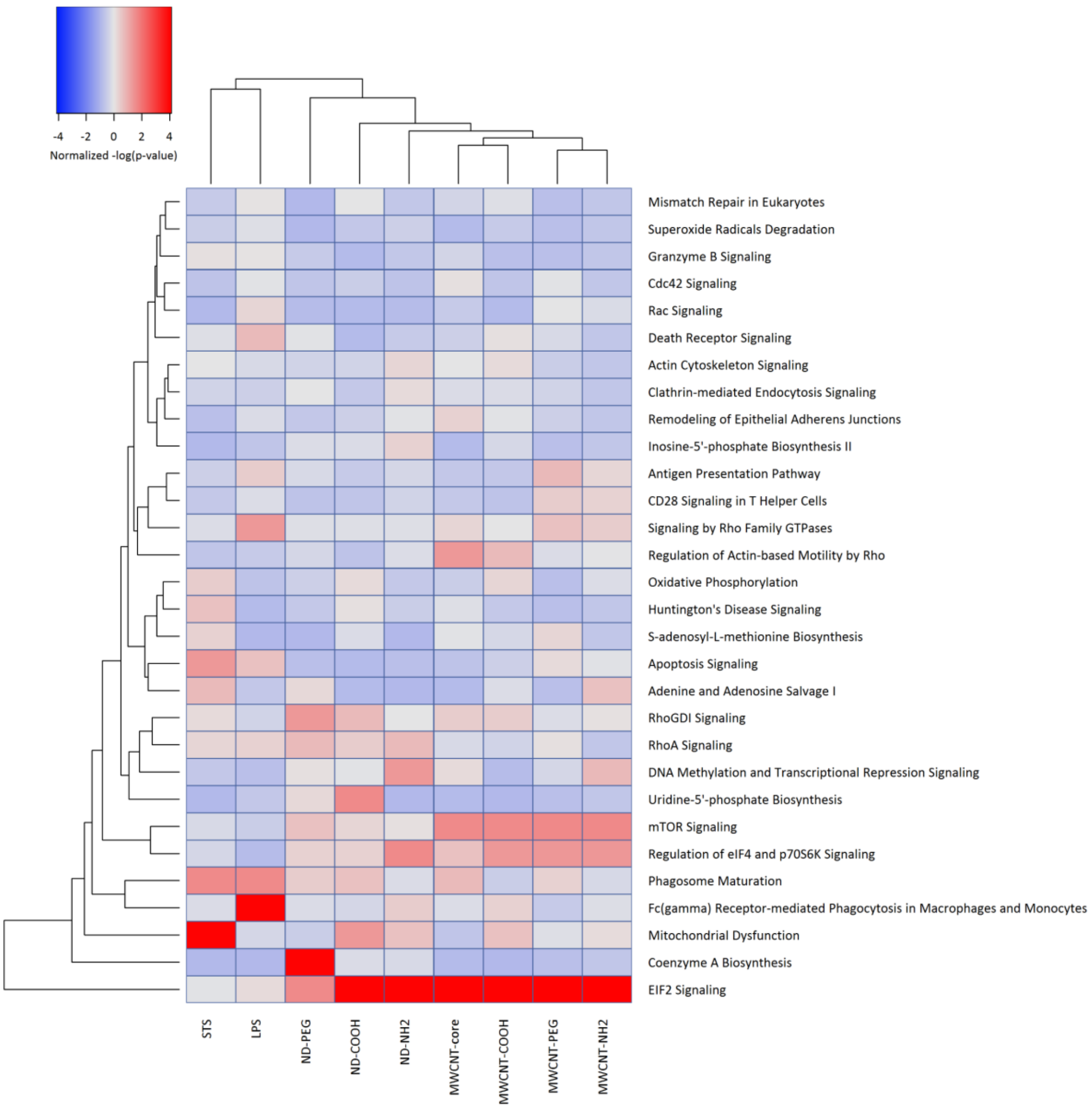

Figure S8. Pathway analysis of the proteomics data. (B). Pathway analysis of the carbon-based ENMs allowing the visualization of the activation (red) or inactivation (blue) of the most significant canonical pathways associated across the multiple conditions. The significance values for the canonical pathways were calculated by Fisher's exact test right-tailed and indicates the probability of association of the proteins with the respective pathway. Score cutoff for the p-value is $p<0.001$ for at least one of the conditions.

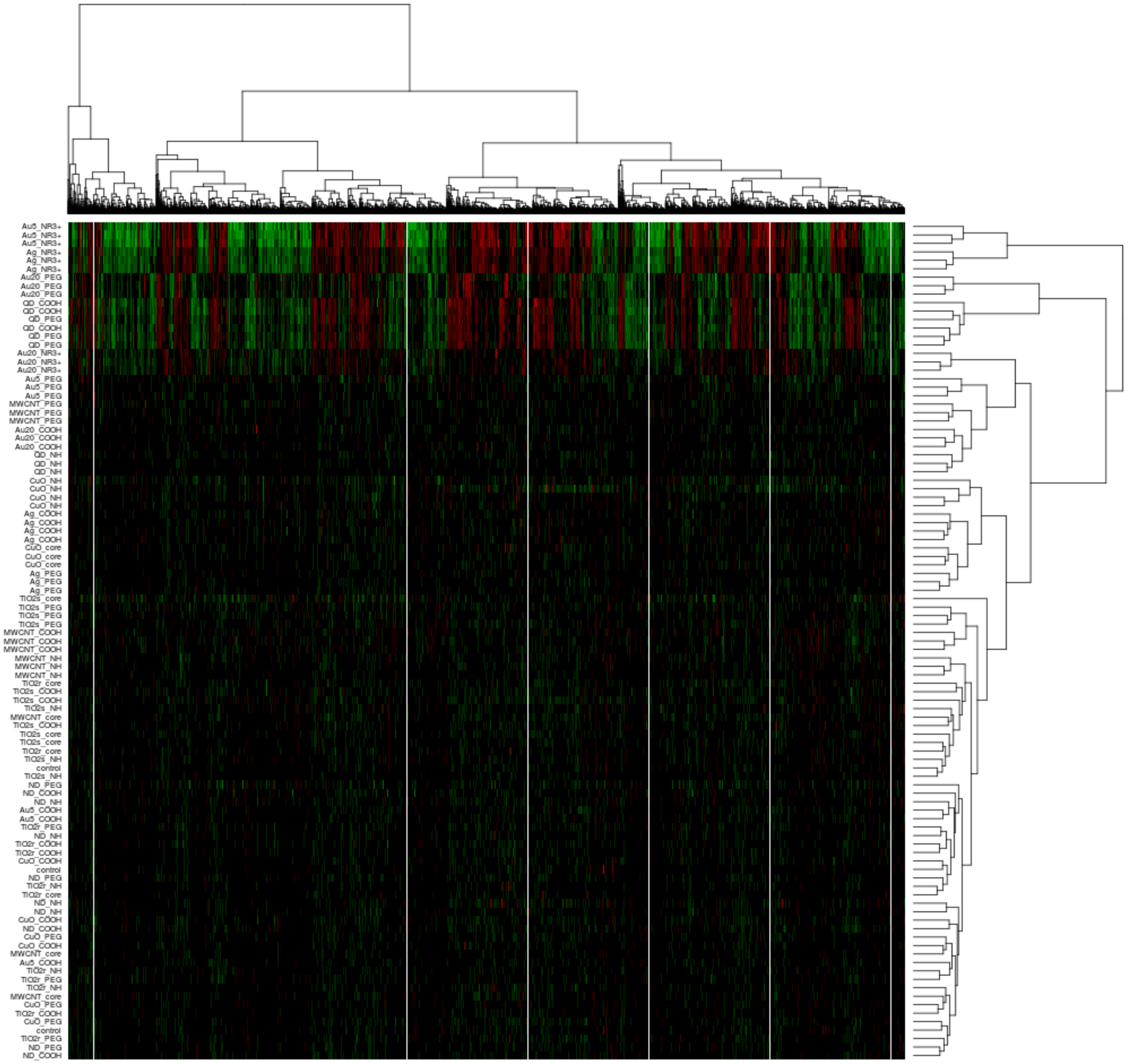

Figure S9. Natural clustering of the microarray data before averaging of the three replicates. Hierarchical clustering of DEGs significantly regulated in THP-1 cells upon exposure for 24 h to the 31 ENMs at EC_{10} doses. The heatmap was colored based on z-scores of normalized expression levels.

UP-REGULATED			DOWN-REGULATED		
Ag-NR3+ (2004 significant DEGs)	*Adjusted P-value*		*Ag-NR3+ (1802 significant DEGs)*	*Adjusted P-value*	
cytokine-mediated signaling pathway (GO:0019221)	1,59E-07	***	DNA metabolic process (GO:0006259)	3,24E-34	***
cellular response to cadmium ion (GO:0071276)	4,25E-07	***	DNA replication (GO:0006260)	9,86E-25	***
interferon-gamma-mediated signaling pathway (GO:0060333)	4,25E-07	***	G1/S transition of mitotic cell cycle (GO:0000082)	4,89E-17	***
response to cadmium ion (GO:0046686)	1,14E-06	***	DNA repair (GO:0006281)	1,94E-13	***
cellular response to copper ion (GO:0071280)	1,14E-06	***	cell cycle G1/S phase transition (GO:0044843)	7,88E-13	***
response to copper ion (GO:0046688)	1,46E-06	***	DNA-dependent DNA replication (GO:0006261)	2,84E-12	***
response to zinc ion (GO:0010043)	1,46E-06	***	DNA biosynthetic process (GO:0071897)	6,66E-12	***
cellular response to zinc ion (GO:0071294)	1,92E-06	***	mitotic cell cycle phase transition (GO:0044772)	6,66E-12	***
lipopolysaccharide-mediated signaling pathway (GO:0031663)	2,74E-05	***	DNA recombination (GO:0006310)	1,05E-11	***
neutrophil degranulation (GO:0043312)	2,74E-05	***	cellular response to DNA damage stimulus (GO:0006974)	3,96E-11	***
Au-5-NR3+ (2625 significant DEGs)	*Adjusted P-value*		*Au-5-NR3+ (2682 significant DEGs)*	*Adjusted P-value*	
protein transport (GO:0015031)	8,34E-04	***	DNA metabolic process (GO:0006259)	5,75E-37	***
cellular protein localization (GO:0034613)	8,34E-04	***	DNA replication (GO:0006260)	1,13E-22	***
vesicle-mediated transport (GO:0016192)	4,83E-03	**	DNA repair (GO:0006281)	2,97E-17	***
regulation of MAP kinase activity (GO:0043405)	1,46E-02	*	DNA recombination (GO:0006310)	1,76E-12	***
intraciliary transport involved in cilium assembly (GO:0035735)	1,58E-02	*	G1/S transition of mitotic cell cycle (GO:0000082)	4,89E-12	***
positive regulation of hydrolase activity (GO:0051345)	1,58E-02	*	RNA metabolic process (GO:0016070)	1,71E-11	***
I-kappaB kinase/NF-kappaB signaling (GO:0007249)	1,58E-02	*	DNA biosynthetic process (GO:0071897)	3,35E-11	***
regulation of cell migration (GO:0030334)	1,58E-02	*	ribosome biogenesis (GO:0042254)	3,35E-11	***
positive regulation of GTPase activity (GO:0043547)	1,64E-02	*	mRNA splicing, via spliceosome (GO:0000398)	5,21E-11	***
intraciliary transport (GO:0042073)	2,27E-02	*	DNA-dependent DNA replication (GO:0006261)	9,35E-11	***
Au-20-NR3+ (852 significant DEGs)	*Adjusted p-value*		*Au-20-NR3+ (407 significant DEGs)*	*Adjusted p-value*	
cytokine-mediated signaling pathway (GO:0019221)	2,63E-12	***	DNA metabolic process (GO:0006259)	9,02E-40	***
interferon-gamma-mediated signaling pathway (GO:0060333)	1,32E-08	***	DNA replication (GO:0006260)	1,33E-37	***
cellular response to interferon-gamma (GO:0071346)	1,16E-07	***	G1/S transition of mitotic cell cycle (GO:0000082)	1,71E-27	***
inflammatory response (GO:0006954)	1,54E-07	***	cell cycle G1/S phase transition (GO:0044843)	4,15E-24	***
neutrophil degranulation (GO:0043312)	1,14E-06	***	DNA-dependent DNA replication (GO:0006261)	2,04E-20	***
neutrophil activation involved in immune response (GO:0002283)	1,26E-06	***	mitotic cell cycle phase transition (GO:0044772)	9,41E-20	***
neutrophil mediated immunity (GO:0002446)	1,43E-06	***	cellular response to DNA damage stimulus (GO:0006974)	3,70E-19	***
cellular response to lipopolysaccharide (GO:0071222)	3,32E-06	***	cellular macromolecule biosynthetic process (GO:0034645)	4,19E-19	***
lipopolysaccharide-mediated signaling pathway (GO:0031663)	5,91E-05	***	DNA repair (GO:0006281)	8,30E-18	***
cellular response to cytokine stimulus (GO:0071345)	1,25E-04	***	DNA replication initiation (GO:0006270)	2,83E-12	***
Au-20-PEG (1153 significant DEGs)	*Adjusted p-value*		*Au-20-PEG (1127 significant DEGs)*	*Adjusted p-value*	
cytokine-mediated signaling pathway (GO:0019221)	4,83E-43	***	carnitine transport (GO:0015879)	5,75E-01	*ns*
inflammatory response (GO:0006954)	3,67E-21	***	amino-acid betaine transport (GO:0015838)	5,75E-01	*ns*
type I interferon signaling pathway (GO:0060337)	3,07E-17	***	pyrimidine deoxyribonucleotide catabolic process (GO:0009223)	5,75E-01	*ns*
cellular response to type I interferon (GO:0071357)	3,07E-17	***	acyl-CoA biosynthetic process (GO:0071616)	7,76E-01	*ns*
neutrophil mediated immunity (GO:0002446)	7,24E-17	***	ganglion development (GO:0061548)	9,34E-01	*ns*
neutrophil degranulation (GO:0043312)	7,51E-17	***	neutrophil degranulation (GO:0043312)	9,34E-01	*ns*
neutrophil activation involved in immune response (GO:0002283)	1,10E-16	***	neutrophil mediated immunity (GO:0002446)	9,34E-01	*ns*
cellular response to interferon-gamma (GO:0071346)	1,84E-16	***	neutrophil activation involved in immune response (GO:0002283)	9,34E-01	*ns*
interferon-gamma-mediated signaling pathway (GO:0060333)	1,84E-16	***	carnitine metabolic process (GO:0009437)	9,71E-01	*ns*
cellular response to cytokine stimulus (GO:0071345)	3,61E-16	***	acetyl-CoA biosynthetic process (GO:0006085)	9,71E-01	*ns*
QD-PEG (1623 significant DEGs)	*Adjusted p-value*		*QD-PEG (1381 significant DEGs)*	*Adjusted p-value*	
cytokine-mediated signaling pathway (GO:0019221)	1,05E-26	***	mitotic sister chromatid segregation (GO:0000070)	2,29E-04	***
inflammatory response (GO:0006954)	1,96E-13	***	regulation of cell cycle G2/M phase transition (GO:1902749)	2,29E-04	***
neutrophil degranulation (GO:0043312)	6,14E-13	***	mitotic cell cycle phase transition (GO:0044772)	2,29E-04	***
neutrophil mediated immunity (GO:0002446)	6,14E-13	***	cell cycle G2/M phase transition (GO:0044839)	1,21E-03	**
neutrophil activation involved in immune response (GO:0002283)	8,19E-13	***	G2/M transition of mitotic cell cycle (GO:0000086)	1,21E-03	**
type I interferon signaling pathway (GO:0060337)	2,38E-12	***	regulation of mitotic cell cycle phase transition (GO:1901990)	5,46E-03	**
cellular response to type I interferon (GO:0071357)	2,38E-12	***	centromere complex assembly (GO:0034508)	1,18E-02	*
interferon-gamma-mediated signaling pathway (GO:0060333)	2,58E-12	***	microtubule cytoskeleton organization involved in mitosis (GO:1902850)	1,20E-02	*
cellular response to interferon-gamma (GO:0071346)	2,65E-12	***	metaphase plate congression (GO:0051310)	1,20E-02	*
cellular response to cytokine stimulus (GO:0071345)	1,63E-11	***	ciliary basal body-plasma membrane docking (GO:0097711)	1,20E-02	*
QD-COOH (1716 significant DEGs)	*Adjusted p-value*		*QD-COOH (1514 significant DEGs)*	*Adjusted p-value*	
cytokine-mediated signaling pathway (GO:0019221)	6,52E-28	***	centromere complex assembly (GO:0034508)	7,39E-04	***
type I interferon signaling pathway (GO:0060337)	2,90E-13	***	chromatin remodeling at centromere (GO:0031055)	2,98E-03	**
cellular response to type I interferon (GO:0071357)	2,90E-13	***	DNA replication-independent nucleosome assembly (GO:0006336)	2,98E-03	**
cellular response to cytokine stimulus (GO:0071345)	7,81E-13	***	mitotic sister chromatid segregation (GO:0000070)	2,98E-03	**
inflammatory response (GO:0006954)	1,18E-12	***	mitotic cell cycle phase transition (GO:0044772)	2,98E-03	**
neutrophil degranulation (GO:0043312)	1,53E-12	***	CENP-A containing nucleosome assembly (GO:0034080)	4,39E-03	**
neutrophil mediated immunity (GO:0002446)	1,53E-12	***	CENP-A containing chromatin organization (GO:0061641)	4,39E-03	**
neutrophil activation involved in immune response (GO:0002283)	2,15E-12	***	histone exchange (GO:0043486)	4,39E-03	**
cellular response to interferon-gamma (GO:0071346)	7,32E-11	***	sister chromatid segregation (GO:0000819)	6,79E-03	**
negative regulation of viral life cycle (GO:1903901)	7,32E-11	***	DNA metabolic process (GO:0006259)	9,32E-03	**

Table S2. Gene ontology (GO) enrichment analysis. Top-10 biological process GO terms for Ag-NR3+, Au-5-NR3+, Au-20-NR3+, Au-20-PEG, QD-PEG, and QD-COOH ENMs sorted by adjusted p-value. GO terms are shown according to up- or downregulated DEGs as determined by RNA-seq with their corresponding adjusted p-values. The p-values were computed from Fisher's exact test with Bonferroni correction: * $p < 0.05$, ** $p < 0.01$, *** $p < 0.001$.

Au-5-NR3+			Ag-NR3+			Au-20-PEG			QD-PEG			QD-COOH			Au-20-NR3+			
Gene Symbol	log FC	adj. P-value	Gene Symbol	log FC	adj. P-value	Gene Symbol	log FC	adj. P-value	Gene Symbol	log FC	adj. P-value	Gene Symbol	log FC	adj. P-value	Gene Symbol	log FC	adj. P-value	
GDF15	5,619313623	8,865E-42	MT1G	5,937036004	7,8742E-53	IGFBP3	7,400315781	6,7348E-55	HMOX1	8,604686709	7,1886E-21	HMOX1	8,596321733	6,6828E-21	LY96	2,734806594	3,7046E-25	Top 20 up-regulated
DACT3	5,057998438	5,8428E-53	MT1X	5,609690793	4,3412E-42	EBI3	7,38307401	1,9686E-50	MT1G	7,024173014	7,3579E-54	MT1G	7,027061653	4,7643E-54	IL4I1	2,732182745	5,0537E-22	
KLF2	4,715574287	1,8897E-50	MT1H	5,467189208	4,3693E-48	CXCL14	7,000383155	6,7348E-55	MT1H	6,592816943	3,0922E-50	IL4I1	6,813553182	2,0978E-47	LOC284561	2,589217316	3,8579E-33	
HMOX1	4,388407254	2,9485E-09	MT1M	4,916576793	5,5148E-41	IL1B	6,623479866	4,6421E-51	IGFBP3	6,56496079	2,1477E-51	IGFBP3	6,780317298	2,2032E-52	HLA-DMB	2,169193324	1,7688E-26	
HLA-DMB	4,183116808	2,748E-45	MT2A	4,70505861	6,7575E-11	IL4I1	6,38912568	1,4865E-45	IL4I1	6,557145019	3,1628E-46	MT1H	6,724115986	6,8698E-51	GPR68	2,138260078	1,2018E-24	
LY96	3,996049435	2,2346E-36	IL4I1	4,636020056	4,2616E-39	MMP9	6,05960485	7,1564E-47	MT1X	6,456627442	2,3701E-43	MT1X	6,604504945	4,8573E-44	SLC7A11	2,044894246	1,3529E-20	
PPM1J	3,975395937	3,2463E-43	MT1L	4,601712793	6,3456E-21	CCL3	6,049818746	2,3973E-42	CCL3	6,014147697	3,9489E-42	MX1	6,130643238	1,9143E-47	CCL2	2,038302493	8,4237E-27	
EGR2	3,837462101	2,4971E-36	MT1B	4,593081099	1,2819E-24	LAMP3	5,868844685	5,6136E-53	MX1	5,972099582	1,3941E-46	CCL3	6,123202714	1,0759E-42	COL4A4	2,026288765	1,2018E-24	
LAMP3	3,819185267	7,328E-41	HMOX1	4,567189321	1,2325E-10	SLAMF7	5,671090455	1,1749E-56	MT1M	5,902005525	1,8304E-43	MT1M	5,953241095	9,0156E-44	ALOX5	2,012111024	3,2184E-31	
RGS2	3,748492794	2,748E-45	MT1HL1	4,390518257	2,6029E-24	CCL2	5,647688876	3,7942E-56	IFIT1	5,820426521	4,258E-47	IFIT1	5,946942357	8,3966E-48	CYP1B1	1,996655637	3,1484E-32	
RGS16	3,747625261	1,9186E-35	GDF15	4,255989564	1,2113E-36	CCL4L2	5,557143687	3,1288E-48	GDF15	5,505435471	2,6592E-41	MAFB	5,751711789	3,1004E-54	EBI3	1,976131324	5,5671E-16	
SLAMF7	3,723672331	6,7856E-45	MT1A	4,234698823	4,6245E-19	CCL3L3	5,462686948	2,0521E-48	MAFB	5,495245398	5,2345E-53	SGK1	5,594010929	2,8916E-55	ZFP36L1	1,95751394	3,8701E-27	
IL1B	3,676598148	7,8865E-35	MT1E	4,132311118	9,8363E-27	CXCL1	5,391479967	3,0642E-49	CCL3L3	5,442551074	3,9839E-48	GDF15	5,577796239	9,4044E-42	IFI16	1,892634674	9,5459E-16	
ZFP36L1	3,586966873	6,7856E-45	MT1F	4,087896023	4,9054E-43	MX1	5,371425843	1,0009E-43	IFI44	5,320086193	3,0998E-43	IFI44	5,532031455	2,3117E-44	SDS	1,883780502	2,409E-19	
SPINK2	3,470803041	8,8245E-35	CD86	3,988222068	4,5193E-07	IL7R	5,250124804	2,4345E-39	SGK1	5,313654318	7,3579E-54	CCL3L3	5,505491317	1,5291E-48	FEZ1	1,881777968	1,7688E-26	
TIPARP	3,468216664	3,1169E-39	LY96	3,944115882	1,8673E-38	SGK1	5,1220793	3,002E-53	IFI44L	5,206848116	1,8313E-48	IFI44L	5,430429019	8,155E-50	SULF2	1,865584085	3,8916E-13	
SLC7A11	3,45518072	9,8631E-35	lnc-SOX6-1	3,891222542	6,539E-07	ICAM1	5,09924073	7,0378E-50	TDO2	5,055180691	3,7545E-41	IFIT3	5,156221592	1,3338E-50	ICAM1	1,848529472	8,6609E-22	
SDS	3,412625137	4,8696E-35	HSPA6	3,741679136	7,4245E-36	MAFB	5,054094198	6,4033E-51	SLAMF8	5,029177783	1,6383E-45	TDO2	5,14498151	9,7781E-42	GDF15	1,821829649	5,8824E-14	
TRIB1	3,394385921	2,1399E-40	lnc-RPP30-2	3,719350915	7,7893E-07	CD14	4,994480892	6,3804E-50	IFIT3	4,977023062	1,5985E-49	LOC344887	5,106871136	5,4322E-46	BCL3	1,800600745	5,0368E-18	
KIAA0513	3,346121135	1,5138E-38	HLA-DMB	3,634645739	1,6284E-44	MMP1	4,942380071	5,1208E-51	MT1B	4,930847967	1,6597E-24	SLAMF8	5,093465694	5,6254E-46	IL1B	1,800036655	9,5556E-17	
S100A8	-4,80144748	1,3799E-38	SERPINB2	-3,73745482	1,1669E-37	MS4A3	-4,25656676	2,4114E-40	MS4A3	-4,26398587	2,3419E-40	MS4A3	-4,44560413	1,3323E-41	DEFA4	-2,30200571	3,8701E-27	Top 20 down-regulated
SERPINB2	-4,7769166	1,2746E-44	S100A8	-3,27636351	2,6688E-28	SERPINB2	-3,98756426	6,4607E-40	SERPINB2	-3,80361719	1,3346E-38	SERPINB2	-4,4352259	6,994E-43	LOC101929612	-1,80101165	4,6813E-18	
PPP1R27	-4,22229185	1,1756E-42	MS4A3	-3,27467551	7,3371E-33	GPA33	-3,96400501	2,1455E-46	lnc-CIB3-1	-3,77490805	1,6683E-40	lnc-CIB3-1	-4,02361169	2,3651E-42	SPP1	-1,64472799	7,9589E-16	
lnc-CIB3-1	-3,90218839	2,5101E-41	PRTN3	-3,11392207	2,1519E-26	CTSG	-3,69791365	1,0836E-39	MLC1	-3,39033399	6,509E-44	MLC1	-3,79707249	3,2786E-47	LOC100131262	-1,63366356	1,8914E-23	
PRTN3	-3,87017502	3,2582E-32	lnc-CIB3-1	-2,94258409	2,0073E-33	CEACAM6	-3,64577141	4,7812E-43	CTSG	-3,02673719	3,1516E-34	PRTN3	-3,46631434	2,5543E-29	PRTN3	-1,5587897	1,3062E-11	
CXorf21	-3,63815374	1,1997E-32	UNG	-2,85170485	6,1909E-39	DEFB1	-3,60713414	4,6242E-43	PRTN3	-2,99167505	1,4869E-25	STAR	-3,31708557	5,0342E-48	FAM222A	-1,49983643	1,3637E-20	
MS4A6A	-3,53551403	1,5005E-44	DEFA4	-2,84355529	4,6569E-34	STAR	-3,52741896	8,3889E-50	STAR	-2,96876738	6,6106E-45	GPA33	-3,13290267	7,0436E-40	ARRDC4	-1,48230665	2,2483E-17	
MLC1	-3,45520223	3,138E-44	HP	-2,83846879	1,8995E-33	lnc-CIB3-1	-3,46197165	3,519E-38	GPA33	-2,8717597	2,0453E-37	CTSG	-3,11957901	4,1836E-35	MANF	-1,45355274	1,8011E-16	
CPNE7	-3,40714838	1,2393E-39	GAL	-2,74400984	2,8004E-35	PPP1R27	-3,37148398	1,249E-36	CACNA2D3	-2,82468312	2,591E-38	CACNA2D3	-3,054623	1,6105E-40	NDOR1	-1,43529099	1,889E-14	
GAL	-3,36932685	3,871E-41	AGMAT	-2,61612914	2,8332E-44	LINC00977	-3,25008659	1,7695E-41	DEFA4	-2,72806559	2,3661E-33	C1orf228	-2,92119407	5,8831E-41	GINS2	-1,41691006	2,4162E-25	
HPDL	-3,31616093	1,7864E-39	CPNE7	-2,60667239	3,796E-32	PRTN3	-3,18495479	4,5245E-27	C1orf228	-2,62565494	5,7903E-38	lnc-PPIE-1	-2,90789292	8,1882E-32	SERPINI2	-1,38424278	1,5663E-08	
S100A9	-3,30874651	7,082E-34	GINS2	-2,54805602	1,0226E-41	FBP1	-3,17273735	8,8237E-47	lnc-PPIE-1	-2,58077459	1,1834E-28	CEACAM6	-2,797716	8,0304E-36	MS4A3	-1,35124934	1,2813E-12	
UNG	-3,25068405	6,4279E-43	CTSG	-2,54217382	2,2765E-29	lnc-PPIE-1	-2,913539	8,5664E-32	CEACAM6	-2,57398218	1,6338E-33	CASC10	-2,7527579	2,2301E-31	TERT	-1,34295911	5,2224E-15	
AGMAT	-3,23796102	1,4541E-50	HPDL	-2,51435233	8,953E-32	MLC1	-2,91032044	1,0385E-39	CASC10	-2,56414236	1,7348E-29	COL9A3	-2,73516613	9,4044E-42	SNORD86	-1,29711789	6,0072E-15	
CDC25A	-3,16262054	3,4647E-40	CXorf21	-2,47554404	7,7372E-23	SEL1L3	-2,8952619	1,66E-38	LAMP5	-2,52602782	6,7096E-39	DEFA4	-2,72988895	2,0043E-33	MLC1	-1,29660671	2,0054E-18	
CACNA2D3	-3,12662383	5,4464E-41	MLC1	-2,46404116	8,9117E-35	ERVH-3	-2,88493488	8,3584E-31	SERPINI2	-2,52171064	3,1142E-19	LAMP5	-2,65026973	2,6822E-40	UHRF1	-1,28695705	5,6424E-21	
HP	-3,12283262	2,3955E-36	AVEN	-2,45126735	5,2502E-44	SERPINI2	-2,82709101	1,0537E-21	LOC100506844	-2,46089111	2,7156E-42	LOC100506844	-2,64316599	2,3849E-44	GAL	-1,28107127	1,9798E-16	
LPPR3	-3,11978206	1,068E-25	MS4A6A	-2,39847613	8,3294E-34	LOC100996338	-2,8140498	3,5106E-33	COL9A3	-2,4439789	1,2995E-38	LPPR3	-2,60232398	2,7164E-21	UNG	-1,26436552	3,6613E-18	
MS4A3	-3,04587389	2,1259E-31	HPR	-2,3953567	6,8729E-34	LOC102723894	-2,78399743	1,7862E-38	CAV1	-2,34977554	3,4889E-27	CAV1	-2,51706882	5,3793E-29	CDC25A	-1,26315068	1,1473E-16	
MCM4	-3,03240599	2,748E-45	VIT	-2,39157704	6,0668E-31	DEFA4	-2,76369316	1,1031E-33	SERPINB10	-2,32183824	6,5774E-34	SERPINI2	-2,48044188	6,0846E-19	E2F1	-1,25371688	2,2645E-23	

Table S3. Top 20 up- and downregulated DEGs in cells exposed to the indicated ENMs. The data were filtered using the following criteria: logFC ≥ 0.58 and adjusted p-values < 10^{-5}. Log FC values and adjusted p-values are shown.

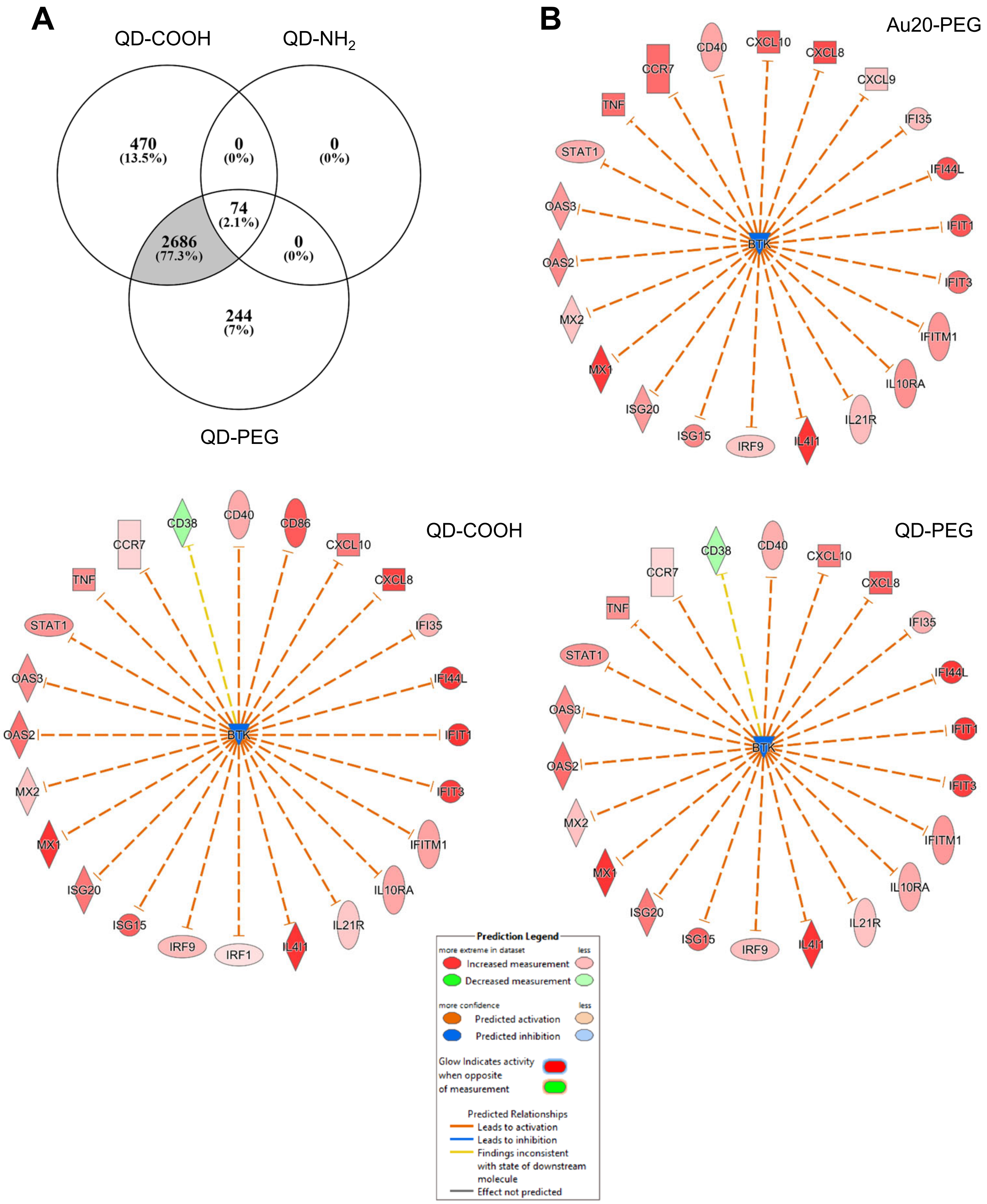

Figure S10. (A) Venn diagram depicting DEGs (FC > 1.5 and adjusted p-value < 0.05) in THP-1 cells exposed to QDs. (B) Upstream regulator analysis of the transcriptomics results identified Bruton's tyrosine kinase (*BTK*) as a putative regulator with a negative activation score for the indicated ENMs.

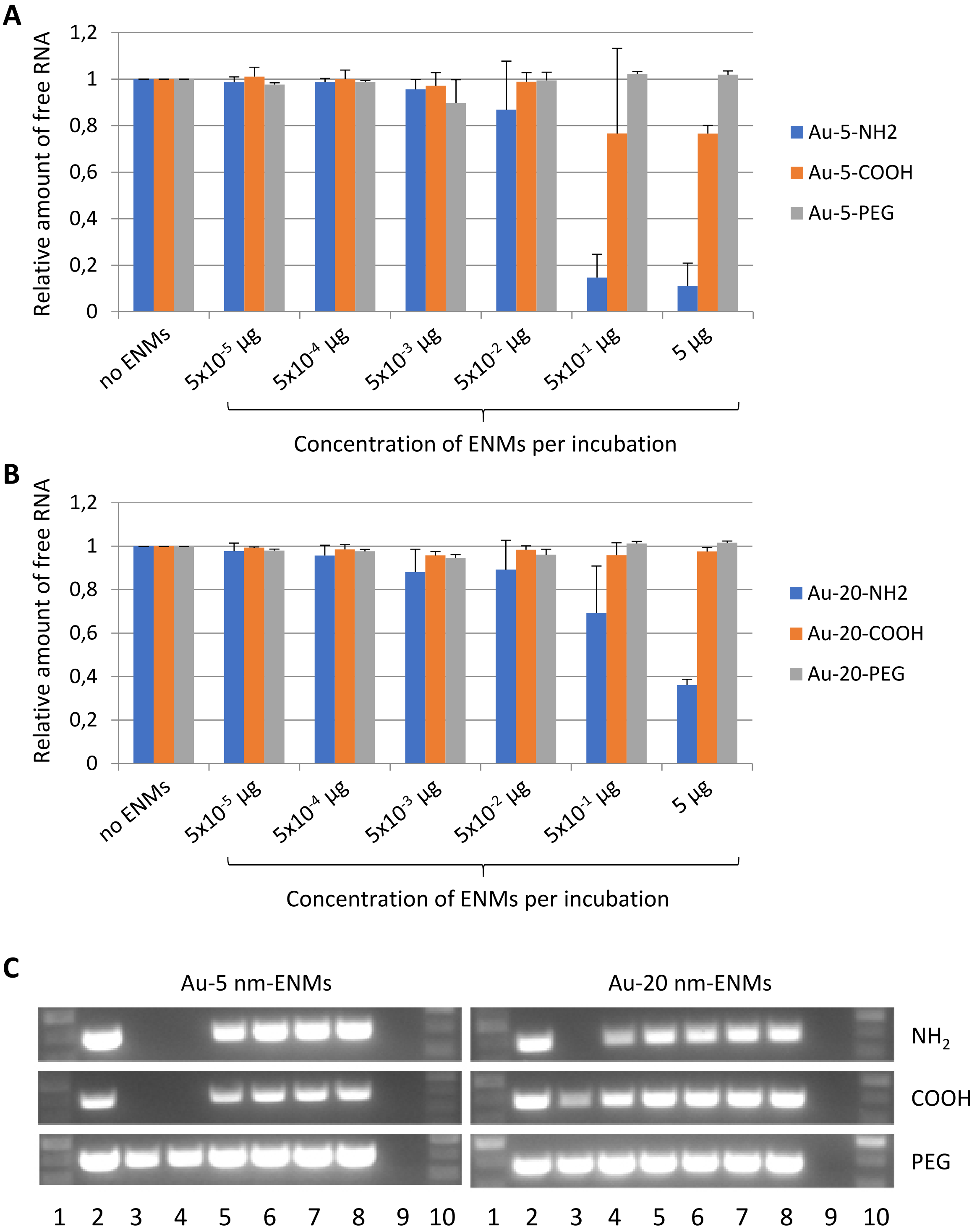

Figure S11. Nucleic acid corona studies. Free RNA left in solution after incubation with different amounts of (A) Au-5 nm or (B) Au-20 nm as compared to the starting amount of RNA. (C). Agarose gel electrophoresis of PCR amplifications of the *LDLR* gene after incubation with different amounts of Au NPs. Lanes 1) and 10) 100 bp ladder, 2) DNA w/o Au-NPs, 3-8) 50 ng DNA incubated with 5, $5x10^{-1}$, $5x10^{-2}$, $5x10^{-3}$, $5x10^{-4}$, and $5x10^{-5}$ µg of Au-NPs, respectively, and 9) no template control. Results shown are from 15 min incubations on ice.
